# Two-step model of paleohexaploidy, ancestral genome reshuffling and plasticity of heat shock response in Asteraceae

**DOI:** 10.1093/hr/uhad073

**Published:** 2023-04-19

**Authors:** Xiangming Kong, Yan Zhang, Ziying Wang, Shoutong Bao, Yishan Feng, Jiaqi Wang, Zijian Yu, Feng Long, Zejia Xiao, Yanan Hao, Xintong Gao, Yinfeng Li, Yue Ding, Jianyu Wang, Tianyu Lei, Chuanyuan Xu, Jinpeng Wang

**Affiliations:** Department of Bioinformatics, School of Life Sciences, and Center for Genomics and Computational Biology, North China University of Science and Technology, Tangshan, Hebei 063000, China; Department of Bioinformatics, School of Life Sciences, and Center for Genomics and Computational Biology, North China University of Science and Technology, Tangshan, Hebei 063000, China; Department of Bioinformatics, School of Life Sciences, and Center for Genomics and Computational Biology, North China University of Science and Technology, Tangshan, Hebei 063000, China; Department of Bioinformatics, School of Life Sciences, and Center for Genomics and Computational Biology, North China University of Science and Technology, Tangshan, Hebei 063000, China; Department of Bioinformatics, School of Life Sciences, and Center for Genomics and Computational Biology, North China University of Science and Technology, Tangshan, Hebei 063000, China; Department of Bioinformatics, School of Life Sciences, and Center for Genomics and Computational Biology, North China University of Science and Technology, Tangshan, Hebei 063000, China; Department of Bioinformatics, School of Life Sciences, and Center for Genomics and Computational Biology, North China University of Science and Technology, Tangshan, Hebei 063000, China; Department of Bioinformatics, School of Life Sciences, and Center for Genomics and Computational Biology, North China University of Science and Technology, Tangshan, Hebei 063000, China; Department of Bioinformatics, School of Life Sciences, and Center for Genomics and Computational Biology, North China University of Science and Technology, Tangshan, Hebei 063000, China; Department of Bioinformatics, School of Life Sciences, and Center for Genomics and Computational Biology, North China University of Science and Technology, Tangshan, Hebei 063000, China; Department of Bioinformatics, School of Life Sciences, and Center for Genomics and Computational Biology, North China University of Science and Technology, Tangshan, Hebei 063000, China; Department of Bioinformatics, School of Life Sciences, and Center for Genomics and Computational Biology, North China University of Science and Technology, Tangshan, Hebei 063000, China; Department of Bioinformatics, School of Life Sciences, and Center for Genomics and Computational Biology, North China University of Science and Technology, Tangshan, Hebei 063000, China; Department of Bioinformatics, School of Life Sciences, and Center for Genomics and Computational Biology, North China University of Science and Technology, Tangshan, Hebei 063000, China; Department of Bioinformatics, School of Life Sciences, and Center for Genomics and Computational Biology, North China University of Science and Technology, Tangshan, Hebei 063000, China; State Key Laboratory of Systematic and Evolutionary Botany, Institute of Botany, Chinese Academy of Science, Beijing 100093, China; University of Chinese Academy of Sciences, Beijing 100049, China; Department of Bioinformatics, School of Life Sciences, and Center for Genomics and Computational Biology, North China University of Science and Technology, Tangshan, Hebei 063000, China; Department of Bioinformatics, School of Life Sciences, and Center for Genomics and Computational Biology, North China University of Science and Technology, Tangshan, Hebei 063000, China; State Key Laboratory of Systematic and Evolutionary Botany, Institute of Botany, Chinese Academy of Science, Beijing 100093, China; University of Chinese Academy of Sciences, Beijing 100049, China

## Abstract

An ancient hexaploidization event in the most but not all Asteraceae plants, may have been responsible for shaping the genomes of many horticultural, ornamental, and medicinal plants that promoting the prosperity of the largest angiosperm family on the earth. However, the duplication process of this hexaploidy, as well as the genomic and phenotypic diversity of extant Asteraceae plants caused by paleogenome reorganization, are still poorly understood. We analyzed 11 genomes from 10 genera in Asteraceae, and redated the Asteraceae common hexaploidization (ACH) event ~70.7–78.6 million years ago (Mya) and the Asteroideae specific tetraploidization (AST) event ~41.6–46.2 Mya. Moreover, we identified the genomic homologies generated from the ACH, AST and speciation events, and constructed a multiple genome alignment framework for Asteraceae. Subsequently, we revealed biased fractionations between the paleopolyploidization produced subgenomes, suggesting the ACH and AST both are allopolyplodization events. Interestingly, the paleochromosome reshuffling traces provided clear evidence for the two-step duplications of ACH event in Asteraceae. Furthermore, we reconstructed ancestral Asteraceae karyotype (AAK) that has 9 paleochromosomes, and revealed a highly flexible reshuffling of Asteraceae paleogenome. Of specific significance, we explored the genetic diversity of Heat Shock Transcription Factors (*Hsfs*) associated with recursive whole-genome polyploidizations, gene duplications, and paleogenome reshuffling, and revealed that the expansion of *Hsfs* gene families enable heat shock plasticity during the genome evolution of Asteraceae. Our study provides insights on polyploidy and paleogenome remodeling for the successful establishment of Asteraceae, and is helpful for further communication and exploration of the diversification of plant families and phenotypes.

## Introduction

As the largest family of angiosperms, Asteraceae has 13 subfamilies with ~1600–1700 genera, including ~24 000–35 000 species, which account for ~10% of angiosperms [1]. Due to its high adaptive evolution ability, Asteraceae, a relatively young family, is one of the most successful plant groups with a wide variety of species, different morphology. Many Asteraceae plants have high ornamental and ecological value (e.g. *Chrysanthemum*, sunflower) [2,3], and the economic value of Asteraceae plant is also immeasurable, which can be seen in every aspect of our life. For example, lettuce (*Lactuca sativa*) is a very popular vegetable [4], sunflower (*Helianthus annuus*) is an important oil crop [2], *Stevia rebaudiana* can be used as a natural sweetener [5], and *Taraxacum kok-saghyz* can produce rubber [6]. Besides, Asteraceae plants also have a wide range of applications in medicine and health care, such as *Artemisia annua* for treating malaria [7], burdock (*Arctium lappa*) for treating wind and fever colds [8], and *Carthamus tinctorius* for being rich in linoleic acid and flavonoid contents [9]. However, some Asteraceae can bring some harm to the ecological environment, such as the *Conyza canadensis* [10] and *Mikania micrantha* [11], which are listed as invasive alien species in China. Until recently, 26 Asteraceae plants have been completed whole genome sequencing, including the typical representative plants *L. sativa* (2n = 2x = 18) [4], and *H. annuus* (2n = 2x = 34) [2]. These genome projects provide rich data materials for studying the structural diversity and function evolution of Asteraceae genomes.

Whole-genome duplication (WGD), frequently occurs in plant genomes along with the evolution of species [12–15]. In the long evolutionary history of plant, polyploidy, as an important driving force of genetic innovation, often occurs repeatedly. Following polyploidizations, the plant genomes showed intense dynamics due to the remodeling of the duplicated ancient genome [16,17]. This makes it extremely difficult to decipher the genome homology structure associated with polyploidy and species divergence events and understand the internal factors that promote the formation of species and phenotype diversity. Previous studies revealed multiple WGD events have occurred in Asteraceae family. Among these polyploidy events, apart from the core eudicot common hexaploidization (ECH) ~115–130 Mya [18,19], the best known is that a hexaploidization event ~57 Mya common to most if not all Asteraceae plants (named Asteraceae common hexaploidization [ACH]) [4,20–22], which was responsible for the successful establishment of the largest angiosperm family on the earth. Recent polyploidization events of Asteraceae also exist in some specific plant lineages, providing more opportunities for shaping of these plant genomes. For example, a tetraploidization ~29 Mya was occurred in genus *Helianthus*, *Stevia*, and *Mikania* genomes from subfamily Asteroideae (named Asteroideae specific tetraploidization [AST]) [2]. To understand the role of ancestral Asteraceae genome remodeling in the successful establishment of a large group of existing Asteraceae plants, it is necessary to identify the genomic homologies related to polyploidizations and species divergence.

Generally, plant genomes often become unstable after polyploidization [23–26]. Large changes may occur frequently, such as the interchromosomal rearrangements, intrachromosomal inversions, and genomic fractionation [27,28]. Polyploids are often divided into two forms, autopolyploid and allopolyploid, according to their different formation properties. After polyploidization events, extensive chromosome rearrangements and large-scale gene loss can lead to biased genomic fractionation in allopolyploids, but this scenario is missing in autopolyploids [29,30]. In this way, previous studies suggested that the paleopolyploidizations of *Brassica* and *Lupinus* genomes were caused by two-step duplications, respectively [30,31]. The essence of these studies is to assume that the two of three subgenomes from hexaploid are the moderately fractionated (MF1) and the most fractionated (MF2) related to the first step duplication, and the third subgenome of least fractionated (LF) related to the second duplication. For the ACH event, the researchers have no detected biased genomic fractionations, and speculated that this polyploidization event was generated from two-step duplications, depending on the comparisons of synonymous nucleotide substitution rates between tripled subgenome regions [4]. But these hypotheses about the two-step model of hexaploidization events can only be subjective guesses, and there is no substantive evidence. A recent study reconstructed the phylogenetic evolutionary relationships of main angiosperm clades through comparing the paleochromosome reshuffling patterns (PRPs) during the diversification of early angiosperms [32].This could assist in solving those evolutionary events that occurred in a short timeframe. If the ACH event of Asteraceae really exists two-step duplications, then we will be very excited to be able to detect the potential PRPs that can just distinguish the orders of two-step duplications.

Recursive polyploidizations and re-diploidizations can lead to highly dynamic changes of plant genomes, including rapid expansion and reduction of chromosome number [33–37]. The remarkable changes of chromosome number mainly generated from chromosomal rearrangements of ancestral genomes of existing species, showing a large number of chromosomal inversion, translocation, and fusion [38]. Chromosome fusion is the main way to reduce the number of chromosomes after polyploidy which includes three patterns, chromosome end-to-end joining (EEJ), nested chromosome fusion (NCF), and chromosome translocation (CT) [39]. For the extant Asteraceae plants, the numbers of basic chromosomes of different species varies greatly, ranging from n = 2 to 216 [22]. This large variation of Asteraceae chromosome numbers has attracted many studies to infer the ancestral Asteraceae karyotype. It is suggested that the basic chromosome number of diploid ancestor is most likely n = 9 or 2 [22]. These hypotheses are based on the comparison and induction of the basic chromosome number of the existing Asteraceae plants, lacking the consideration of the ancient genome rearrangement. The seven chromosomes in eudicot common ancestor (ECA) has been demonstrated in previous study [18]. Asteraceae plants underwent at least two genome-wide triplications (ECH and ACH events) from ECA, but the extant genomes only have smaller chromosome numbers, far less than the expected chromosomes (7 × 3 × 3 = 63). For example, the *L. sativa* has only nine gametic chromosomes. This indicates that the *L. sativa* and other Asteraceae plants experienced large-scale chromosomal rearrangements, as demonstrated in previous study that is inferred the genomes of *L. sativa*, *H. annuus*, and *Cynara cardunculus* have large number of chromosome fusions and fissions [2]. Currently, although the paleohistory of chromosome evolution of Asteraceae has been preliminarily speculated, the ancestral karyotype and chromosome evolutionary trajectories of Asteraceae plants, remains poorly understood.

In this study, by further improving our previously developed genome analysis pipeline [16], we compared 11 genomes from 10 genera of the family Asteraceae and reference genome *Vitis vinifera*. We provided clear evidence for the “two-step” duplications of hexaploidization in Asteraceae. Based on the identified genomic homologies associated with polyploidization and species divergence events, we constructed a multigenomic alignment framework for Asteraceae with a focus on *L. sativa* and *H. annuus*. According to the cross-species genomic alignment, we compared the fractionation patterns of polyploidizations produced subgenomes. Importantly, we constructed the ancestral karyotypes of key evolutionary nodes and inferred the evolutionary trajectories of chromosomes during the diversification of Asteraceae, revealing the flexible reshuffling of ancient Asteraceae. As an exploration of biological functions, we studied the plasticity of important trait genes affected by paleogenomic remodeling during the polyploidizations and (re)diploidizations, taking the Heat Shock Transcription Factors (*Hsfs*) of Asteraceae as a representative gene family.

## Results

### Paleoployploidization histories of Asteraceae genomes

**Figure 1:**
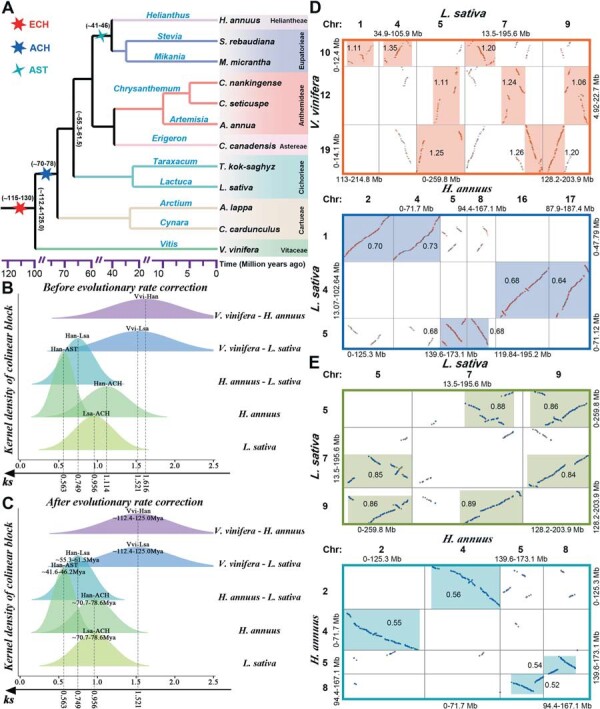
Inference of polyploidization events in the genomes of the studied Asteraceae. **(A)** Phylogenetic tree of some species of Asteraceae. Different colored stars indicate different multiploidy events, ECH: the core eudicot common hexaploidization, ACH: hexaploidization shared by most if not all Asteraceae plants, AST: the Asteroideae specific tetraploidization. **(B)***Ks* distributions of gene pairs in syntenic blocks among compared genomes. **(C)** After evolutionary rate correction, *Ks* values distributions and times of the key evolutionary events. **(D)** Specific intergenomic synteny blocks dotplots among the genomes of *V. vinifera*, *L. sativa*, and *H. annuus*. **(E)** Specific synteny blocks dotplots among the genomes of *L. sativa* with *L. sativa*, and *H. annuus*. The *Ks* median of gene pairs in homologous genomic regions were represented near the highlighted boxes.

To clearly understand the ancient polyploidization histories of Asteraceae plants, we performed synteny analysis on 11 genomes from 10 genera of Asteraceae and the reference genome *V. vinifera* (Fig. 1A). Among these genomes, we found that the Asteraceae plants usually have more syntenic genes than the *V. vinifera*. Meanwhile, we also found greater number of syntenic genes in the genomes of *H. annuus*, *M. micrantha* and *S. rebaudiana* in subfamily Asteroideae than the other Asteraceae plants (Supplementary Table S1). It is mainly because all of these Asteraceae plants experienced an additional ACH event after the ECH, and the Asteroideae plants affected by further round of AST event. We then described the synonymous nucleotide substitution rate (*Ks*) distributions of syntenic genes among Asteraceae genomes, which were related to the key evolutionary events (Fig. 1B and Supplementary Table S2). The *Ks* distribution peaks of syntenic gene pairs in *L. sativa* and *H. annuus* genomes showed two peaks approximately at 0.95, and 1.1, respectively, which were related to the ACH event. In addition to that, we also found another younger *Ks* peak in *H. annuus* approximately at 0.56, corresponding to the genome-wide doubling event AST (Fig. 1B and Supplementary Table S2). We determined the *Ks* peaks related to the species divergence events of *V. vinifera-H. annuus*, *V. vinifera*-*L. sativa*, and *L. sativa*-*H. annuus*, and found that these peaks were located at ~1.61, ~1.52, and ~ 0.56, respectively. These *Ks* peaks of syntenic genes among genomes can provide great convenience for the identification of homologous genomic regions associated with the polyploidization and speciation events.

Then, we identified the orthologous and paralogous genomic regions among the *V. vinifera* and Asteraceae plants. Genomic comparisons between the *V. vinifera* and *L. sativa*, we found that one genomic region in *V. vinifera* matched to three orthologous genomic regions in *L. sativa* (Fig. 1D and Supplementary Figs S1, S2). Comparing the *H. annuus* and *L. sativa* genomes, we identified one genomic region of the *L. sativa* that matched to two orthologous genomic regions in *H. annuus* (Fig. 1D and Supplementary Figs S3, S4). Intragenomic comparisons of the genomes of *L. sativa* and *H. annuus*, we found one to two matched paralogous regions in *L. sativa* and one to one paralogous region in *H. annuus*, respectively (Fig. 1E and Supplementary Figs S5-S8). Further, intergenomic comparisons of the *L. sativa* with other 7 Asteraceae genomes (*A. annua*, *C. canadensis*, *C. cardunculus*, *Chrysanthemum nankingense*, *Chrysanthemum seticuspe*, *A. lappa* and *T. kok-saghyz*), and the *H. annuus* and other two Asteroideae genomes (*M. micrantha* and *S. rebaudiana*), we found that they all presented the same orthologous ratio of 1:1 (Supplementary Figs S9-S21). These results indicated that the 11 Asteraceae genomes commonly experienced ACH event, and the *H. annuus*, *M. micrantha* and *S. rebaudiana* of Asteroideae experienced a further round of tetraploidization event AST, supporting that the polyploidizations of Asteraceae reported in the previous studies [2,40] (Fig. 1A and Supplementary Fig. S22). Besides, we redated the times of the polyploidization and key species divergence events among Asteraceae plants, according to the event related *Ks* distribution correction (Fig. 1C and Supplementary Table S3). We inferred that the divergences of *Vitaceae*-Asteraceae and Asteroideae*-*other Asteraceae plants were ~ 112.4–125.0 and ~ 55.3–61.5 Mya, respectively, and estimated that the ACH and AST events were ~ 70.7–78.6 and ~ 41.6–46.2 Mya, respectively.

### Event-related homologous gene framework across Asteraceae plants

Based on the identified the orthologous genomic regions among the selected representative plants *L. sativa* and *H. annuus* of Asteraceae and *V. vinifera* (Supplementary Figs S1, S2, S16, S17 and Supplementary Tables S4, S5), we identified the homologous genes related to the species divergence and polyploidization events. We found 9955 and 12 097 orthologs between *V. vinifera* and two Asteraceae genomes, respectively, and 18 517 orthologs between *L. sativa* and *H. annuus* genomes (Supplementary Tables S6, S7). Then, we identified the ECH, ACH, and AST events produced paralogs in three considered genomes (Supplementary Table S8). For example, we identified 2255 paralogous gene pairs involving 3870 genes in *L. sativa*, and 2041 paralogous gene pairs involving 2829 genes in the *H. annuus* that are produced by ACH event. It worth noted that an obvious difference in the number of ACH produced paralogous genes between *L. sativa* and *H. annuus*, with 36.8% more in *L. sativa* than in *H. annuus*. The most likely explanation is that *H. annuus* experienced more paleochromosomal rearrangements after further AST event, resulting in more gene loss.

**Figure 2:**
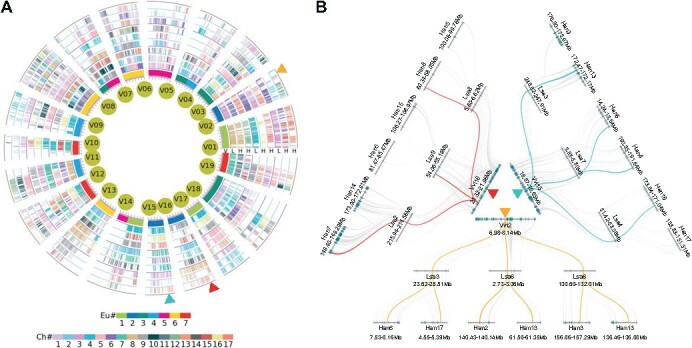
Multigenomic alignment framework and microsynteny comparison of studied genomes. **(A)** Multigenomic alignment of *L. sativa* and *H. annuus*, with the *V. vinifera* chromosomes as reference in the innermost circle. The other circles represent the orthologous genes of the *V. vinifera* genes in the genomes of *L. sativa* and *H. annuus*, with the short lines represent genes. The *V. vinifera* genes were colored using the seven ancestral eudicot chromosomes (Eu#), and their orthologs in Asteraceae genomes were color coded with the chromosome number (Ch#). **(B)** Microsyntey genomic regions from a tripled ancestral eudicot chromosome in panel A. Highlighted lines (cyan, yellow, and red) show the orthologous relationship of genes between genomes. Chromosomal region lengths are displayed in Mb.

Based on the genomic homology between two representative Asteraceae plants and *V. vinifera*, we constructed a multiple genome alignment table with *V. vinifera* as the reference genome to store the event-related homologous gene information (Supplementary Table S9). We first displayed all the *V. vinifera* genes into the first column of preset table, and then added the orthologous genes in Asteraceae genomes column by column. Since the ACH event produced three paralogous genes in *L. sativa*, each of three *V. vinifera* genomic homologs generated from ECH has three orthologous genes in *L. sativa*. In addition, each *L. sativa* gene has another two orthologous genes in *H. annuus* generated from AST event. If the orthologous genes of a queried *V. vinifera* gene that have been lost or translocated in considered Asteraceae genome, we used a dot to fill in the corresponding location of alignment table. Lastly, we constructed a multiple genome alignment table with 30 = (1 + 3 + 6) × 3 columns (Fig. 2A and Supplementary Fig. S23). Combined with whole and local genome alignment analysis, it was shown that large-scale gene loss occurred in *H. annuus* after the divergence of *L. sativa* and *H. annuus* (Fig. 2A, 2B). From this table, each gene that is focused on can be searched to locate its origin and whether it is missing in existing species.

### Allohexaploidy nature of ACH/AST events and two-step duplication model of ACH

Genomic fractionation is often characterized by widespread gene losses and translocation after polyploidization [41]. Here, we calculated the gene loss or translocation ratios in *H. annuus* and *L. sativa* relative to the reference genomes, and examined the fractionation patterns of ACH and AST events produced subgenomes, respectively. For example, we found that 50.3% (702/1399) and 52.8% (738/1399) of genes in *V. vinifera* chromosome 1 were absent from both syntenic locations in the *H. annuus* and *L. sativa* genomes, respectively (Supplementary Tables S10, S11). Using the *L. sativa* genome as a reference, we found that 63.9% (2471/3868) genes from chromosome 1 of *L. sativa* have lost in *H. annuus* genome (Supplementary Table S12). In a similar manner, compared to the *V. vinifera* genome, we found 32.9% (7779/23647) of genes lost in *L. sativa* and 33.6% (7950/23647) of genes lost in *H. annuus* (Supplementary Tables S10, S11). Furthermore, we explored the gene loss manners in *H. annuus* and *L. sativa* genomes. By referring to the genome of *V. vinifera*, we found that the length of random gene losses approximately followed a geometric distribution in *H. annuus* and *L. sativa*, with expansion parameters of 0.23 and 0.29 (Supplementary Fig. S24A, S24B and Supplementary Table S13), respectively. The goodnesses of fit values were 0.9820 and 0.9897, and the P values (*F* test) were 0.9299 and 0.9265, respectively (Supplementary Table S13). Approximately half of the runs of genes were 15 or fewer, accounting for 49.0% and 57.8% of all lost genes in *H. annuus* and *L. sativa*, respectively. Most of the runs of gene loss were 49 continuous genes or fewer, accounting for 95.2% and 95.4% of all lost genes in *H. annuus* and *L. sativa*, respectively. Besides, we identified that there were 7779 and 7950 lost genes in *L. sativa* and *H. annuus*, respectively. We carried out functional analysis of them, and showed that 7770 genes of *L. sativa* and 7941 genes of *H. annuus* matched to the *Arabidopsis* genes. Most of the lost genes were components of cells (*L. sativa* 87.4%, *H. annuus* 87.3%), more than half genes were related to biological processes (*L. sativa* 54.1%, *H. annuus* 54.1%), and only a few genes were associated with molecular functions (*L. sativa* 23.5%, *H. annuus* 23.4%) (Supplementary Fig. S24C and Supplementary Table S7). These comparisons indicated that the highly genomic fractionation exists in the genomes of living Asteraceae plants.

Next, we used sliding windows to compare the gene retention levels between ACH produced subgenomes in *H. annuus* and *L. sativa*, with the *V. vinifera* chromosomes as reference, respectively (Supplementary Figs S25, S26). We detected that almost all local genomic regions in each of these two genomes have largely divergent gene retention levels, such as the three orthologous regions of *V. vinifera* chromosome 19 were located in chromosomes 5, 7, and 9 of *L. sativa* that are retained significantly different number of genes 106, 216, and 169, respectively (P-value = 2.85e-9) (Supplementary Fig. S27). According to this quantified fractionation patterns of subgenomes, we then defined three subgenomes of the least fractionated (LF), moderately fractionated (MF1), and most fractionated (MF2) in *L. sativa*. We found 3865, 3387 and 2845 orthologs of *V. vinifera* in subgenoms LF, MF1 and MF2 from *L. sativa*, respectively, which were showed a significant divergent fractionation level (P-value <2.2 e-16) (Supplementary Fig. S28A). Along *L. sativa* chromosomes with sliding window, we also detected that the highly divergent fractionation levels between AST produced subgenomes in *H. annuus* (Supplementary Fig. S29). The autopolyploids could be having P-index >0.3, and the allopolyploids could be having P-index <0.3, as reported in previous study [42]. Therefore, we further calculated the P-index value of the *L. sativa* hexaploid ancestor to be 0.53 using the *V. vinifera* genome as a reference. When using the *L. sativa* as reference, we estimated the P-index value of *H. annuus* tetraploid ancestor was 0.44 (Supplementary Table S14). These results suggested that the ACH of Asteraceae and AST of Asteroideae are likely to be allopolyplodization events.

Here, through genomic fractionation comparisons, we suggested that the ACH may be an allohexaploidization event. Similar to the hexaploidization in *Brassica rapa* [30,43], we can assume that the ACH may be formed by a “two-step” polyploidizations, where two subgenomes first form tetraploid intermediate, and then add third subgenome by hybridization. This is different from that the two-step duplications of ACH event reported in previous study, which were inferred by the *Ks* analysis of local ACH tripled genomic regions [4]. However, we found no significant difference in the median *Ks* of anchored gene pairs among the three subgenomes LF, MF1, and MF2 in *L. sativa* (P-value = 0.9997) (Supplementary Fig. S28B), indicating that it is impossible to determine whether the ACH event of Asteraceae is consistent with the two-step duplication hypothesis by the analysis of *Ks*. Therefore, we attempted to investigate the polyploidization process of ACH event through comparisons of genomic structural features among the genomes of four Asteraceae plants (*L. sativa*, *A. lappa*, *C*. *canadensi*, and *C. cardunculus*) and *V. vinifera*.

**Figure 3:**
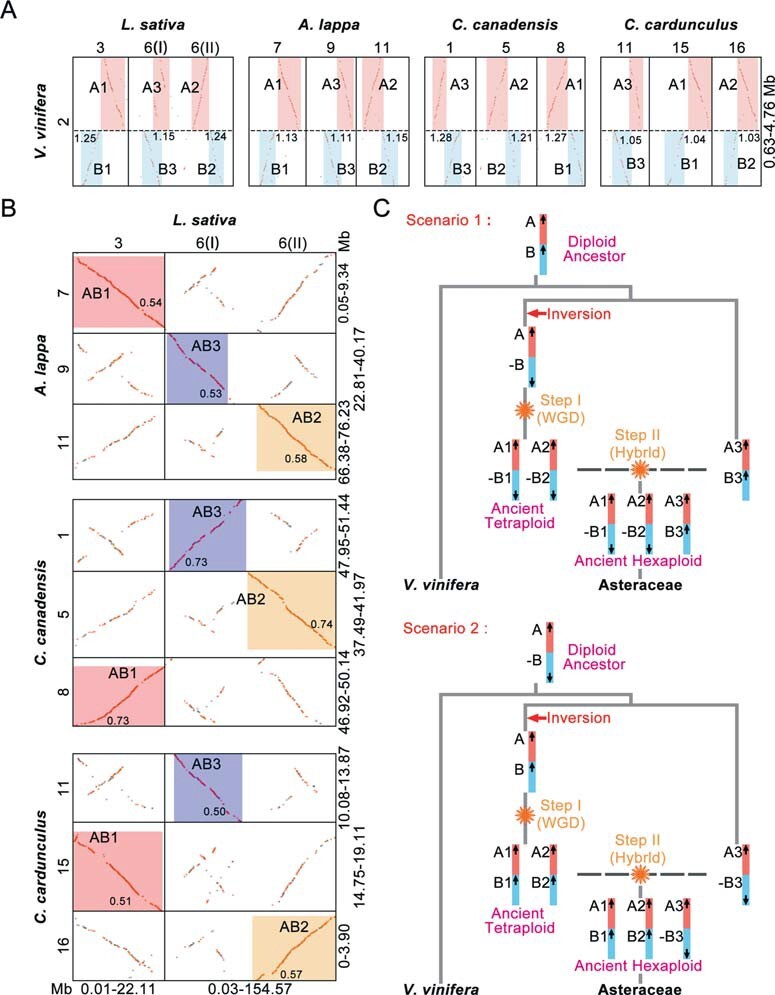
Inference of two-step duplications for ACH events. **(A-B)** Identified orthologous regions between considered genomes. The genomic regions involving the identified CRs in *V. vinifera* were defined as A and B. Highlighted boxes indicate the identified orthologous regions between genomes. The black dashed lines in genomic synteny dotplots indicate the breakage point of chromosomes involving identified CRs among the *V. vinifera* and Asteraceae genomes. The syntenic gene pairs with best and second BLAST-hits matches are plotted as red dots, and other hits are plotted as gray dots. The sizes of the involved genomic regions are displayed in Mb. The *Ks* median of syntenic gene pairs in syntenic regions were marked in the highlighted boxes. **(C)** Two scenarios of two-step duplications for the ACH event in Asteraceae. The columns with different colors indicate the chromosome regions involved in CRs, and the arrows placed inside represent the possible connection directions of the assumed chromosome regions.

Specifically, we identified a large chromosomal inversion that probably occurred during the two-step duplications of ACH event. Through previous study [18], we know that the chromosome 2 of *V. vinifera* (Vvi) was generated from one ancestral eudicot chromosome. Next, intergenomic comparisons between *V. vinifera* and *L. sativa*, we found the three orthologous regions of Vvi 2 were located in the chromosomes 3 and 6 of *L. sativa* (Lsa), which were generated from the ACH event (Fig. 3A). To clearly illustrate the genomic rearrangement involved here, we defined the regions Vvi 2 as A and B. When comparing the *L. sativa* genome to the other three representative Asteraceae genomes *A. lappa* (Ala), *C. canadensis* (Ccan) and *C. cardunculus* (Ccar), we found the orthologous chromosomes of Lsa 3, Lsa 6 (I), and Lsa 6 (II) were located in Ala 7, Ala 9, and Ala 11 of *A. lappa* (Ala), Ccan 8, Ccan 1, and Ccan 5 of *C. canadensis* (Ccan), and Ccar 15, Ccar 11, and Ccar 16 of *C. cardunculus* (Ccar), respectively (Fig. 3A, 3B). In *L. sativa*, we found that the region B of the orthologous regions of Lsa 3 and Lsa6 (II) reversed 180° compared to the region A in Lsa 3 and Lsa6 (II), but the regions A and B of Lsa6 (I) lack of this connected pattern (Fig. 3A). Interestingly, when compared the *V. vinifera* with other three Asteraceae (*A. lappa*, *C. canadensis*, and *C. cardunculus*) genomes, we also found two homologous regions of B reversed 180° in Ala 7 and Ala 11 of *A. lappa*, Ccan 8 and Ccan 5 of *C. canadensis*, and Ccar 15 and Ccar 16 of *C. cardunculus*, respectively (Fig. 3A). Further, synteny examinations revealed that the locations of the breakpoints (Vvi 2: 2.79 Mb) is similar when comparing *V. vinifera* with these four Asteraceae genomes (Fig. 3A). These genomic comparisons suggested that the two of three orthologous regions of Vvi2 in Asteraceae genomes were likely shared a (synapomorphic) chromosomal rearrangement, but missing in another orthologous region.

To further get insights into these chromosomal rearrangements (CRs), we reconstructed the evolutionary history of CRs following the ACH in extant Asteraceae plants (Fig. 3C). We first hypothesized that there are two possible connection patterns (CPs) in the regions A and B involving CR in *V. vinifera* genome: (A)-(B) and (A)-(-B). When the CP of Vvi 2 is (A)-(B), the three orthologous regions of Vvi 2 in Asteraceae exhibiting the CPs as (A1)-(-B1), (A2)-(-B2), and (A3)–(B3), respectively. If the CP of Vvi 2 is (A)-(-B), the three orthologous regions of Vvi 2 in Asteraceae exhibiting the CPs as (A1)–(B1), (A2)–(B2), and (A3)-(-B3), respectively. For these two scenarios, the CP of the regions A1, B1, A2, and B2 involving in CRs are differ from that the Vvi 2 and orthologous regions of A3 and B3 having same CPs, confirming that two of three orthologous regions of Vvi 2 in Asteraceae sharing a CR, but missing in another set of orthologous region. Therefore, we suggested that this CR occurring in a diploid ancestor of Asteraceae, then following a WGD, producing an ancient tetraploid, which was hybridized with a closely related diploid ancestor to form the present ancient hexaploid of Asteraceae (Fig. 3C). Besides, according to the identified fractionated subgenome regions, we found that Lsa 3, Lsa 6 (I), and Lsa 6 (II) were related to the subgenomes LF, MF2, and MF1, respectively (Supplementary Fig. S30). Therefore, to explain the duplications of ACH event in Asteraceae, we proposed a two-step duplication model, which was that the subgenomes LF and MF1 forming a tetraploid as the first step and then hybridized with subgenome MF2 as the second step forming the current hexaploid of Asteraceae (Fig. 3C).

### Ancestral karyotypes and evolutionary trajectories of chromosomes

**Figure 4:**
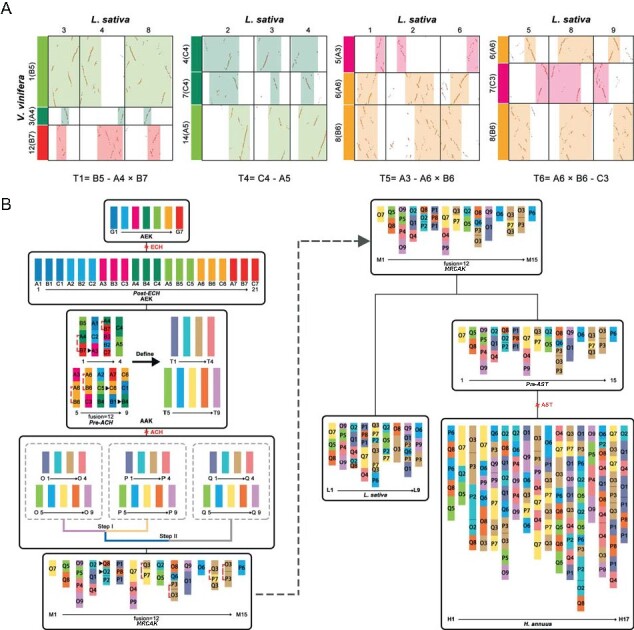
Construction of the ancestral genome and the chromosome evolution trajectories of the Asteraceae. **(A)** Local syntenic dotplots between the *V. vinifera* and *L. sativa* genomes. The 9 inferred proto-chromosomes in the ancestral Asteraceae karyotype (AAK) are represented by T1–9. In the parentheses, A, B, and C represent the three ECH produced subgenomes, and the numbers indicate the 7 ancestral eudicot chromosomes. The fusion pattern was illustrated at the bottom of the dotplot, with the “-” indicating chromosomal fusions and “×” indicating reciprocal chromosomal translocations. **(B)** The evolutionary trajectories of the most recent common ancestral karyotype (MRCAK) of *L. sativa* and *H. annuus*. G1–7 represent the ancestral eudicot chromosomes (pre-ECH). O, P, and Q represent the three ACH-produced subgenomes, and the M1–15 indicate the inferred MRCAK chromosomes. The black triangle indicates that the identified nonreciprocal chromosomal translocations (NCTs). The red dashed line indicates that the identified reciprocal chromosomal translocations (RCTs).

Ancestral chromosomes reconstruction in Asteraceae can help to reveal the evolutionary trajectories of the extant Asteraceae genomes and the possible effects of CRs. Here, we constructed the most likely ancestral Asteraceae karyotype (AAK, pre-ACH event) and the chromosome evolutionary trajectories from the ancestral eudicot karyotype (AEK) to extant genomes. With the *V. vinifera* as the reference, we inferred the AAK when ignoring the small genomic inversions and minor deletions. First, after the ECH, the 7 AEK chromosomes [18] triplicated to form 21 chromosomes (post-ECH karyotype) which were defined as A1–7, B1–7, and C1–7, respectively (Fig. 4B). Then, we inferred that the 21 chromosomes in post-ECH karyotype evolved into 9 proto-chromosomes T1–9 of AAK, which were including 11 EEJ, 1 NCF, 2 reciprocal chromosomal translocations (RCTs), and 3 nonreciprocal chromosomal translocations (NCTs). For example, the entire ancestral chromosomes C4 (Vvi4 and Vvi7) and A5 (Vvi14) of AEK were fused together with NCF pattern in the *L. sativa* genome, and located in the three paralogous chromosomes Lsa 2, Lsa 3, and Lsa 4. This connection patterns also detected in *H. annuus* genome (Supplementary Figs S30, S31, S32), thus we inferred that this fusion event occurring in the common ancestor of Asteraceae before the ACH named as T4. Similarly, the other 8 proto-chromosomes of AAK were inferred (Fig. 4A and Supplementary Fig. S31). Then, after the ACH, the nine AAK chromosomes were tripled as 27 chromosomes O1-O9, P1-P9, and Q1-Q9 (post-ACH karyotype), which were corresponding to the defined subgenomes MF1, MF2, and LF, respectively (Fig 4B and Supplementary Fig. S30). According to the inferred post-ACH karyotype, we also determined the orthogonal regions of paleochromosomes in post-ACH karyotype in the *L. sativa* and *H. annuus* genomes (Fig. 4B and Supplementary Figs S27, S33).

In addition, we inferred the most recent common ancestral karyotype (MRCAK) of Asteraceae using the representative genomes of *L. sativa* and *H. annuus*. Here, we inferred that the 27 chromosomes in post-ACH karyotype evolved into 15 proto-chromosomes M1-M15 in MRCAK involving 10 EEJ, 2 NCF, 2 RCTs, and 2 NCTs (Fig. 4B and Supplementary Fig. S34). For example, the entire ancient chromosomes Q5 and Q8 in post-ACH karyotype were fused together with EEJ pattern and corresponding to the chromosomes 1 of *L. sativa*. This connection pattern also detected in *H. annuus* (Fig. 4B and Supplementary Fig. S34), suggesting that this fusion event occurring in the MRCAK and the fused chromosome named as M4. Eventually, we identified 12 chromosome fusions reduced the chromosome number of the MRCAK from 27 of post-ACH karyotype into 15 chromosomes (Supplementary Fig. S34). Subsequently, we revealed the evolutionary trajectories of chromosomes from MRCAK to *L. sativa* and *H. annuus* karyotypes, respectively. For *L. sativa*, 15 chromosomes of MRCAK through 5 EEJ, 1 NCF, 1 RCT, and 4 NCTs evolved into the exist 9 chromosomes (Supplementary Fig. S35). After the *L. sativa* and *H. annuus* divergence, *H. annuus* experienced an additional AST event, then through 8 EEJ, 5 NCF, 2 RCTs, and 27 NCTs evolved into the exist 17 chromosomes (Supplementary Fig. S35). In summary, we constructed the ancestral karyotypes of Asteraceae, and revealed the highly flexible remodeling of ancient genome during the polyploidizations and (re)diploidizations in Asteraceae.

### Plasticity of the *Hsf* genes in Asteraceae

**Figure 5:**
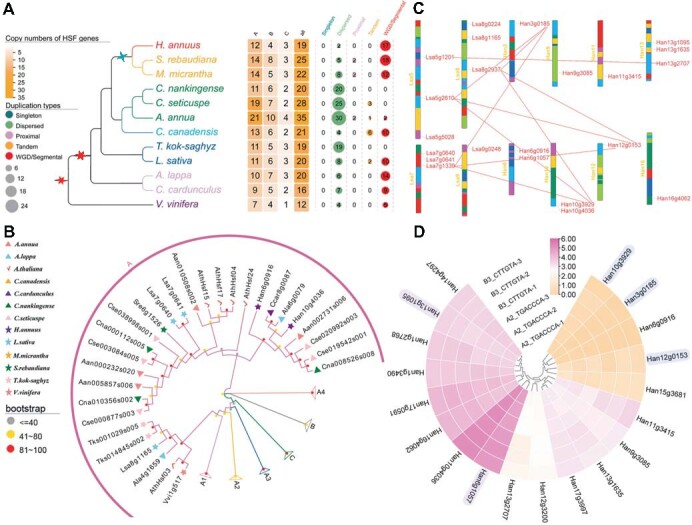
Identification and evolution analysis of *Hsf* genes in the genomes of Asteraceae and outgroup *V. vinifera*. **(A)** The copy numbers and duplication types of *Hsf* genes in 11 Asteraceae and *V. vinifera* genomes. **(B)** The phylogenetic tree of *Hsf* genes in 11 Asteraceae and *V. vinifera*, *A. thaliana* genomes. The different symbols with different colors indicate the *Hsf* genes from different genome, and the “A” presents the A subgroup of *Hsf* genes. The detailed phylogenetic trees of genes from subgroups A1, A2, A3, A4, B, C were showed in the supplementary Figure S36. **(C)** The distributions of *Hsf* genes in *L. sativa* and *H. annuus* chromosomes. The lines colored by red indicate the orthologous genes between genomes. **(D)** The expression pattern of the *Hsf* proteins in *H. annuus* genome. The gradual change of gene expression level from low to high was presented by the change from yellow to purple, the number shows the specific expression values of the proteins, and the highlighted gene names represent the ACH-produced paralogous genes.

*Hsfs* are central regulators of heat responses in all eukaryotes, such as metabolic changes. Apart from that, *Hsfs* genes have acquired broader roles in growth regulation and biotic stress responses [44]. Here, to explore the evolution of *Hsf* genes in Asteraceae, we identified 281 *Hsf* genes in *V. vinifera* and 11 Asteraceae genomes. We found the 12 *Hsf* genes in *V. vinifera* were less than that in Asteraceae (mean of all genomes: 22) (Fig. 5A and Supplementary Table S15), which shows that the *Hsf* genes largely expanded in Asteraceae. The *Hsf* genes were then classified into three subgroups A, B, and C, based on the classification of *Hsf* gene functions in *Arabidopsis thaliana* [45] (Fig. 5A, 5B). To explore the expansion patterns of different subgroups, we examined the duplication types of *Hsf* genes. We discovered that the most of *Hsf* genes in *C*. *nankingense*, *C*. *seticaspe*, *A*. *annus* and *T. kok-saghyz* were duplicated by dispersed, and most of *Hsf* genes in other studied genomes were duplicated by whole-genome or segment duplication (Fig. 5A and Supplementary Table S16). Further, we analyzed the phylogenetic tree of *Hsf* genes, and found that both of the polyploidization events and tandem provide the genetic basis for the expansion of *Hsf* genes, but the independent gene loss following repetitive polyploidizations promoted the diversity of family genes (Fig. 5B and Supplementary Fig. S36).

Next, based on the identified CRs that occurred during the diversification of Asteraceae, we placed the *Hsf* genes on the chromosomes of Asteraceae and *V. vinifera* to explore the relationship between CRs and *Hsf* gene evolution (Supplementary Figs S37-S41). We found many *Hsf* gene clusters distributed in studied genomes, such as a gene cluster including 4 *Hsf* genes on chromosome 3 of *C. canadensis* (Supplementary Fig. S41), suggesting that the formation of clusters play an important role in the expansion of *Hsf* genes. Furthermore, intergenomic comparisons of these genomes, we detected that the clusters of one species in another frequently display a decrease in the number of genes associated with CRs (Fig. 5C). For example, the three ACH-produced paralogous chromosomes 5 (*Lsa5g2610*), 7 (*Lsa7g1339*), and 8 (*Lsa8g0937*), which were related to the ancestral CR at the base of *L. sativa*, only have 1 gene, respectively. In *H. annuus*, the orthologous cluster of the three genomic regions on chromosomes 10, 12, and 3 have two genes (*Han10g3929*, *Han10g4036*, *Han12g0153* and *Han3g0185*) (Fig. 5D). These results implied that during the paleogenomic reshuffling, the CRs change the plasticity of *Hsf* genes by inhibiting family expansion.

Moreover, we identified the *Hsf* gene structures motif, coding sequence (CDS), and untranslated region (UTR) in order to further explore the structural diversity of *Hsf* genes. In general, we found that the *Hsf* genes in each group have the similar structures (Supplementary Figs S42, S43). After careful observation, we detected the differences of motifs between some members, some genes underwent a loss of motif, such as *Cna004128s001* from group A3 missing the motif4 (Supplementary Figs S42, S43). For motif5, it was specific to those genes from group B. These results showed that the gene structural variations (GSVs) of *Hsf* genes exhibited another salient evolutionary trait that might be associated with its specific function. Next, to explore the potential functional impacts of structural diversity of *Hsf* genes, we analyzed the expression patterns of *Hsf* proteins in *H. annuus* and *L. sativa* genomes (Fig. 5D, Supplementary Fig. S44 and Supplementary Tables S17, S18). We found that the ACH-produced paralogous genes *Han6g1057* expressed highly in each tissue, the *Han3g0185* not expressed in all tissues, and the *Han12g2707* expressed at different levels in different tissues (Fig. 5D), suggesting that the expression patterns of duplicated *Hsf* proteins show strong expression diversity. Further, for the genes *Han6g1057* and *Han13g1095* with motif7, the expression level in each tissue is higher than the genes *Han10g3929*, *Han3g0185* and *Han12g0153* without motif7 (Fig. 5D and Supplementary Figs S42, S43). For the gene *Han6g1057* with motif8, the expression level in each tissue is higher than the gene *Han13g1095* without motif8 (Fig. 5D and Supplementary Figs S42, S43). In summary, the motif 7 and 8 might take a significant role in the expression pattern of *Hsf* genes. Besides, for the ACH-produced paralogs *Han10g3929*, *Han3g0185* and *Han12g0153*, the expression levels are lowered than other *Hsf* genes, implying that the *Hsf* duplicated genes generated by polyploidization events appearing neo/subfunctionalization (Fig. 5D). Based on the above analysis results, we summarized that the expansion of *Hsf* gene families enabled heat shock plasticity during the genome evolution of Asteraceae.

## Discussion

### Polyploidization events and multigenomic alignment resources in Asteraceae

Recursive polyploidizations have occurred frequently in plant genomes and provided tremendous genetic basis for genome functional innovation [46–49]. During polyploidizations and (re)diploidizations, the plant genomes are often accompanied by large scale gene loss and CRs, which adds complexity to the genome and creates significant challenge in identifying polyploidy events [16,50]. Among all identified polyploidizations, the hexaploidization events are relatively rare compared with ancient tetraploidization events. After the ECH event, the many Asteraceae genomes underwent one whole-genome triplication event (~70.7–78.6 Mya), and in particular several studies suggested that this triplication event was common in Asteraceae genomes [1,2,4,8,40]. We confirmed that the ACH event commonly occurred in the studied Asteraceae after the ECH event, which is consistent with previous reports [2,4,51], and revealed some species of Asteroideae experienced a further round of tetraploidization event AST. We also inferred that the ACH event occurring at ~70.7–78.6Mya, which was likely shared by all Asteraceae plants after the Asteraceae split with *V. vinifera* at ~123.7 Mya [52].

Plants have undergone recurrent polyploidizations and genome remodeling during the process of evolution, resulting in complex genome structures of plants [53]. This makes it challenging to interpret their homologous genomic structures, comprehend the genome-generation process, and investigate the functional evolution of genes associated with significant features. The identification of genes associated with evolutionary events may aid in reflecting the influences of such polyploidizations on genome expansion and differentiation. Here, using reference genome (*V. vinifera*), we constructed an event-related multigenomic alignment framework with *L. sativa* and *H. annuus* as the core. This list displays how pairs of homologous genes arise and diverge, and whether gene loss occurs after certain doubling events, providing important paleogenome genetic information for uncovering genes, gene families, regulatory pathways, and evolutionary and functional innovations for economically important traits in agriculture. This work is valuable for analyzing the layers of homologous regions following the recursive polyploidizations, generating the paralogs and orthologs lists, and connecting these homologs to species divergence events and polyploidizations.

### Genomic fractionation and two-step duplications

Polyploidy plays a key role during plant diversification and contributes to artificial selection during the domestication of crops [42,46,47, 54–57]. Polyploidization events can divide into two types: allopolyploidization and autopolyploidization event. Allopolyploids are produced by crossing different genomes, and the autopolyploids are produced by doubling the genome in a cell [58,59]. Previous studies have suggested that the genomic fractionation caused by tens of million years may not be able to distinguish the allopolyploids and autopolyploids [42,60,61]. In this study, the ACH event of Asteraceae is a hexaploidization similar to the best-known ECH (*gamma*) event which covered 75% angiosperms [62,63] including Asteraceae plants. Here we compared the ACH-produced subgenomes, and revealed that the biased genomic fractionation existed between them. But instead of comparing the three subgenomes within *L. sativa*, as in previous *L. sativa* study [4]. Here, we analyzed the subgenomes with the help of outgroup *V. vinifera* and detected that the extensive imbalances of retention and loss of ancestral eudicot genes between the three ACH-produced subgenomes in *L. sativa*. And with the help of the statistical indicator P-index [42], we further suggested that ACH and AST events were likely two allopolyplodization events.

In previous studies, the researchers proposed that the ECH event, *Brassica* common hexaploidization and *Lupinus* lineage specific hexaploidization are also showed allopolyploidy nature, while the observed patterns of triplicated genomes were explained by a two-step duplication model [29–31,43]. The ACH-produced subgenomes of *L. sativa* underwent biased genomic fractionation after hexaploidization, suggesting that the formation process of ACH in Asteraceae may be similar to that of *B. rapa* hexaploidization, that is, subgenomes MF1 and MF2 form a tetraploid as the first step, and then hybridized with subgenome LF to form hexaploid as the second step [30,43]. In order to verify the two-step duplications hypothesis of ACH, we first calculated the *Ks* median values of anchor gene pairs between the three subgenomes, but found no significant difference between *Ks* values of subgenomes (Supplementary Fig. S28B). Then, through the comparisons of GSV features, we identified a large chromosomal inversion that probably occurred during the two step duplications of the ACH event. This supported that the two-step duplications process for the ACH, with the subgenomes LF and MF1 formed a tetraploid as the first step and then hybridized with the subgenome MF2 to form a hexaploid as the second step. Our findings revealed that the most recently added subgenome would be MF2 rather than the dominant subgenome (LF), and rejected the previous hypothesis [29,31,43].

### Highly dynamic remodeling of Asteraceae paleogenome

Ancestral chromosome karyotype reconstruction is crucial for establishing the phylogenetic position of species and illuminating the effects of various polyploidy events on genomic diversification [50]. Based on parsimony phylogenomic analysis, we could find the date of occurrence for relative genomic changes and infer the karyotype evolution [36]. Asteraceae species shared another hexaploidization event (ACH) after ECH event. If the fusions of chromosomes are not considered, the number of chromosomes in Asteraceae will reach to 7 × 3 × 3 = 63. But the representative species *L. sativa* contains only nine chromosomes, indicating that the chromosomal rearrangements occurring dramatically in Asteraceae species. Previous studies have suggested that n = 9 was the most likely chromosomal base number in Asteraceae and speculated that Asteraceae ancestors may have only 9 ancestral chromosomes [22], but this lacked clear evidence of genomic structure. In this study, according to the previously proposed theory of telomere-centric genome repatterning [39], we constructed the Asteraceae ancestor has 9 chromosomes before ACH event, and verified the hypothesis that the ancestors had 9 chromosomes. The reconstruction of the AAK provides a reference for studying the origin and evolution of extant Asteraceae. This allows us to better understand the evolutionary history of Asteraceae at the chromosome level. Meanwhile, we also deduced the MRCAK of *L. sativa* and *H. annuus* following the ACH event, which filling the gap in the evolution of the chromosome karyotype of Asteraceae family. Among them, we inferred that the chromosome rearrangements of *L. sativa* and *H. annuus* from AEK to modern chromosomes were 30 and 37 chromosome fusions, respectively. These chromosome fusion patterns were mainly EEJ and similar to those of most eudicots, such as the *V. vinifera* [18]. In summary, we constructed the ancestral karyotypes of Asteraceae providing an important reference for inferring the chromosomal evolutionary trajectories, and revealed the highly dynamic remodeling of ancient genome during the polyploidizations and (re)diploidizations in Asteraceae.

### Strongly plasticity of heat shock response in Asteraceae

*Hsf* is an important gene family for resistance to heat stress, which can specifically recognize and binds to the heat shock element on the promoter of the heat shock protein in the face of heat stress. Thereby it can mediate and activate the expression of the *Hsf* gene in response to heat stress, and then mitigate the damage caused by heat stress [64]. The genomes of many organisms, including *V. vinifera* [65], Apiaceae [45], *Secale cereale* [66] and *Tigriopus californicus* [67], have been analyzed for *Hsf* genes. Here, the *Hsf* genes were identified and classified in *L. sativa*, *H. annuus* and 10 other studied genomes. In previous studies it has been suggested that the highly dynamic nature of genome structure plays an important role in the diversification of polyploid species [17,37,68,69]. In our study, we found that the strongly plasticity of the *Hsf* genes in Asteraceae, and this plasticity was closely related to the polyploidization, tandem, and dispersed duplication events. We also inferred the duplication type of *Hsf* genes and found that as the proportion of copy numbers of the *Hsf* gene (produced by WGD events) increased, the temperature range for optimal growth increased. For example, the *Hsf* gene (produced by WGD events) copy number proportions in *L. sativa* and *H. annuus* were 50% (10/20) and 89.5% (17/19) respectively, while the optimum growth temperatures were 15–25°C and 31–37°C respectively. This suggests that the *Hsf* gene produced by WGD may help to enhance the adaptive capacity of Asteraceae. In addition, we also found the duplication types detection in *C*. *nankingense*, *C*. *seticaspe*, *A*. *annus* and *T. kok-saghyz* might be influenced by the genomes which were failed to assemble at the chromosomal level. This result exemplifies the importance of high-quality genome sequencing and assembly. The phylogenetic analyses and chromosome locations of *Hsf* genes also showed that the highly plasticity of *Hsf* genes, which was affected by the duplication events and genomic reshuffling in extant Asteraceae plants.

Gene structural variations may lead to functional differences. This phenomenon is also present in *Hsf* genes of Asteraceae. Here, we found that those proteins which lost the specific motif likely resulted the lower expression of *Hsf* genes, providing a new clue to explore the plant breeding about the *Hsf* genes for improving the ability of species to withstand heat stress. Besides, our results revealing that the *Hsf* duplicated genes generated by polyploidization events appearing subfunctionalization. In summary, together with the results of structural variations and expression pattern analyses of *Hsf* genes, suggested that modifying the copy number and structure of them may greatly improve the heat tolerance of Asteraceae plants.

## Materials and methods

### Materials

The reference genome *V. vinifera* and 11 genomes from Asteraceae (*L. sativa*, *H. annuus*, *A. annua*, *C. canadensis*, *C. cardunculus*, *M. micrantha*, *C. nankingense* and *C. seticuspe*, *S. rebaudiana*, *A. lappa* and *T. kok-saghyz*) used in this study were downloaded from the public databases. Supplementary Table S19 provides comprehensive genomic information. In this study, we selected *V. vinifera* as the reference genome because it has a relatively conservative genomic structure without additional polyploidization events after the eudicot common hexaploidization event [19].

### Inference of genome synteny

To infer the genome synteny, the BLASTP [70] was utilized to look for possible homologous gene pairs and set the parameters E-value <1e-5 and score > 100. Following that, the BLAST results as input for ColinearScan [71] to infer the syntenic genes among studied genomes. The maximum gap was set to 50 intervening genes, and in the BLAST results, the large gene families with 50 or more members were eliminated from the anchored gene pairs. This approach can be well adopted in the genomic synteny analysis of angiosperms and has been widely used in previous studies [16,32,72].

### *Ks* calculation and event-related distribution analysis

Calculation of *Ks* between homologous gene pairs in syntenic blocks, we performed Nei-Gojobori’s [73] algorithm and implemented with the Bioperl statistical module. In this study, the *Ks* distribution of syntenic blocks within and between genomes was analyzed using the kernel function. The kernel smoothing density function *Ks* density’s width parameter was set to 0.05 [74] when estimating the density curves of *Ks* median of syntenic gene pairs in each block using MATLAB. The curves were fitted by the fitting toolbox *cftool*’s *Gaussian* method. The *R-squared* parameter was generally set to at least 95% to evaluate the goodness of fit, and the *Ks* distributions were represented by the fewest possible normal distributions. We used the maximum likelihood estimate method to deduce the μ (*Ks* peak) from the *Ks* distribution curves, and the polyploidization and species divergence events was represented by *Ks* peaks.

### Inference of event-related genomic homology

To identify genomic homologies generated from the polyploidization and species divergence, we utilized the dotplots that integrated genomic synteny and *Ks* analysis. A dotplot for comparison within and between genomes was constructed according to the genomic locations of syntenic genes, and the anchor gene pairs with the best BLAST hit were shown as red dots. We identified event-related genomic homologies by the *Ks* median of syntenic gene pairs in blocks. Within genomes, when the *Ks* median of gene pairs in a syntenic region is roughly equivalent to the *Ks* peak associated with polyploidization, this region is referred to as paralogous region. Between genomes, when the *Ks* median of gene pairs in a syntenic region is roughly equivalent to the *Ks* peak associated with species divergence, this region is referred to as orthologous region.

### Evolutionary dating correction based on *Ks*

Plant genomes evolved at divergent evolutionary rates [30,75], making it difficult to determine the timing of key events in their evolutionary history. Here, we constructed a correction algorithm which was similar to previously applications in families Fabaceae [76], Malvaceae [72], and Cucurbitaceae [16]. The correction process includes two rounds according to different correction benchmarks. The first round of correction was based on the *Ks* distribution peaks of divergence event of Asteraceae plants with *V. vinifera* to have the same values. Assuming that the *Ks* distribution of divergence between Asteraceae plants and *V. vinifera* follows a normal distribution $X\sim \left(\mu, {\sigma}^2\right)$, we can get that the distribution between specific Asteraceae plant *i* and *V. vinifera* is ${X}_i\sim \left({\mu}_i,{\sigma}_i^2\right)$, and the Asteraceae plant *s* and *V. vinifera* with the slowest divergence is ${X}_s\sim \left({\mu}_s,{\sigma}_s^2\right)$. Then, we can further assume that the correction coefficient of Asteraceae plant *i* is ${\lambda}_i$, while the correction coefficient of Asteraceae plant *s* and *V. vinifera* would be fixed to 1. Aligning the distributions between Asteraceae plant*s* and *V. vinifera* to the have same maximum likelihood value of *Ks* peaks with that of the slowest evolving Asteraceae plant *s* resulted in

$$ \left(\frac{1+{\lambda}_i}{2}\right)\times{\mu}_i={\mu}_s, $$

and

$$ {\lambda}_i=\frac{\mu_s}{\mu_i}\times 2-1, $$

then

$$ {X}_{correction}\sim \left({\lambda}_i{\mu}_i,{\lambda}_i^2{\sigma}_i^2\right). $$

After first round of correction, the ACH-produced paralogs in Asteraceae plants still had very divergent *Ks* distributions. Therefore, we made a further round of corrections, aligning the *Ks* distribution peaks of the paralogs of Asteraceae produced by ACH with that of the slowest evolving Asteraceae. The second round of correction method was similar to the approach used in previous research of Fabaceae genomes [76].

### Presentation of gene homology associated with evolutionary events

To present the identified gene homology information associated with evolutionary events, we constructed a table to store multigenome syntenic genes across Asteraceae plants based on a reference genome (*V. vinifera*). The table filled the first column with all *V. vinifera* genes, with two additional columns filled homologs genes for each *V. vinifera* gene due to the ECH event. Then gene IDs for species of Asteraceae were added column by column based on the identified orthologous information. When the orthologous gene is missing, we replace the missing gene ID with a dot in the corresponding cell. For the Asteraceae genomes experienced ACH event, we assigned 3 × 3 columns for each Asteraceae plant. If the Asteraceae genomes have additional *N* round of tetraploidization events after the ACH, we can assign 3x3x*N*x2 columns.

### Detection of the gene loss rate

Using *V. vinifera* as a reference, we counted the genes with no orthologous genes in Asteraceae and counted the frequency of gene loss in each continuously lost region. The following formula was used to estimate the probability of missing several consecutive groups of genes:

$$ \mathrm{y}=f\left(x=k|p\right)={\left(1-p\right)}^{n-1}p $$

The fitting functions curve-fit and NumPy were run using Python scripts.

### P-index calculation and inference of paleopolyploid nature

To infer the possible nature of polyploids, we utilized a previously developed polyploidy index (P-index) to assess the degree of differentiation between polyploid subgenomes [42]. In this study, using *V. vinifera* and *L. sativa* as reference genomes, we calculated the P-indices of ACH and AST events produced subgenomes, respectively, where the number of sliding windows was 50 and the parameters were set to 0.05–1.8. The calculation formula of P-index value is as follows:

$$ \mathrm{P}- index=\sum \limits_{c=1}{w}_c abs\left[\frac{\sum{}_{i=1}^{N_c}\frac{A_i-{B}_i}{abs\left({A}_i-{B}_i\right)}\times{\delta}_i}{N_c-\delta}\right] $$

Previous studies have demonstrated that the robustness of P-index to infer the nature of polyploidy. According to the previous results, known or inferred species with paleoallopolyploid ancestors usually have P-index >0.3 [16,42,54,77]. In contrast, species with known or inferred paleoautopolyploid ancestors usually have P-index <0.3 [42].

### Reconstruction of Asteraceae paleogenome

We used the “Telomere-centric genome repatterning model” proposed in previous study [39] to infer the evolutionary trajectory of chromosomes in Asteraceae plants. The main inference steps are as follows: A) Genomic comparison analysis of the reference (*V. vinifera*) and Asteraceae genomes, we plotted the homologous gene dotplots and identified the orthologous genomic regions between the reference genome and Asteraceae genomes. B) Identifying the genomic regions that have been affected by chromosomal rearrangements in the genomes of Asteraceae and showing the connecting order of these orthologous sections. C) Constructed the AAK based on the orthologous regions of extant species. D) Comparing the genomes of Asteraceae genomes and the inferred AAK to determine the status of the MRCAK of Asteraceae. E) Comparing the genomes of Asteraceae plants with the inferred model of MRCAK, respectively, and identifying the species-specific genomic rearrangements during the evolution.

### Identification and evolutionary analyses of *Hsfs* genes

The protein sequences of *Hsfs* in *A. thaliana* were downloaded from PlantTFdb database (http://planttfdb.gao-lab.org/). Then, the *A. thaliana Hsf* sequences were used as the query to search in *V. vinifera* and eleven Asteraceae genomes by performing BLASTP software [78], with strict parameters *E-value* < 1e−5 and *Score* > 100. All *Hsfs* family members with high confidence were obtained by gene structural domain screening. The protein sequences without HSF_DNA-bind (PF00447) structural domains were removed by using the Pfam (http://pfam.xfam.org/), followed by the removal of sequences without coiled-coil using the SMART (http://smart.embl.de/smart/batch.pl), and the detection of sequences with coiled-coil using MARCOIL (https://toolkit.tuebingen.mpg.de/tools/marcoil) to detect the sequences with HR-A/B structural domains. Next, we constructed the phylogenetic tree using MEGA X software [79] with Neighbor-Joining method and 1000 bootstrap replicates. Then, based on the phylogenetic tree of *Hsfs* proteins and the classification of *A. thaliana Hsf* sequences [45], the genes were divided into different subgroups. Next, to explore the diversity of structures, the gene structure was identified. The motifs were recognized by Multiple Expectation maximization for Motif Elicitation (MEME, https://meme-suite.org/meme/tools/meme), and the maximum number of motifs detected was set to 10, while the other parameters were defaulted. The gene structure CDS and UTR and motifs of *Hsf* members were visualized by the TBtools [80].

Moreover, to visualize the distribution of gene family members on the chromosomes of each species, we have illustrated the distribution of family members on chromosomes by the Gene Location Visualize module in TBtools [80] by reading the sequence length and locations on chromosomes. Next, to explore the metabolomic regulation of *Hsfs* genes, the expression data of *H. annuus* (SRR17804688-SRR17804693) [81] and *L. sativa* (SRR9659238, SRR9659247, SRR9659244, and SRR9659241) [82] was download from the SRA database (https://www.ncbi.nlm.nih.gov/sra/). For *H. annuus*, we selected transcriptome data from seeds incubated in glass Petri dishes with polyethylene glycol (SRR17804691-SRR17804693), and water (SRR17804688-SRR17804690) moistened with cotton for 15 h at 20°C [81]. For *L. sativa*, transcriptome data from aerial tissues at four different time after light treatment [82] were selected (detailed information can be found in Table S18). We processed the raw RNA-seq reads using Trimmomatic software [83] and removed the adaptor sequences and low-quality reads with the default parameters. Then, we mapped the clean reads to the genomes by using Hisat2 software [84] with default parameters, and quantified them by using StringTie software [85] with the “-e -A” parameter. Then, the expression patterns of family genes were shown by the TBtools software [80]. The expression patterns of *Hsf* proteins within *H. annuus* and *L. sativa Hsf* proteins were visualized by TBtools [80].

## Acknowledgments

This work was funded by the National Natural Science Foundation of China (32170236 and 31501333 to J.P.W.), the Hebei Natural Science Foundation (C2020209064 to J.P.W.), and the Fundamental Research for the Hebei Province Universities (JQN2020018 to T.L.).

## Authors contributions

J.P.W. conceived the project and was responsible for the project initiation. J.P.W., X.K., C.X., and T.L. supervised and managed the project and research. Z.W., S.B., Y.F., J.Q.W., Z.Y., F.L., Z.X., Y.H., X.G., Y.L., Y.D. and J.Y.W. performed the analysis. The manuscript was organized, written and revised by J.P.W., X.K., Y.Z., All authors read and approved the manuscript.

## Data availability

All original data in this study are available through the article /Supplementary Material.

## Conflict of interest statement

The authors declare no conflicts of interest.

## Supplementary Data

Supplementary data is available at *Horticulture Research* online.

## Supplementary Material

Web_Material_uhad073
