# Supplementary material for: Two-step model of paleohexaploidy, ancestral genome reshuffling and plasticity of heat shock response in Asteraceae
Source: Hortic Res. 2023 Apr 19;10(6):uhad073. doi: 10.1093/hr/uhad073 (PMC10251138; doi:10.1093/hr/uhad073)
Supplement: Web_Material_uhad073 [file web_material_uhad073.zip › Supplementary_Figures.docx]

**Supplementary Information**

**Two-step model of paleohexaploidy, ancestral genome reshuffling and plasticity of heat shock response in Asteraceae**

Xiangming Kong1, †, Yan Zhang1, †, Ziying Wang1, †, Shoutong Bao1, †, Yishan Feng1, Jiaqi Wang1, Zijian Yu1, Feng Long1, Zejia Xiao1, Yanan Hao1, Xintong Gao1, Yinfeng Li1, Yue Ding1, Jianyu Wang1, Tianyu Lei1, 2, 3, *, Chuanyuan Xu1, *, Jinpeng Wang1, 2, 3, *

^1^Department of Bioinformatics, School of Life Sciences, and Center for Genomics and Computational Biology, North China University of Science and Technology, Tangshan, Hebei 063000, China

^2^State Key Laboratory of Systematic and Evolutionary Botany, Institute of Botany, Chinese Academy of Science, Beijing 100093, China

^3^University of Chinese Academy of Sciences, Beijing 100049, China

^†^These authors contributed equally to this study.

^*^**Corresponding author:**

Tianyu Lei, Email: [leitianyu@ncst.edu.cn](mailto:leitianyu@ncst.edu.cn); Add: 21 Bohai Road, Caofeidian Xincheng, Tangshan 063210, Hebei, China; Tel: 86-315-8805600.

Chuanyuan Xu, Email: [xuchuanyuan@ncst.edu.cn](mailto:xuchuanyuan@ncst.edu.cn); Add: 21 Bohai Road, Caofeidian Xincheng, Tangshan 063210, Hebei, China; Tel: 86-315-8805600.

Jinpeng Wang, E-mail: [wangjinpeng@ibcas.ac.cn](mailto:wangjinpeng@ibcas.ac.cn); Add: 21 Bohai Road, Caofeidian Xincheng, Tangshan 063210, Hebei, China; Tel: 86-315-8805600.

**Running Title:** Flexible reshuffling of paleogenome in Asteraceae

Content

[**Supplementary** **Figure S1. Intergenomic comparison analyses of the *Vitis* *vinifera* and *Lactuca* *sativa* genomes.** 4](#_Toc125062134)

[**Supplementary** **Figure S2.** **Intergenomic comparative analyses: Syntenic dotplot between the *V*. *vinifera* and *L*. *sativa* genomes.** 5](#_Toc125062135)

[**Supplementary Figure S3. Intergenomic comparison analyses of the *L*. *sativa* and *Helianthus* *annuus* genomes.** 6](#_Toc125062136)

[**Supplementary Figure S4. Intergenomic comparative analyses: Syntenic dotplot between the *L*. *sativa* and *H*. *annuus* genomes.** 7](#_Toc125062137)

[**Supplementary Figure S5. Intragenomic comparison analyses of the *L*. *sativa* genome.** 8](#_Toc125062138)

[**Supplementary** **Figure S6. Intragenomic comparative analyses: Syntenic dotplot within the *L*. *sativa* genome.** 9](#_Toc125062139)

[**Supplementary Figure S7. Intragenomic comparison analyses of the *H*. *annuus* genome.** 10](#_Toc125062140)

[**Supplementary Figure S8. Intragenomic comparative analyses: Syntenic dotplot within the *H*. *annuus* genome.** 11](#_Toc125062141)

[**Supplementary Figure S9. Intergenomic comparison analyses of the *L*. *sativa* and *Conyza* *canadensis* genomes.** 12](#_Toc125062142)

[**Supplementary** **Figure S10. Intergenomic comparative analyses: Syntenic dotplot between the *L*. *sativa* and *C*. *canadensis* genomes.** 13](#_Toc125062143)

[**Supplementary Figure S11. Intergenomic comparison analyses of the *L*. *sativa* and *Cynara* *cardunculus* genomes.** 14](#_Toc125062144)

[**Supplementary Figure S12. Intergenomic comparative analyses: Syntenic dotplot between the *L*. *sativa* and *C*. *cardunculus* genomes.** 15](#_Toc125062145)

[**Supplementary Figure S13. Intergenomic comparison analyses of the *L*. *sativa* and *Arctium* *lappa*.** 16](#_Toc125062146)

[**Supplementary Figure S14. Intergenomic comparative analyses: Syntenic dotplot between the *L*. *sativa* and *A*. *lappa* genomes.** 17](#_Toc125062147)

[**Supplementary Figure S15. Intergenomic homologous structure between the genomes of *L*. *sativa* and other Asteraceae (*Artemisia* *annua*, *Chrysanthemum* *nankingense*, *Chrysanthemum* *seticuspe*, and *Taraxacum* *kok-saghyz*).** 18](#_Toc125062148)

[**Supplementary Figure S16. Intergenomic comparison analyses of the *V*. *vinifera* and *H*. *annuus* genomes.** 19](#_Toc125062149)

[**Supplementary Figure S17. Intergenomic comparative analyses: Syntenic dotplot between the *V*. *vinifera* and *H*. *annuus* genomes.** 20](#_Toc125062150)

[**Supplementary Figure S18. Intergenomic comparison analyses of the *H*. *annuus* and *Mikania* *micrantha* genomes.** 21](#_Toc125062151)

[**Supplementary Figure S19. Intergenomic comparative analyses: Syntenic dotplot between the *H*. *annuus* and *M*. *micrantha* genomes.** 22](#_Toc125062152)

[**Supplementary Figure S20. Intergenomic comparison analyses of the *H*. *annuus* and *Stevia* *rebaudiana* genomes.** 23](#_Toc125062153)

[**Supplementary Figure S21. Intergenomic comparative analyses: Syntenic dotplot between the *H*. *annuus* and *S*. *rebaudiana* genomes.** 24](#_Toc125062154)

[**Supplementary Figure S22. Gene phylogenetic tree of *V*. *vinifera*, *L*. *sativa*, and *H*. *annuus*.** 25](#_Toc125062155)

[**Supplementary Figure S23. Genomic alignment of *L*. *sativa* and *H*. *annuus*.** 26](#_Toc125062156)

[**Supplementary Figure S24. Gene loss analysis of *L*. *sativa* and *H*. *annuus* genomes.** 27](#_Toc125062157)

[**Supplementary Figure S25. ACH event in *L*. *sativa* generates intersubgenome retention level balance.** 28](#_Toc125062158)

[**Supplementary Figure S26. Polyploidization event in *H*. annuus generates intersubgenome retention level balance.** 29](#_Toc125062159)

[**Supplementary Figure S27. Intergenomic homologous structure comparison analyses between *V*. *vinifera* and *L*. *sativa* genomes.** 30](#_Toc125062160)

[**Supplementary Figure S28. LF, MF1 and MF2 subgenomic gene retention numbers and inter-subgenomic *Ks*.** 31](#_Toc125062161)

[**Supplementary Figure S29. AST event in *H*. *annuus* generates intersubgenome retention level balance.** 32](#_Toc125062162)

[**Supplementary Figure S30. Ancestral chromosome fusion in the Asteraceae family inferred from *L*. *sativa*.** 33](#_Toc125062163)

[**Supplementary Figure S31. Construction of the ancestral karyotype of Asteraceae.** 34](#_Toc125062164)

[**Supplementary Figure S32. Ancestral chromosome fusion in the Asteraceae family inferred from *H*. *annuus*.** 35](#_Toc125062165)

[**Supplementary Figure S33. Intergenomic homologous structure comparison analyses between *V*. *vinifera* and *H*. *annuus* genomes.** 36](#_Toc125062166)

[**Supplementary Figure S34. Construction of the most recent common ancestral karyotype of Asteraceae.** 37](#_Toc125062167)

[**Supplementary Figure S35. Reconstruction of the chromosome evolution trajectories of *L. sativa* and *H. annuus*.** 38](#_Toc125062168)

[**Supplementary Figure S36. Inference of the expansion pattern of *Hsf* proteins by comparing the Asteraceae with the *V*. *vinifera* genes.** 39](#_Toc125062169)

[**Supplementary Figure S37. The chromosomal distributions of *Hsf* genes in *V*. *vinifera*.** 40](#_Toc125062170)

[**Supplementary Figure S38. The chromosomal distributions of *Hsf* genes in *L*. *sativa*.** 41](#_Toc125062171)

[**Supplementary Figure S39. The chromosomal distributions of *Hsf* genes in *H*. *annuus*.** 42](#_Toc125062172)

[**Supplementary Figure S40. The chromosomal distributions of *Hsf* genes in *A*. *lappa*.** 43](#_Toc125062173)

[**Supplementary Figure S41. The chromosomal distributions of *Hsf* genes in *C*. *canadensis*.** 44](#_Toc125062174)

[**Supplementary Figure S42. The phylogenetic trees and structure analyses of the *Hsf* genes within A1 (A), A2 (B), A3 (C) and A4 (D) subgroups in studied genomes.** 45](#_Toc125062175)

[**Supplementary Figure S43. The phylogenetic trees and structure analyses of the *Hsf* genes within A (A), B (B) and C (C) subgroups in studied genomes.** 46](#_Toc125062176)

[**Supplementary Figure S44. The expression pattern of *Hsf* proteins among *L*. *sativa*.** 47](#_Toc125062177)

**
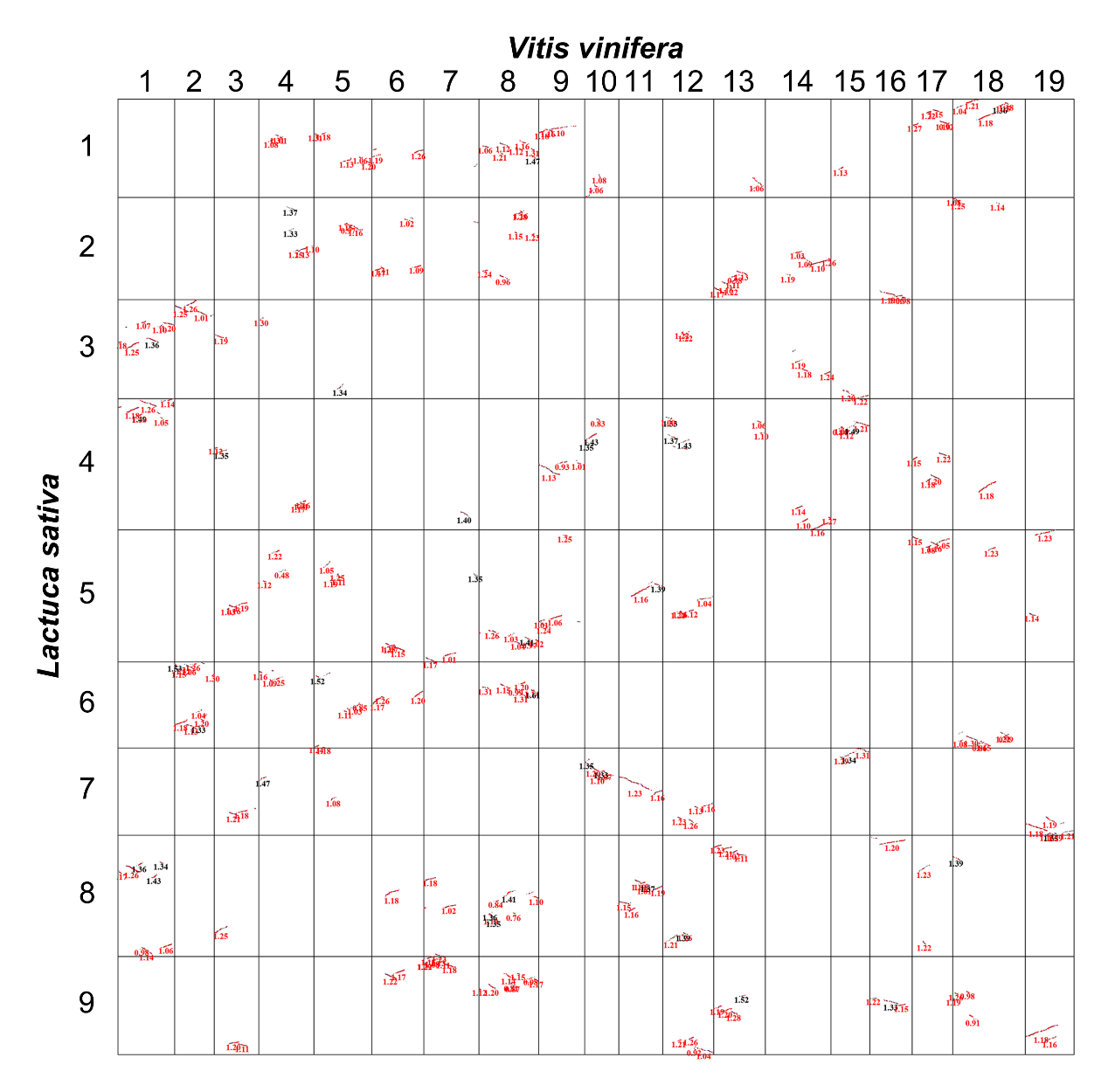
**

**Supplementary** **Figure S1. Intergenomic comparison analyses of the *Vitis* *vinifera* and *Lactuca* *sativa* genomes.** If the anchor gene pairs are the best BLAST hits among the genomes, they are plotted as red dots; otherwise, they are shown in blue dots. Median *Ks* of each inferred syntenic blocks is exhibited near their corresponding regions. The *Ks* values <1.325 were in red, and others are in black.

**
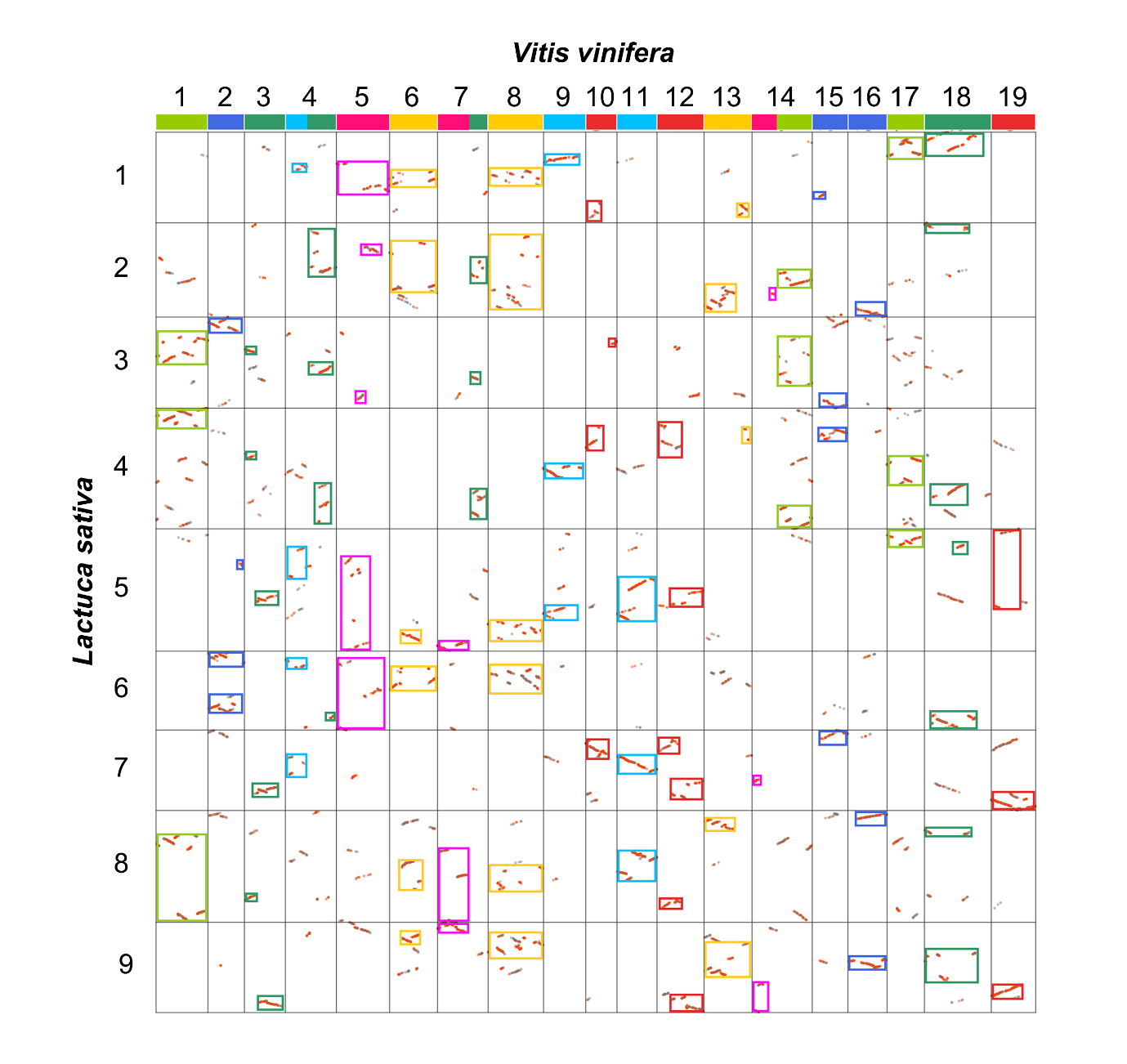
**

**Supplementary** **Figure S2.** **Intergenomic comparative analyses: Syntenic dotplot between the *V*. *vinifera* and *L*. *sativa* genomes.** Genomic syntenic blocks were shown in dotplot according to their genomic locations in *V*. *vinifera* and *L*. *sativa*. The blocks contain at least 10 collinear gene pairs. The collinear gene pairs with the best and secondary BLAST hits were plotted by red and gray dots, respectively. Highlighted box next to the *V*. *vinifera* chromosome corresponds to the seven colors of the eudicot’s ancestor. The part with highlighted color in solid line indicate the selected orthologous regions between *V*. *vinifera* and *L*. *sativa* with syntenic depth ratio of 1:3.


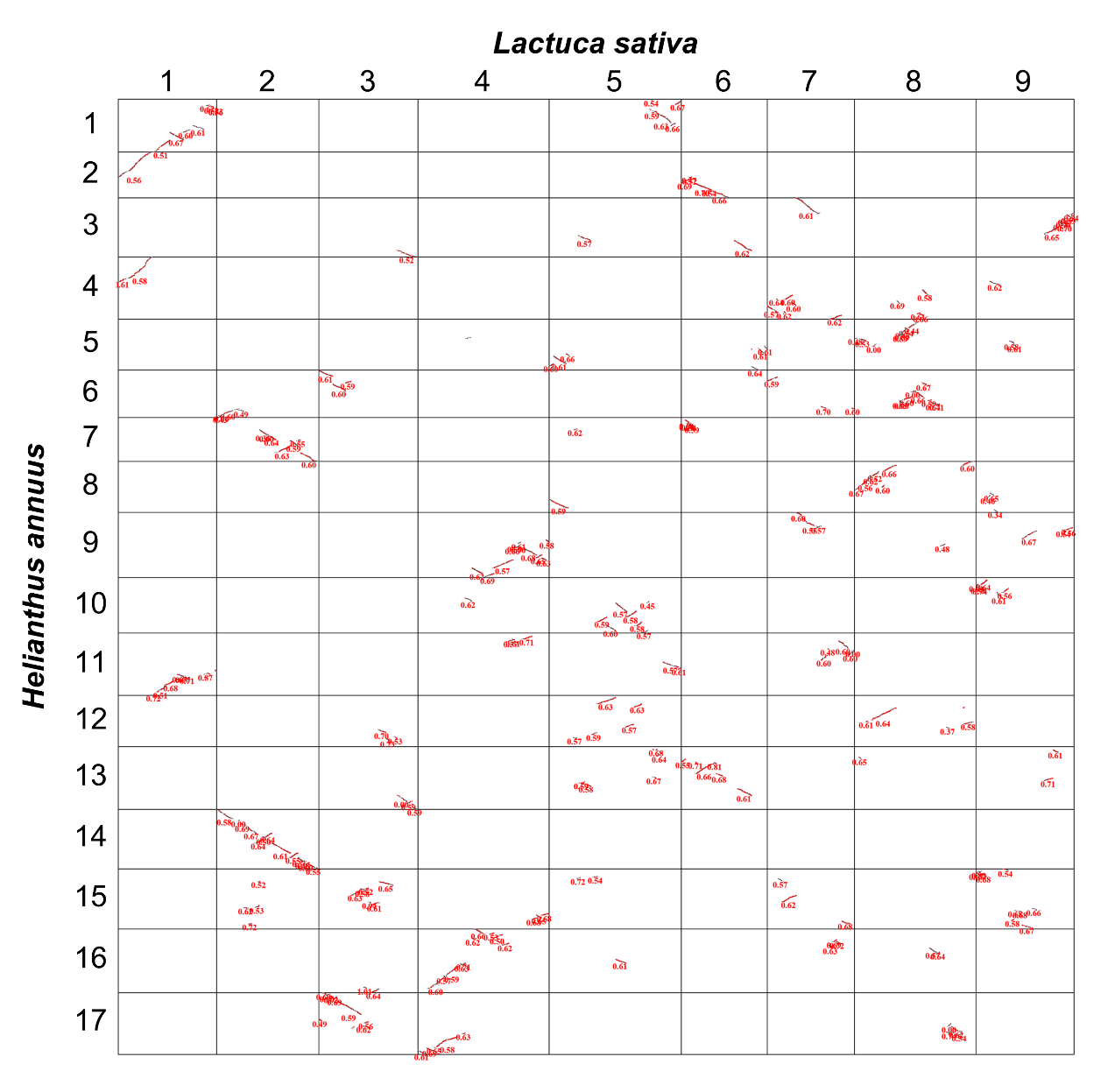


**Supplementary Figure S3.** **Intergenomic comparison analyses of the *L*. *sativa* and *Helianthus* *annuus* genomes.** If the anchor gene pairs are the best BLAST hit among the genomes, they are plotted as red dots; otherwise, they are shown in blue dots. Median *Ks* of each inferred syntenic blocks is exhibited near their corresponding regions. The *Ks* values <1.325 were in red, and others are in black.


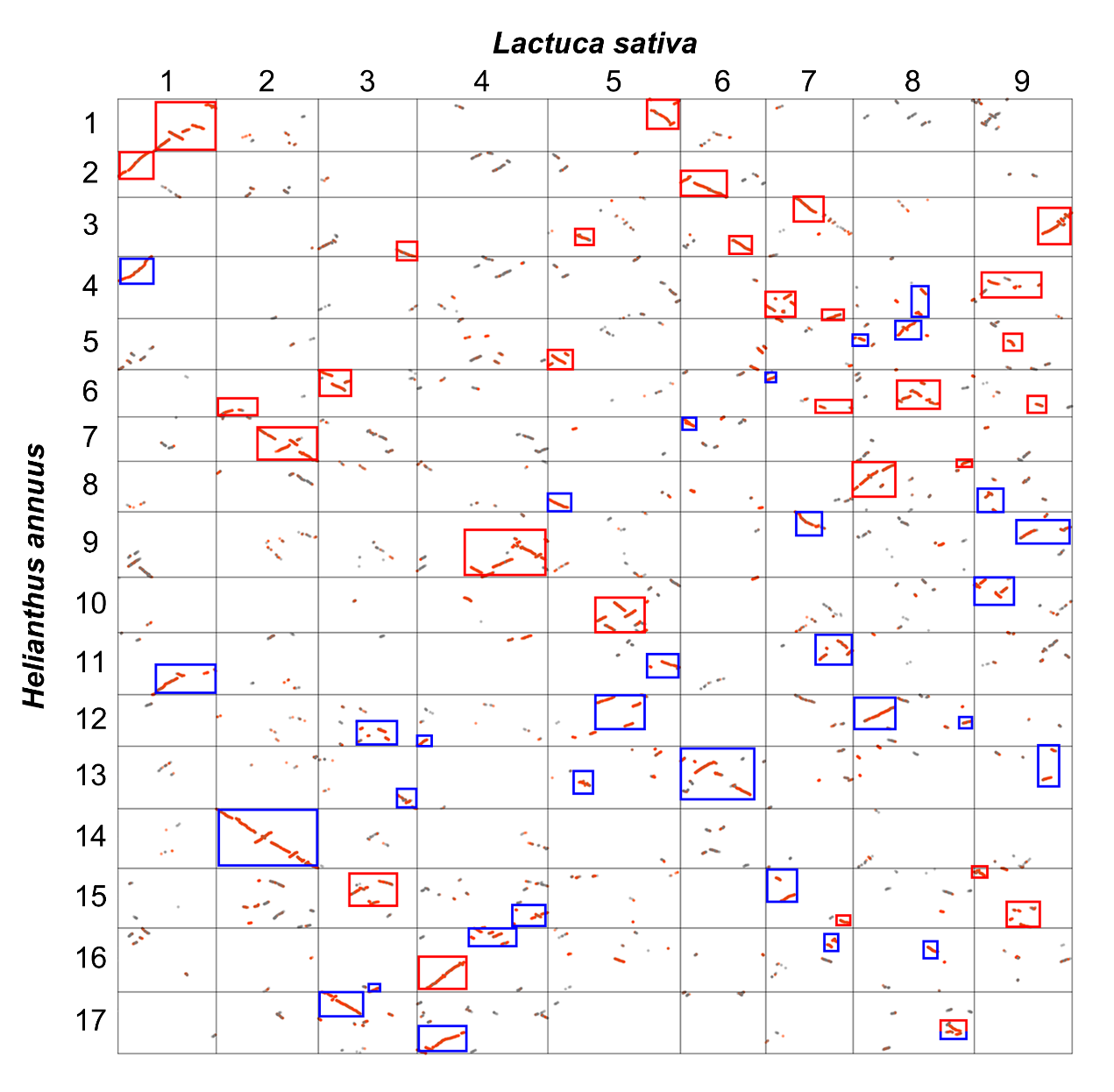


**Supplementary Figure S4. Intergenomic comparative analyses: Syntenic dotplot between the *L*. *sativa* and *H*. *annuus* genomes.** Genomic syntenic blocks were shown in dotplot according to their genomic locations in *L*. *sativa* and *H*. *annuus*. The blocks contain at least 10 collinear gene pairs. The collinear gene pairs with the best and secondary BLAST hits were plotted by red and gray dots, respectively. Highlighted boxes indicate the selected orthologous regions between *L*. *sativa* and *H. annuus* with syntenic depth ratio of 1:2.


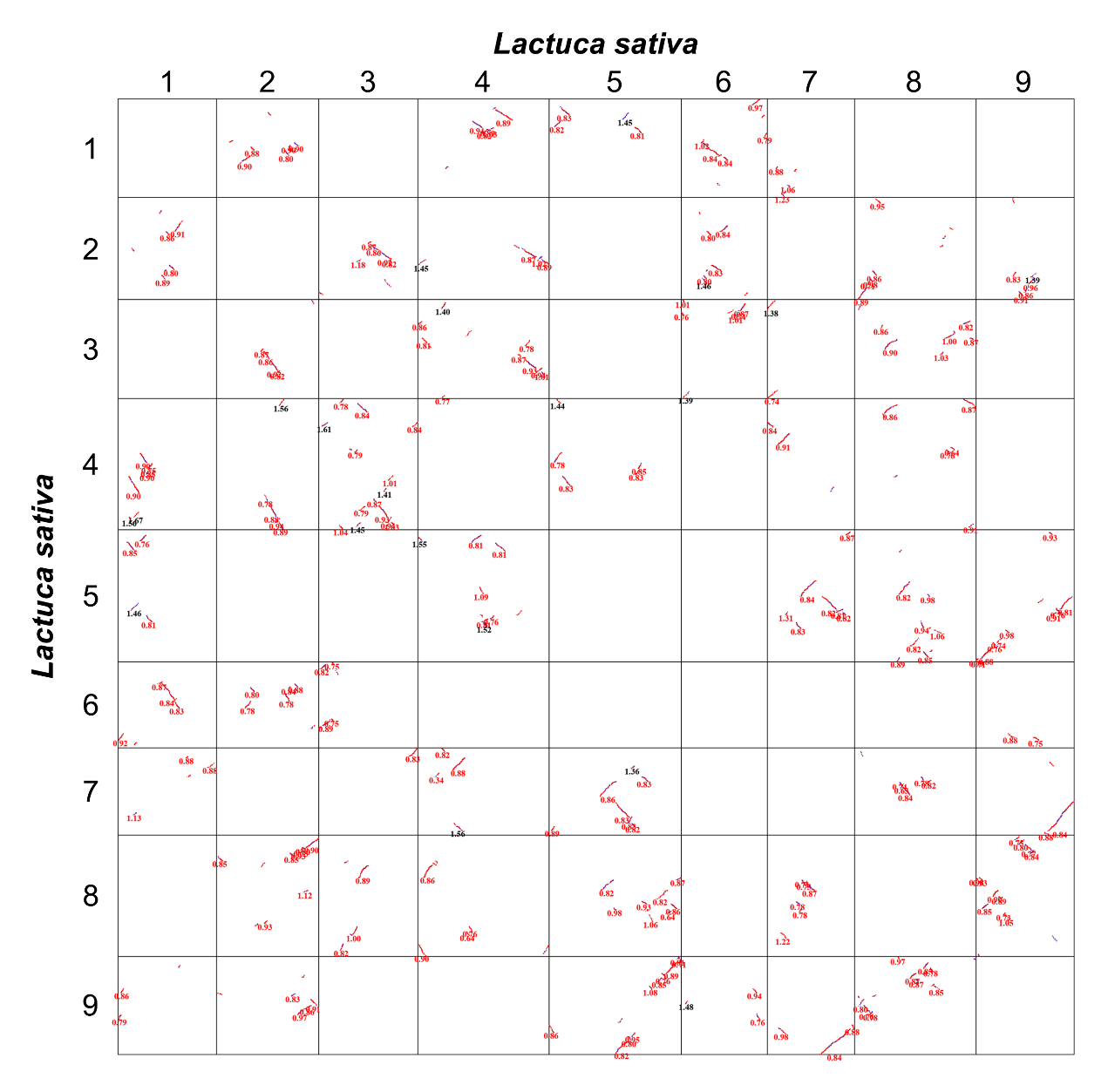


**Supplementary Figure S5. Intragenomic comparison analyses of the *L*. *sativa* genome.** If the anchor gene pairs are the best BLAST hits among the genomes, they are plotted as red dots; otherwise, they are shown in blue dots. Median *Ks* of each inferred syntenic blocks is exhibited near their corresponding regions. The *Ks* values <1.325 were in red, and others are in black.


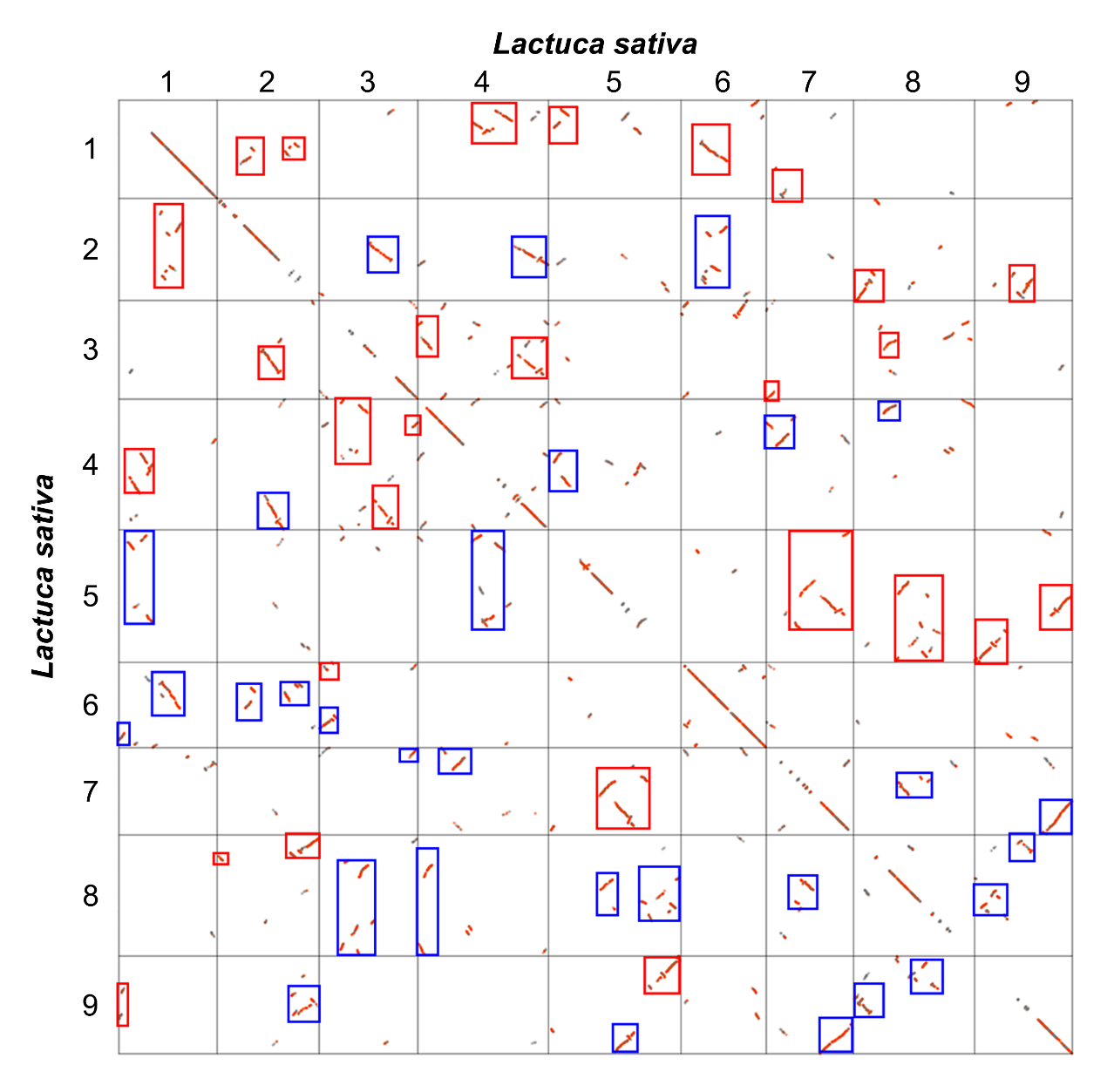


**Supplementary** **Figure S6. Intragenomic comparative analyses: Syntenic dotplot within the *L*. *sativa* genome.** Genomic syntenic blocks were shown in dotplot according to their genomic locations in *L*. *sativa*. The blocks contain at least 10 collinear gene pairs. The collinear gene pairs with the best and secondary BLAST hits were plotted by red and gray dots, respectively. A genomic region of *L*. *sativa* matched two paralogous regions produced by the most recent WGD (Asteraceae common hexaploidization).


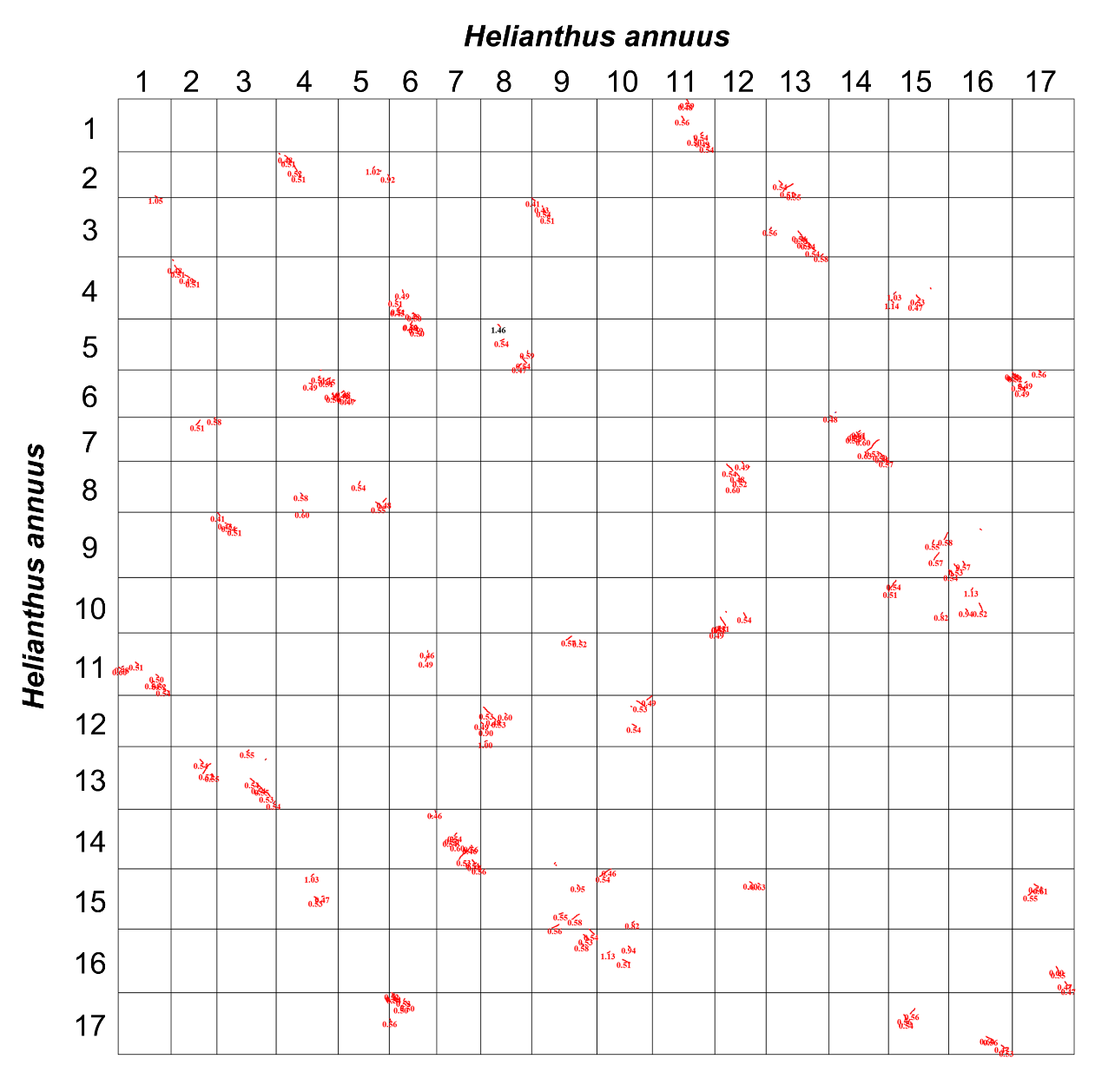


**Supplementary Figure S7. Intragenomic comparison analyses of the *H*. *annuus* genome.** If the anchor gene pairs are the best BLAST hits among the genomes, they are plotted as red dots; otherwise, they are shown in blue dots. Median *Ks* of each inferred syntenic blocks is exhibited near their corresponding regions. The *Ks* values <1.325 were in red, and others are in black.


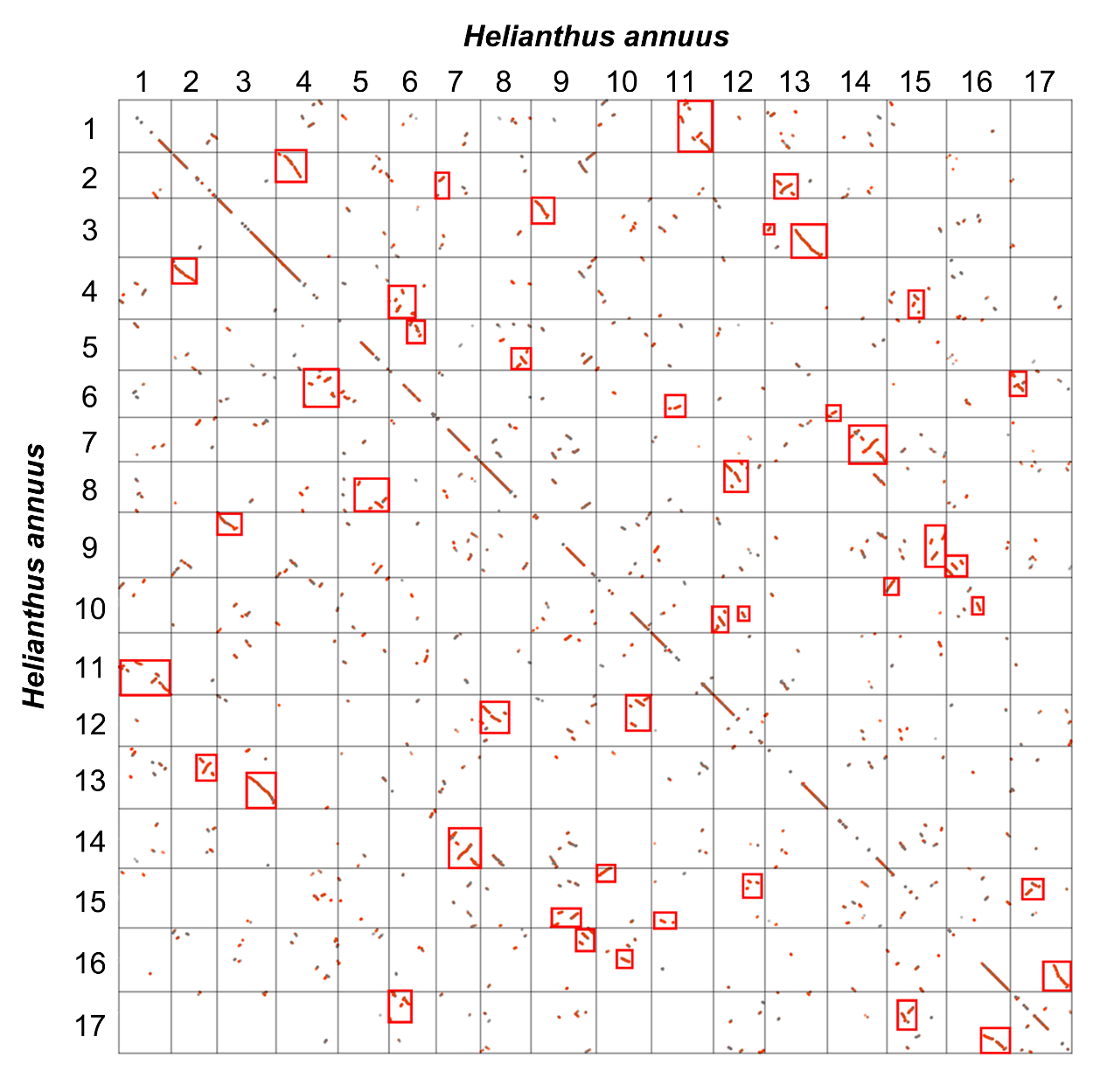


**Supplementary Figure S8. Intragenomic comparative analyses: Syntenic dotplot within the *H*. *annuus* genome.** Genomic syntenic blocks were shown in dotplot according to their genomic locations in *H*. *annuus*. The blocks contain at least 10 collinear gene pairs. The collinear gene pairs with the best and secondary BLAST hits were plotted by red and gray dots, respectively. A genomic region of *H*. *annuus* matched one paralogous region produced by the most recent WGD (Asteroideae specific tetraploidization).

**
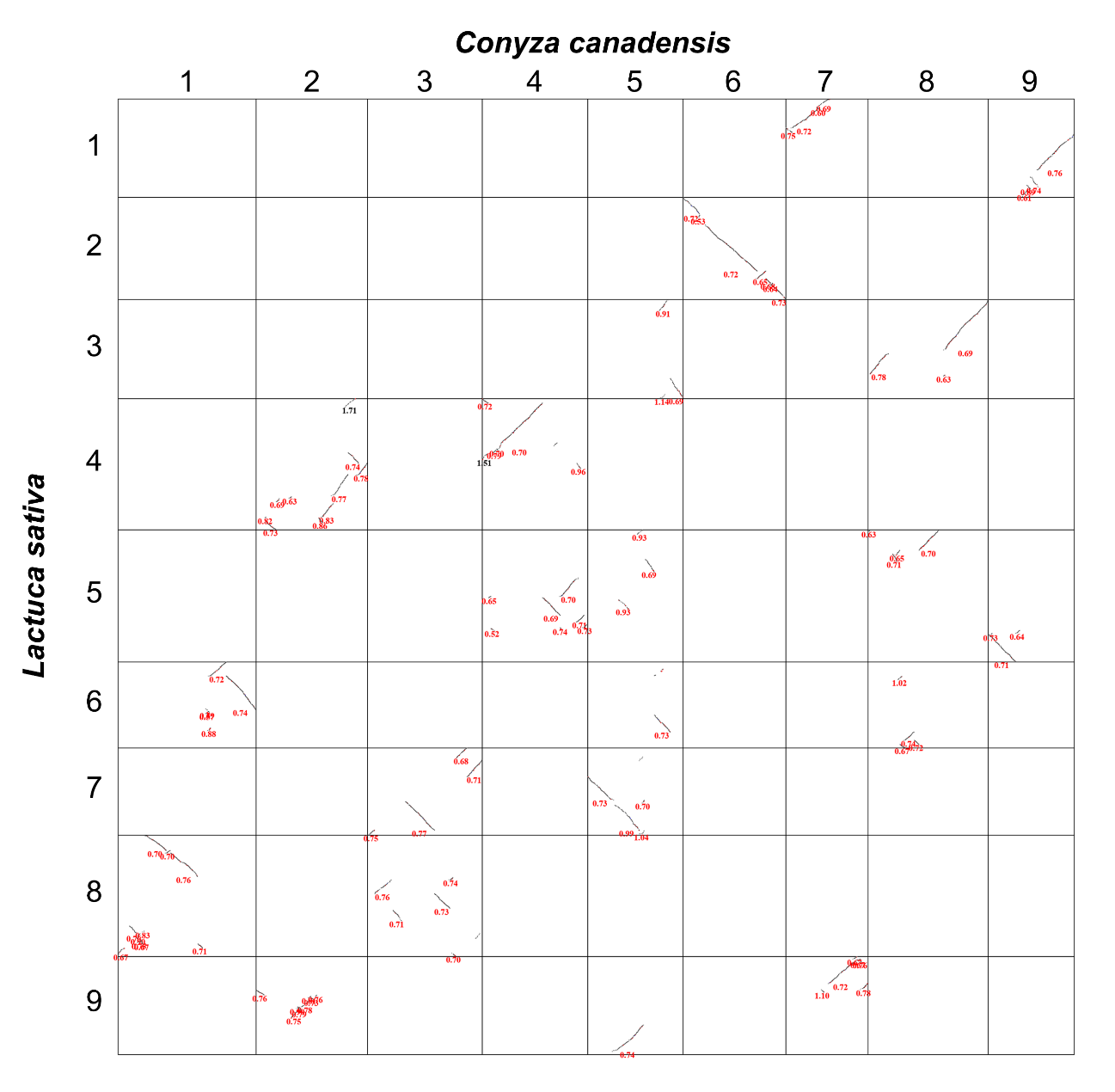
**

**Supplementary Figure S9. Intergenomic comparison analyses of the *L*. *sativa* and *Conyza* *canadensis* genomes.** If the anchor gene pairs are the best BLAST hit among the genomes, they are plotted as red dots; otherwise, they are shown in blue dots. Median *Ks* of each inferred syntenic blocks is exhibited near their corresponding regions. The *Ks* values <1.325 were in red, and others are in black.

**
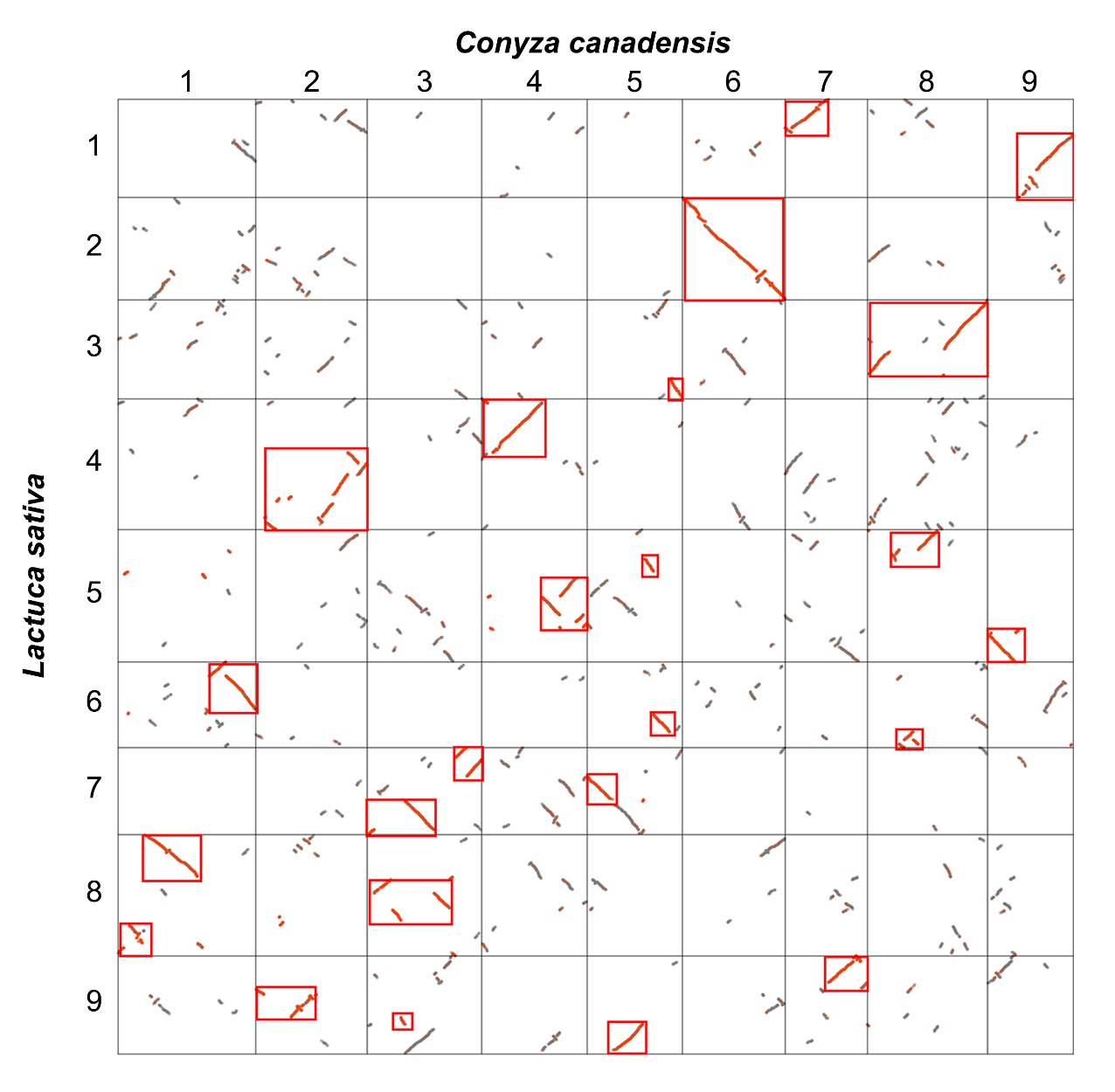
**

**Supplementary** **Figure S10. Intergenomic comparative analyses: Syntenic dotplot between the *L*. *sativa* and *C*. *canadensis* genomes.** Genomic syntenic blocks were shown in dotplot according to their genomic locations in *L*. *sativa* and *C*. *canadensis*. The blocks contain at least 10 collinear gene pairs. The collinear gene pairs with the best and secondary BLAST hits were plotted by red and gray dots, respectively. Highlighted boxes indicate the selected orthologous regions between *L*. *sativa* and *C*. *canadensis* with syntenic depth ratio of 1:1.

**
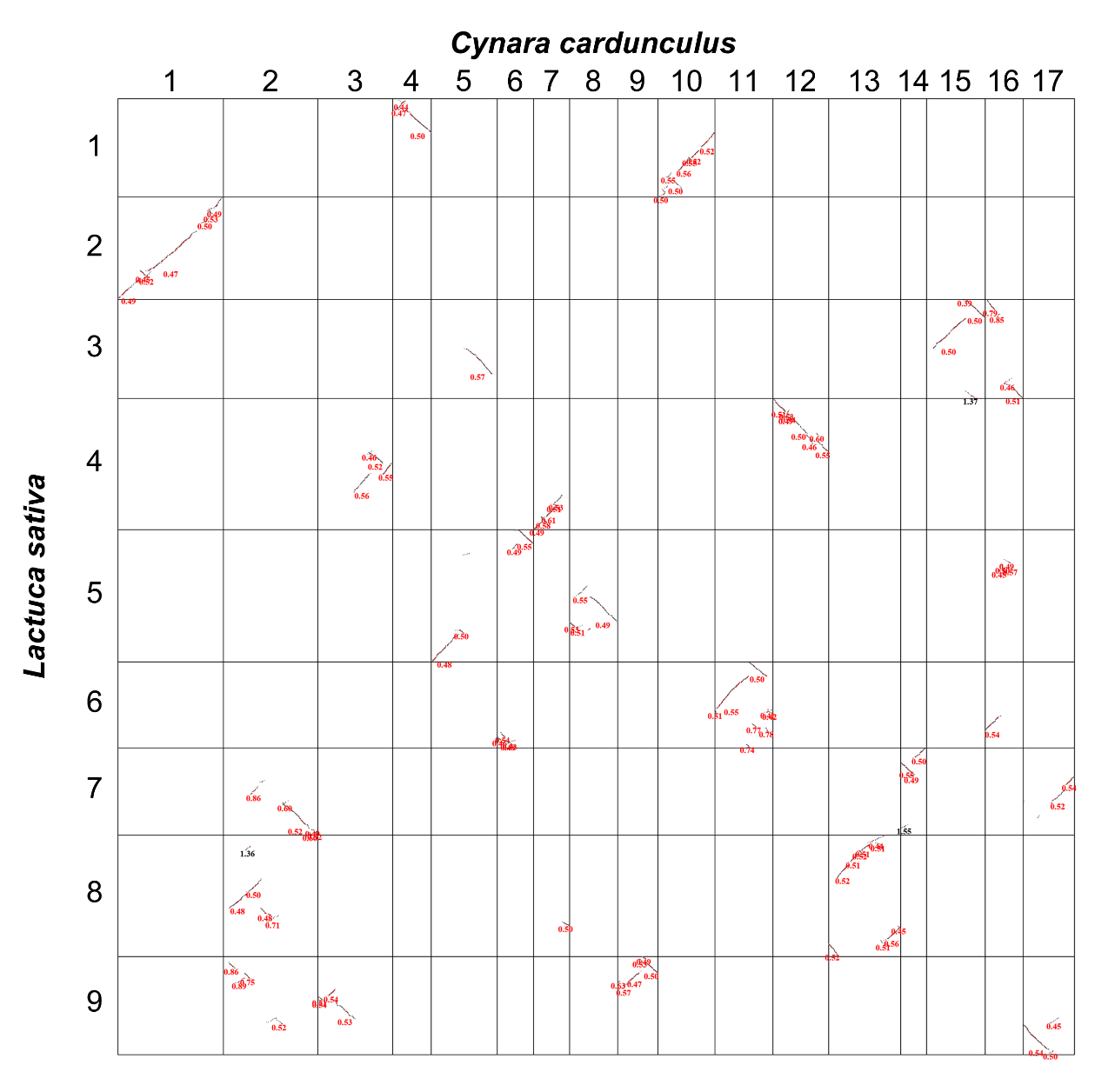
**

**Supplementary Figure S11. Intergenomic comparison analyses of the *L*. *sativa* and *Cynara* *cardunculus* genomes.** If the anchor gene pairs are the best BLAST hit among the genomes, they are plotted as red dots; otherwise, they are shown in blue dots. Median *Ks* of each inferred syntenic blocks is exhibited near their corresponding regions. The *Ks* values <1.325 were in red, and others are in black.

**
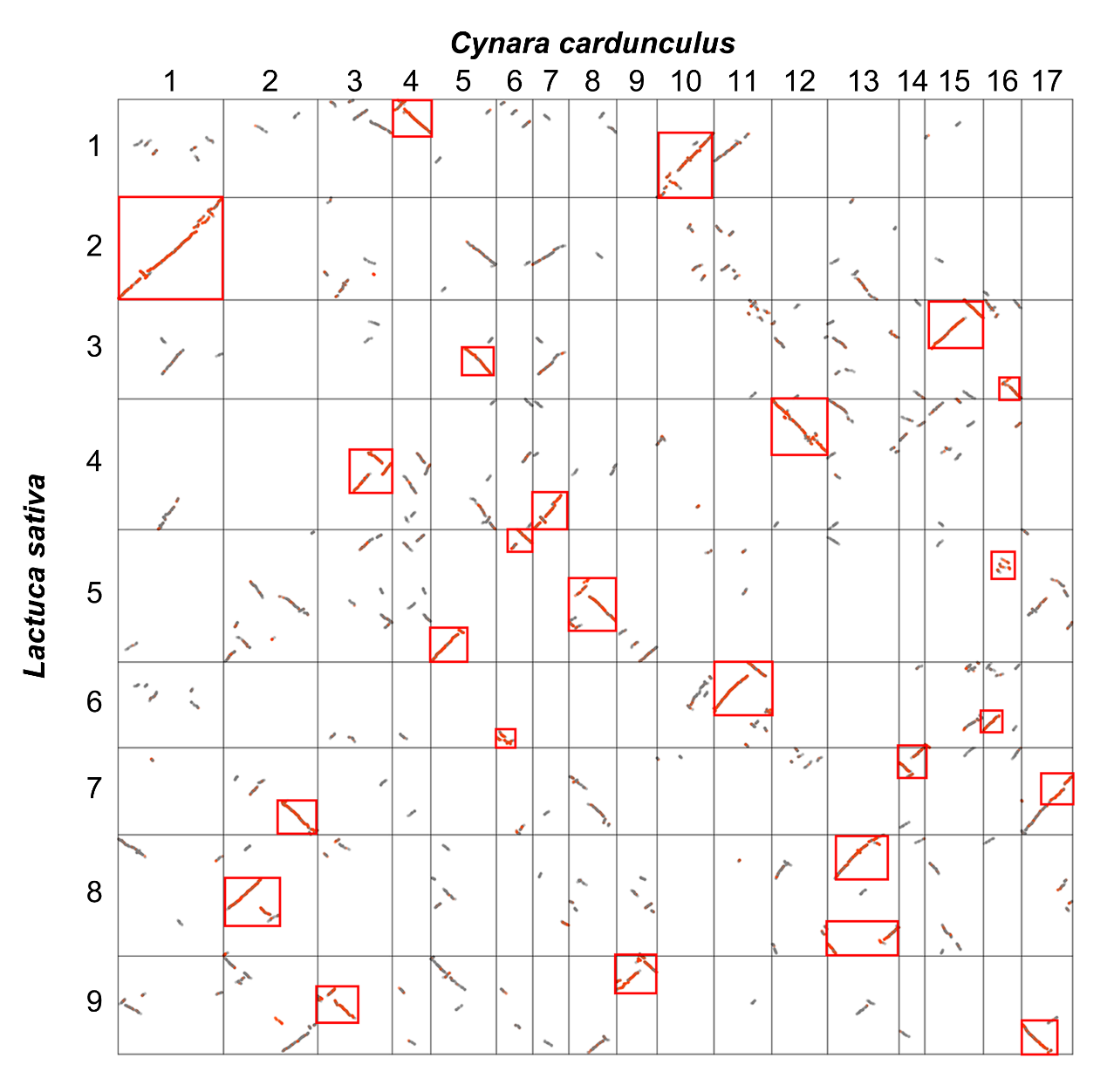
**

**Supplementary Figure S12. Intergenomic comparative analyses: Syntenic dotplot between the *L*. *sativa* and *C*. *cardunculus* genomes.** Genomic syntenic blocks were shown in dotplot according to their genomic locations in *L*. *sativa* and *C*. *cardunculus*. The blocks contain at least 10 collinear gene pairs. The collinear gene pairs with the best and secondary BLAST hits were plotted by red and gray dots, respectively. Highlighted boxes indicate the selected orthologous regions between *L*. *sativa* and *C. cardunculus* with syntenic depth ratio of 1:1.


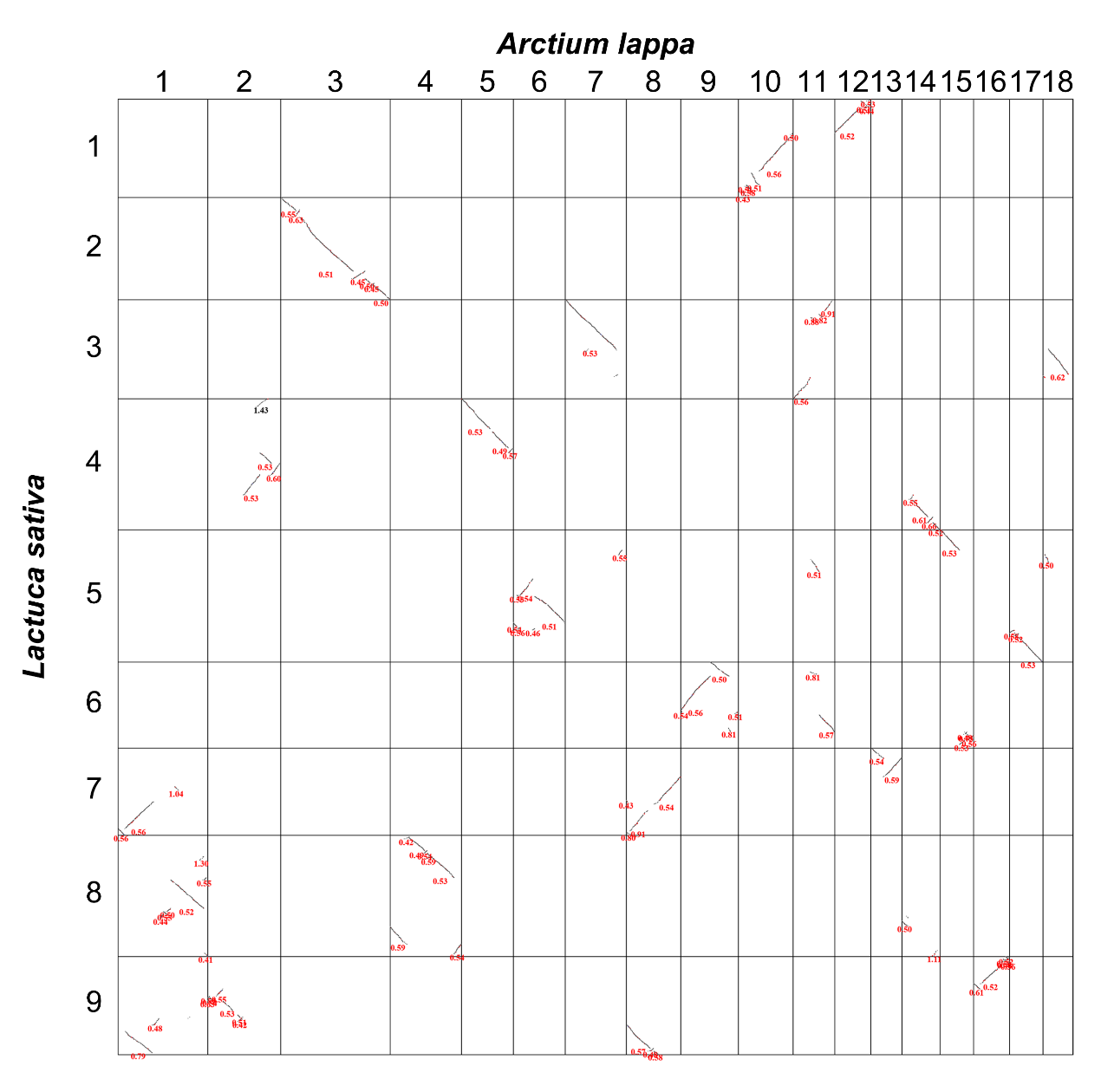


**Supplementary Figure S13. Intergenomic comparison analyses of the *L*. *sativa* and *Arctium* *lappa*.** If the anchor gene pairs are the best BLAST hit among the genomes, they are plotted as red dots; otherwise, they are shown in blue dots. Median *Ks* of each inferred syntenic blocks is exhibited near their corresponding regions. The *Ks* values <1.325 were in red, and others are in black.


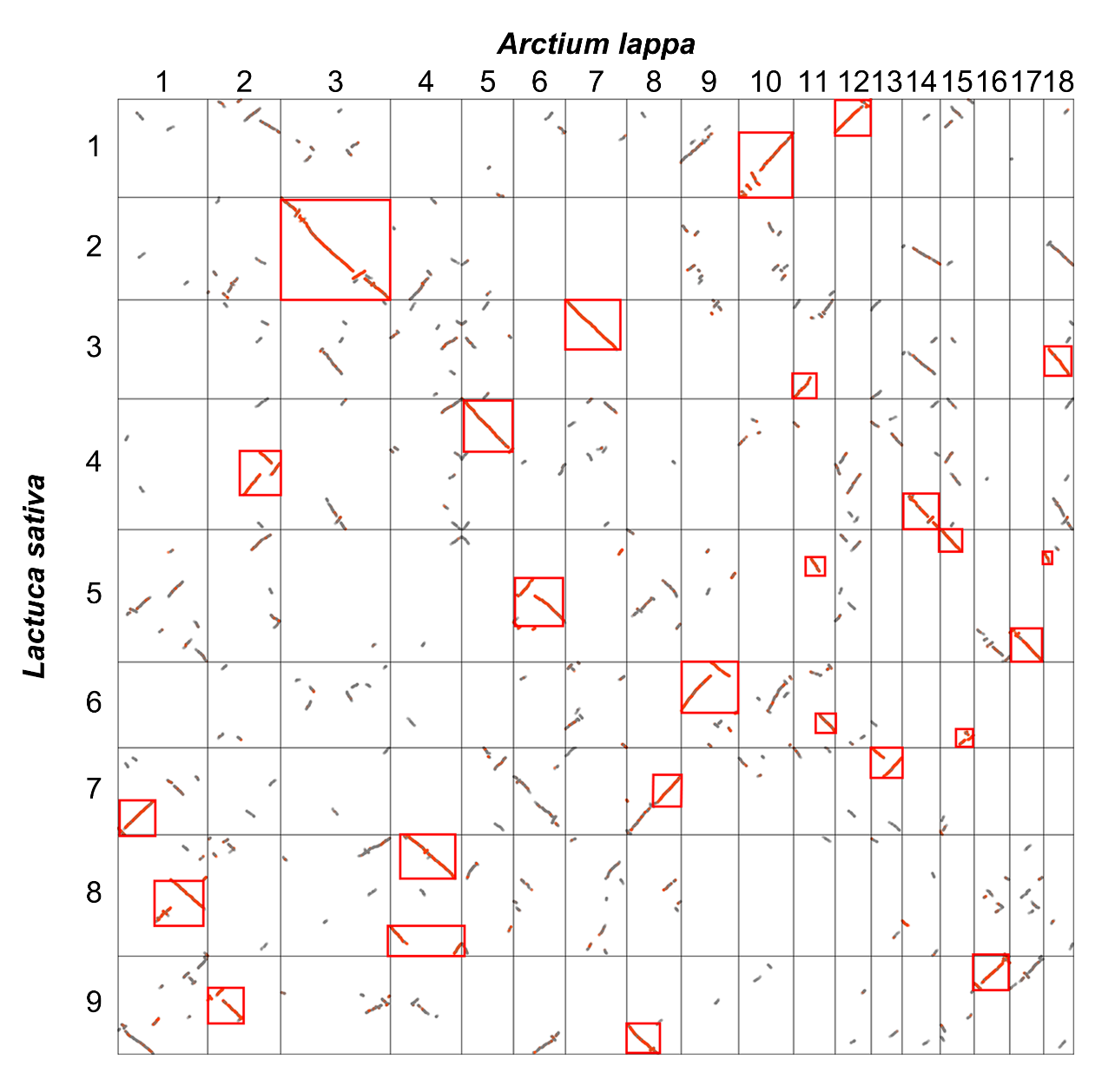


**Supplementary Figure S14. Intergenomic comparative analyses: Syntenic dotplot between the *L*. *sativa* and *A*. *lappa* genomes.** Genomic syntenic blocks were shown in dotplot according to their genomic locations in *L*. *sativa* and *A*. *lappa*. The blocks contain at least 10 collinear gene pairs. The collinear gene pairs with the best and secondary BLAST hits were plotted by red and gray dots, respectively. Highlighted boxes indicate the selected orthologous regions between *L*. *sativa* and *A*. *lappa* with syntenic depth ratio of 1:1.


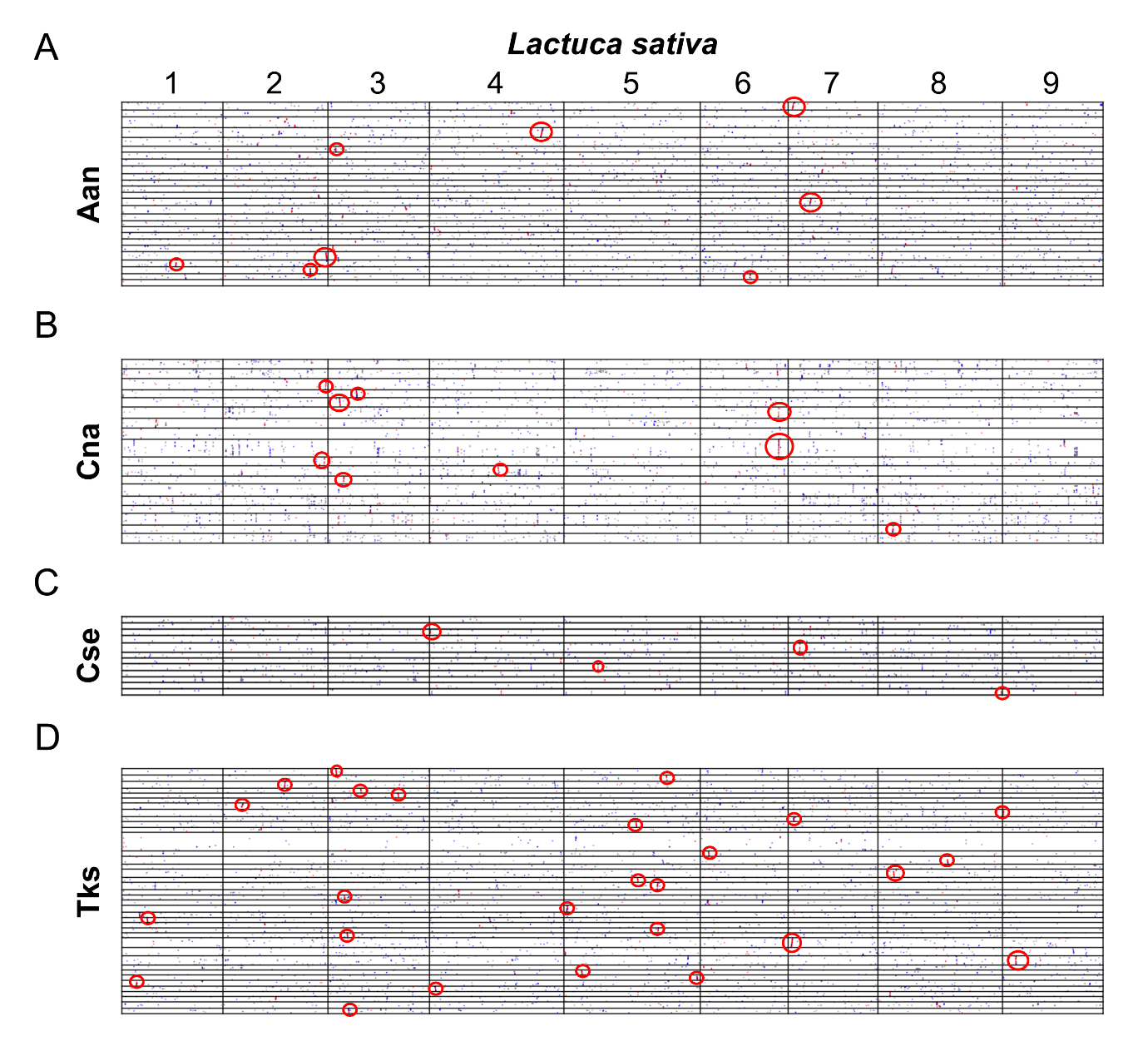


**Supplementary Figure S15. Intergenomic homologous structure between the genomes of *L*. *sativa* and other Asteraceae (*Artemisia* *annua*, *Chrysanthemum* *nankingense*, *Chrysanthemum* *seticuspe*, and *Taraxacum* *kok-saghyz*).** The best, secondary, and other matched homologous gene pairs output by Blastp were plotted by red, blue, and gray colors in this figure, respectively. Highlighted boxes indicate the selected orthologous regions between *L*. *sativa* and other Asteraceae scaffold with syntenic depth ratio of 1:1. (A) *L. sativa* vs. *A*. *annua* (Aan) scaffold; (B) *L*. *sativa* vs. *C*. *nankingense* (Cna) scaffold; (C) *L*. *sativa* vs. *C*. *seticuspe* (Cse) scaffold; (D) *L*. *sativa* vs. *T*. *kok-saghyz* (Tko) scaffold.


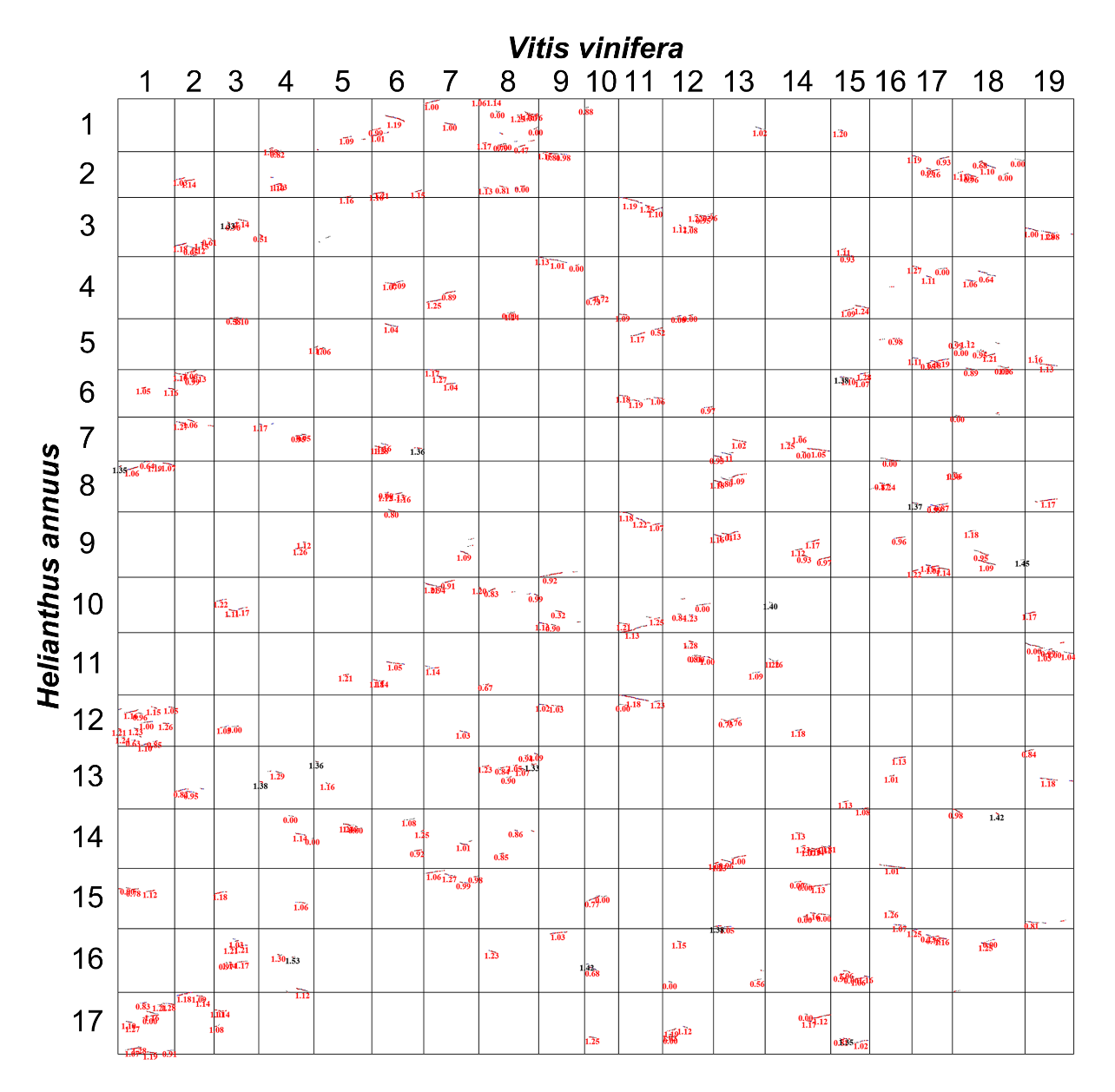


**Supplementary Figure S16. Intergenomic comparison analyses of the *V*. *vinifera* and *H*. *annuus* genomes.** If the anchor gene pairs are the best BLAST hits among the genomes, they are plotted as red dots; otherwise, they are shown in blue dots. Median *Ks* of each inferred syntenic blocks is exhibited near their corresponding regions. The *Ks* values <1.325 were in red, and others are in black.


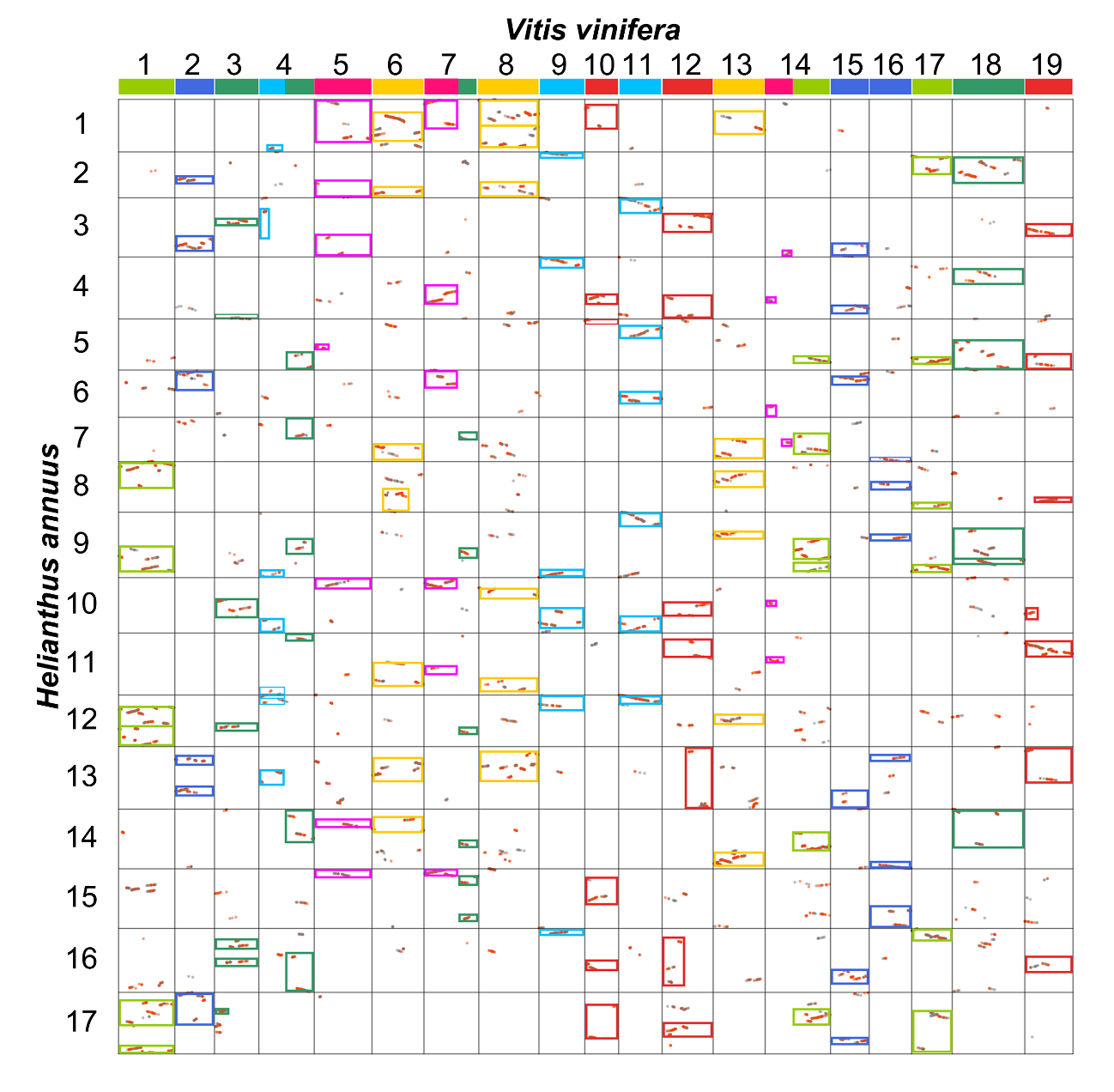


**Supplementary Figure S17. Intergenomic comparative analyses: Syntenic dotplot between the *V*. *vinifera* and *H*. *annuus* genomes.** Genomic syntenic blocks were shown in dotplot according to their genomic locations in *V*. *vinifera* and *H*. *annuus*. The blocks contain at least 10 collinear gene pairs. The collinear gene pairs with the best and secondary BLAST hits were plotted by red and gray dots, respectively. Highlighted box next to the *V*. *vinifera* chromosome corresponds to the seven colors of the eudicot’s ancestor. The part with highlighted color in solid line indicate the selected orthologous regions between *V*. *vinifera* and *H*. *annuus* with syntenic depth ratio of 1:6.

**
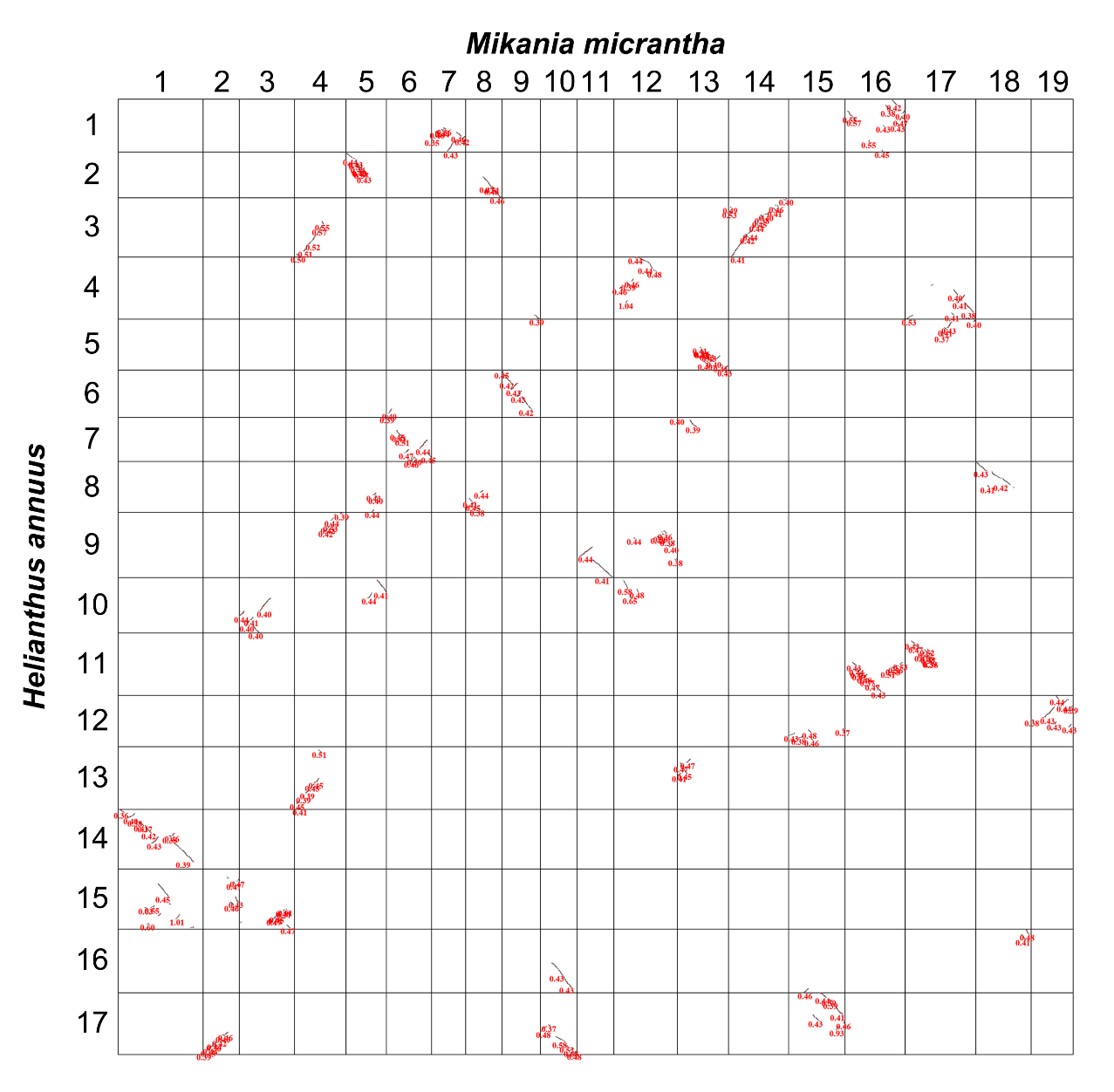
**

**Supplementary Figure S18. Intergenomic comparison analyses of the *H*. *annuus* and *Mikania* *micrantha* genomes.** If the anchor gene pairs are the best BLAST hits among the genomes, they are plotted as red dots; otherwise, they are shown in blue dots. Median *Ks* of each inferred syntenic blocks is exhibited near their corresponding regions. The *Ks* values <1.325 were in red, and others are in black.


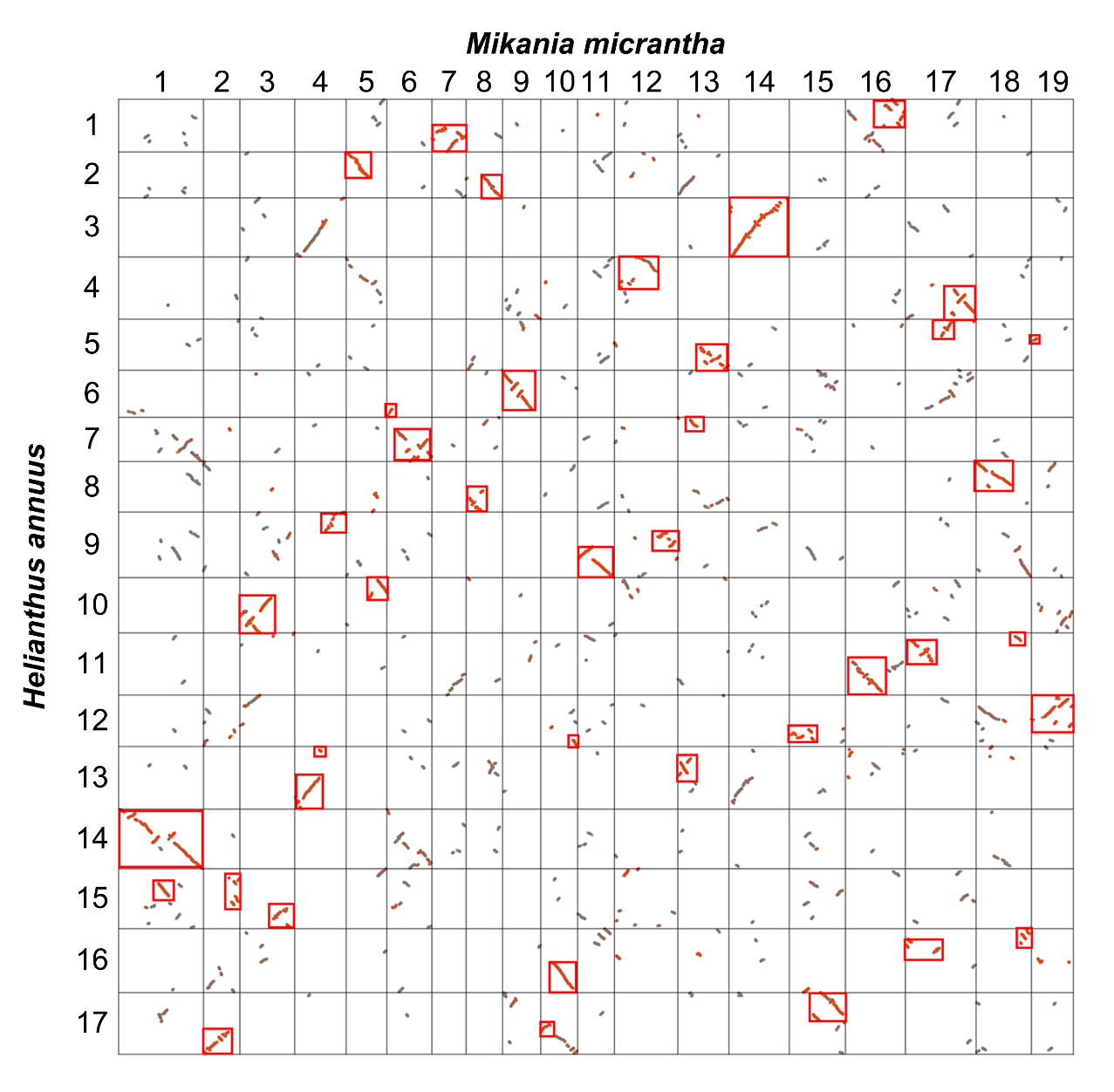


**Supplementary Figure S19. Intergenomic comparative analyses: Syntenic dotplot between the *H*. *annuus* and *M*. *micrantha* genomes.** Genomic syntenic blocks were shown in dotplot according to their genomic locations in *H*. *annuus* and *M*. *micrantha*. The blocks contain at least 10 collinear gene pairs. The collinear gene pairs with the best and secondary BLAST hits were plotted by red and gray dots, respectively. Highlighted boxes indicate the selected orthologous regions between *H*. *annuus* and *M*. *micrantha* with syntenic depth ratio of 1:1.


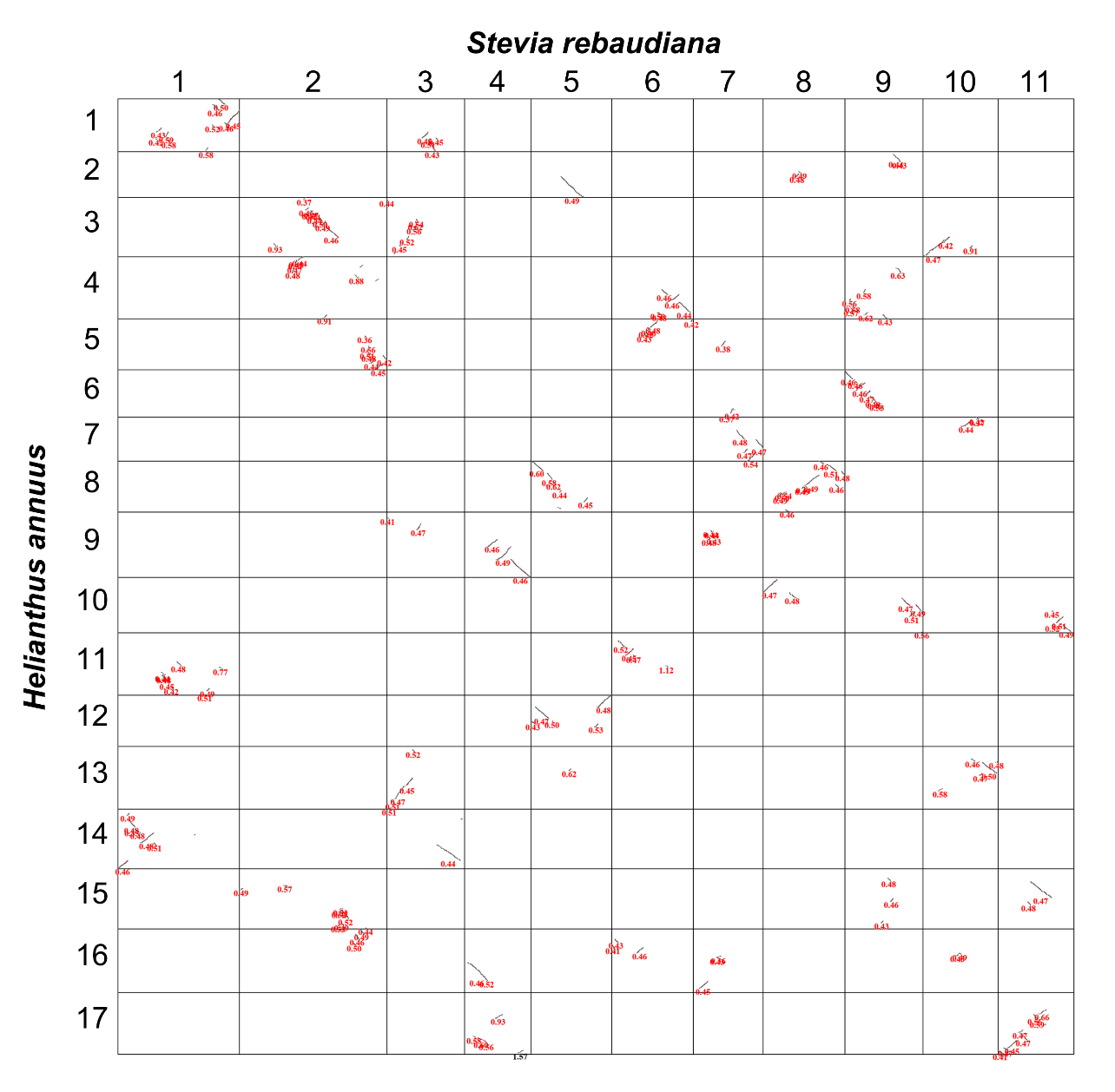


**Supplementary Figure S20. Intergenomic comparison analyses of the *H*. *annuus* and *Stevia* *rebaudiana* genomes.** If the anchor gene pairs are the best BLAST hits among the genomes, they are plotted as red dots; otherwise, they are shown in blue dots. Median *Ks* of each inferred syntenic blocks is exhibited near their corresponding regions. The *Ks* values <1.325 were in red, and others are in black.


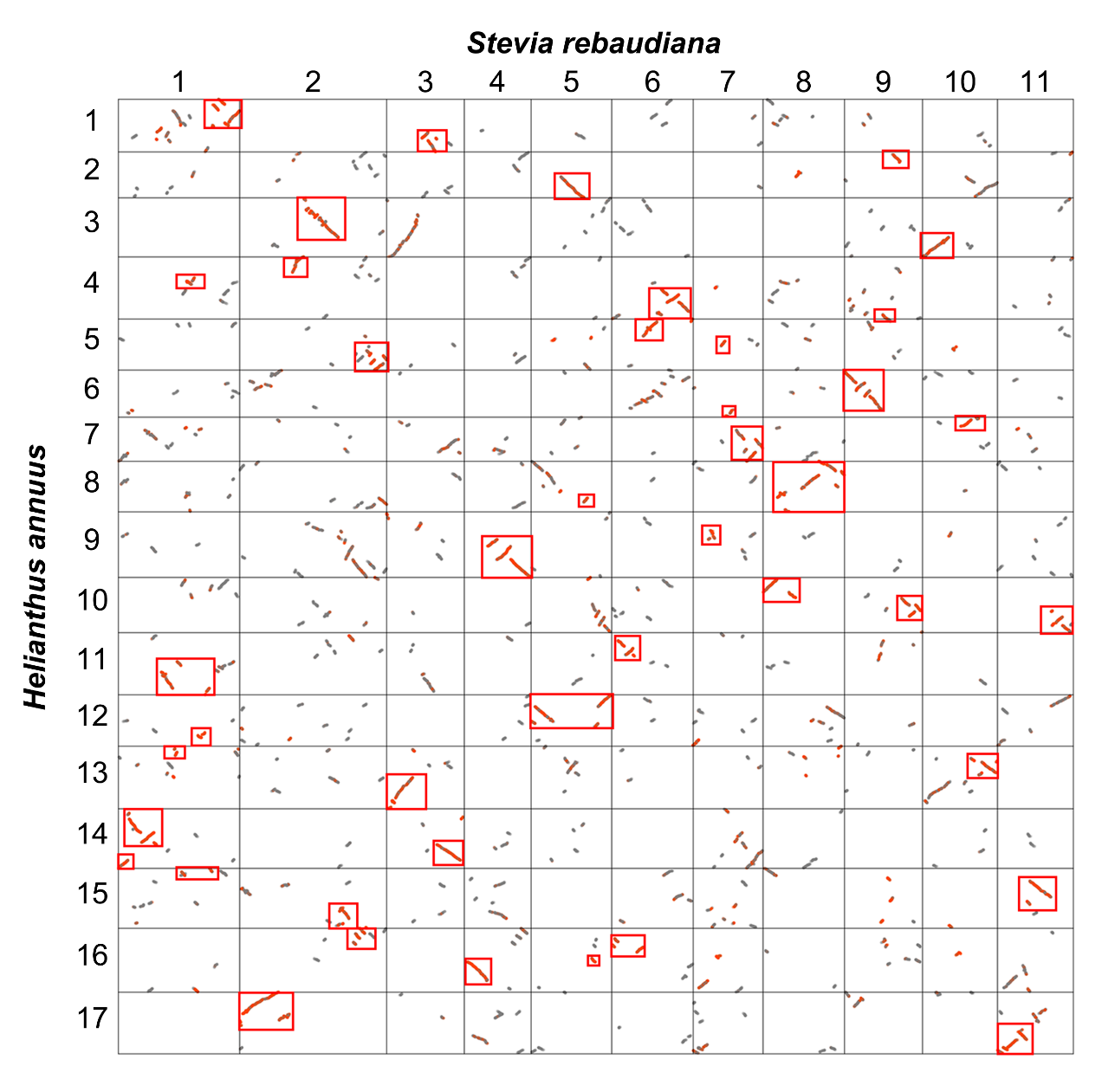


**Supplementary Figure S21. Intergenomic comparative analyses: Syntenic dotplot between the *H*. *annuus* and *S*. *rebaudiana* genomes.** Genomic syntenic blocks were shown in dotplot according to their genomic locations in *H*. *annuus* and *S*. *rebaudiana*. The blocks contain at least 10 collinear gene pairs. The collinear gene pairs with the best and secondary BLAST hits were plotted by red and gray dots, respectively. The blocks contain at least 10 collinear gene pairs are representing in dotplot. Highlighted boxes indicate the selected orthologous regions between *H*. *annuus* and *S*. *rebaudiana* with syntenic depth ratio of 1:1.


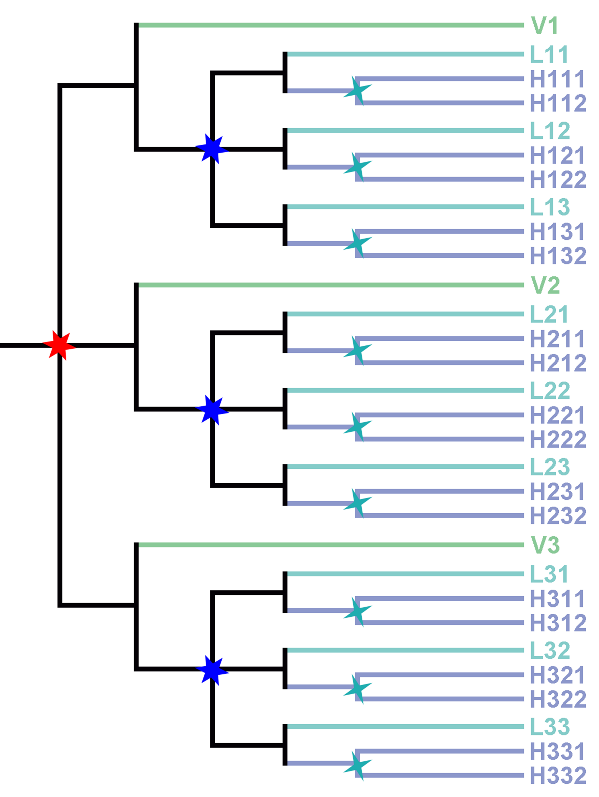


**Supplementary Figure S22. Gene phylogenetic tree of** ***V*. *vinifera*,** ***L*. *sativa*, and** ***H*. *annuus*.** The star labels on the trees indicate the inferred polyploidizations among three considered species, in which the core eudicot common hexaploidization (ECH) color-coded by red hexagonal star label, ACH event color-coded by blue hexagonal star label, AST event color-coded by cyan four-pointed star label. Three ECH-produced paralogous genes in *V*. *vinifera* (V) are denoted V1, V2, and V3, each having three orthologous genes in the *L*. *sativa* (L) genome and six in the *H*. *annuus* (H) genome


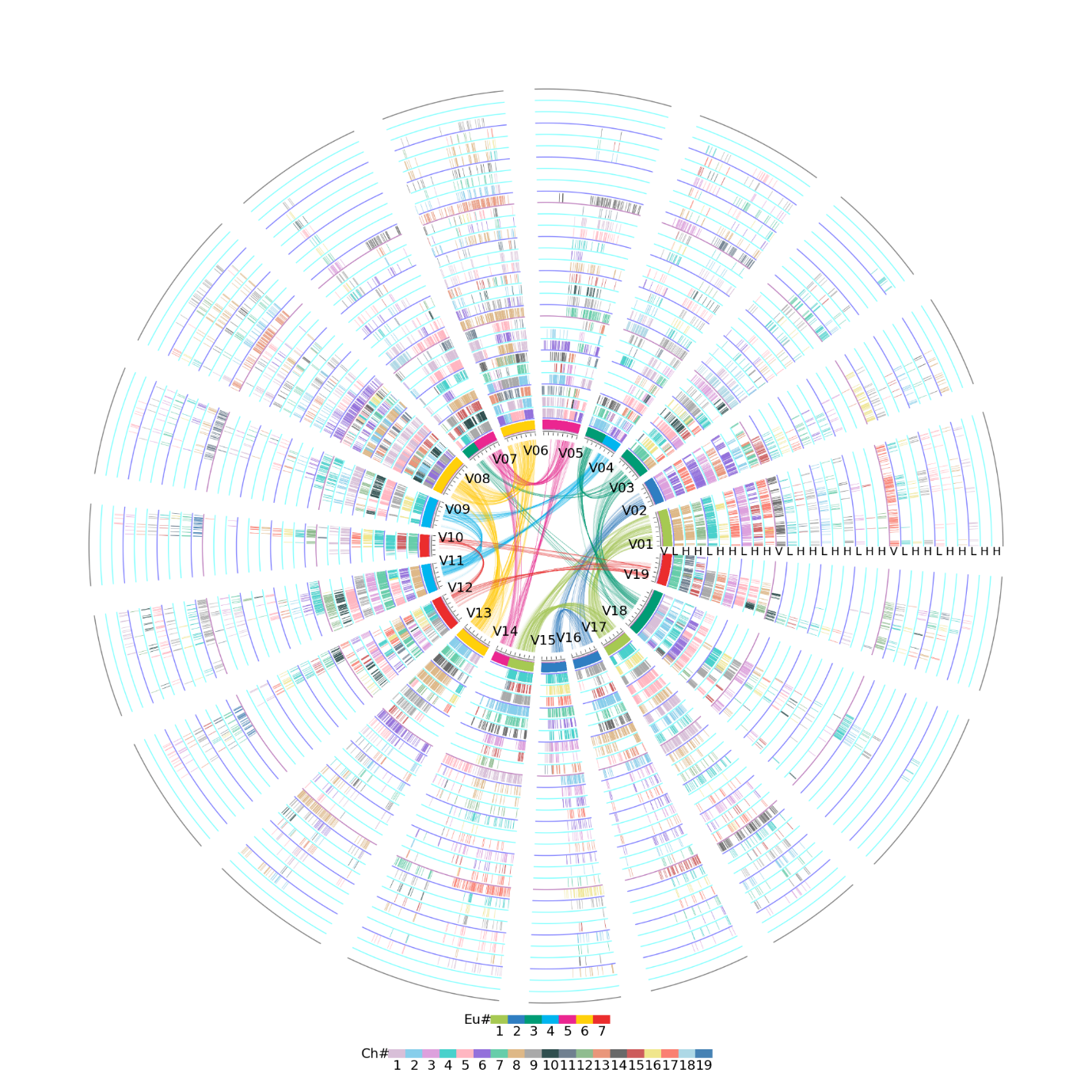


**Supplementary Figure S23. Genomic alignment of *L*. *sativa* and *H*. *annuus*.** The chromosomes of *V*. *vinifera* constitute the innermost circle, and their syntenic paralogous genes are linked by curved lines and correspondingly coloured according to the previously inferred 7 core-eudicot-common ancestor chromosomes. *L*. *sativa* is denoted by L, *H*. *annuus* is denoted by H, and *V*. *vinifera* is denoted by V. Each chromosomal region had 3 and 6 orthologous regions in the *L*. *sativa* and *H*. *annuus* genomes, respectively; these regions are displayed as 30 circles, where each circle represents one subgenome. Short lines between two circles show syntenic genes, which correspond to the colours of the encoded Ch#. The colour scheme is shown at the bottom.


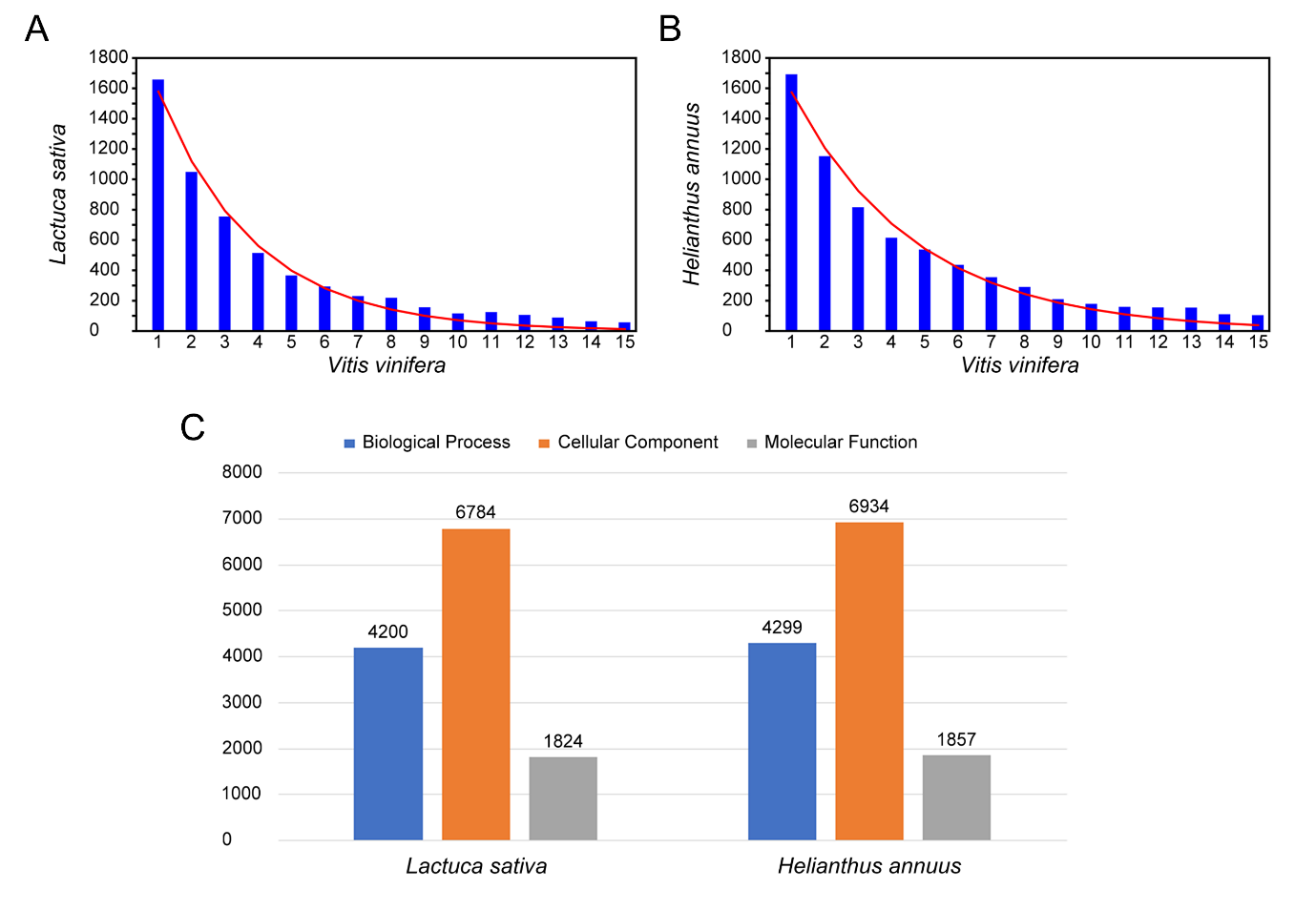


**Supplementary Figure S24. Gene loss analysis of *L*. *sativa* and *H*. *annuus* genomes.** (A-B) Gene loss rates of the *L*. *sativa* and *H*. *annuus*. genome. The x-axis represents the number of continuously lost genes in the region of gene collinearity, the y-axis represents the number of losses, and the red curve is the model fit of gene loss. (C) Functional genes lost during the evolution of *L*. *sativa* and *H*. *annuus*. The C figure shows the functions of 7,779 and 7,950 lost functional genes found in *L*. *sativa* and *H*. *annuus*, respectively. Most of the lost genes were components of cells (*L*. *sativa* 87.4%, *H*. *annuus* 87.3%), more than half were related to biological processes (*L*. *sativa* 54.1%, *H*. *annuus* 54.1%) and a few were associated with molecular functions (*L*. *sativa* 23.5%, *H*. *annuus* 23.4%). The x-axis shows the different Asteraceae species. The y-axis shows the number of functional genes identified.


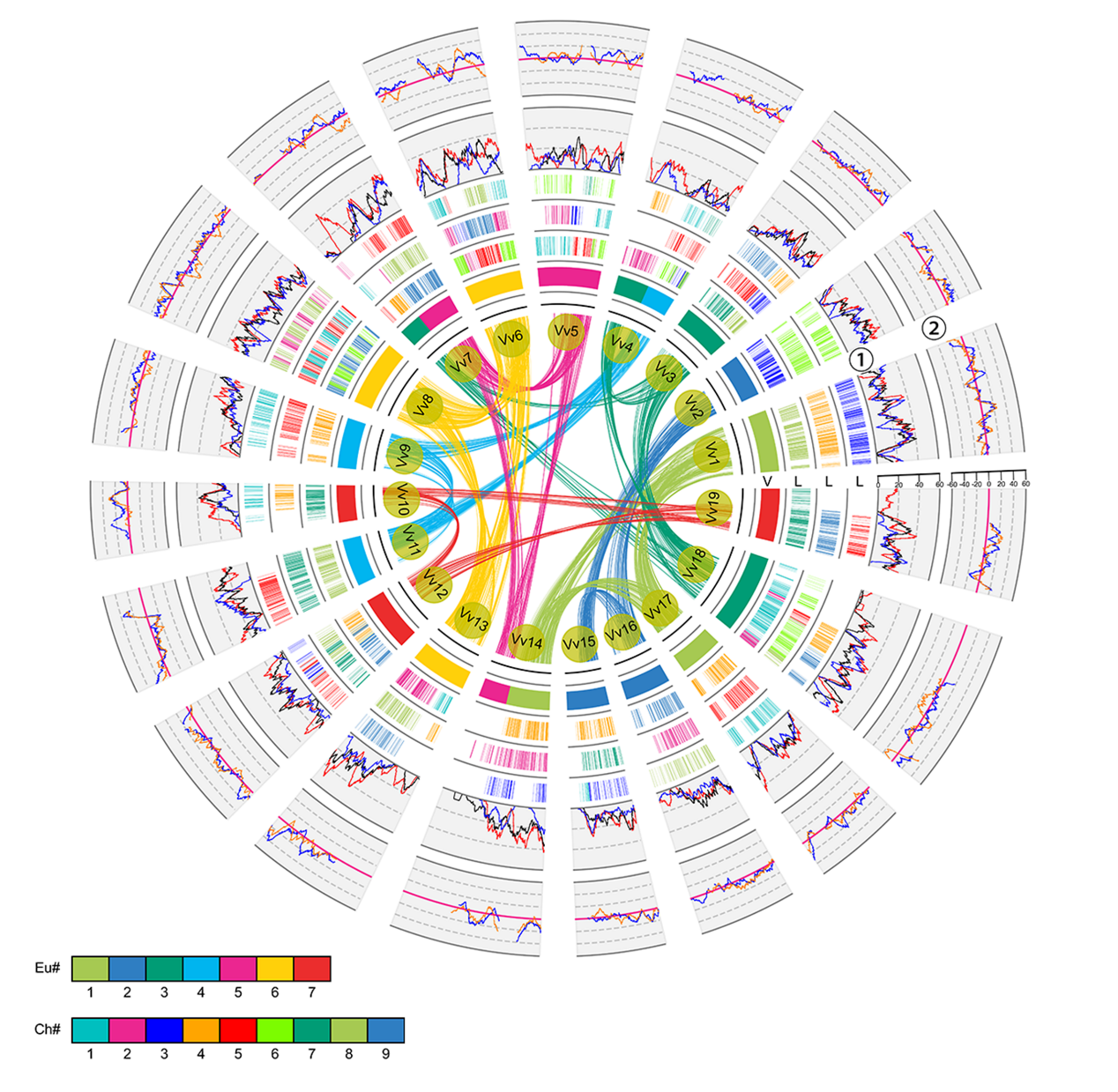


**Supplementary Figure S25. ACH event in *L*. *sativa* generates intersubgenome retention level balance.** Genomic alignments and gene retention of *L*. *sativa* subgenomes along corresponding orthologous *V*. *vinifera* chromosomes. The genes in 19 chromosomes of *V*. *vinifera* within the inner circle colored by the 7 core-eudicot-common ancestor chromosomes, as shown in the color scheme at the bottom (denoted by Eu#). Compared to the *V*. *vinifera* genome, the genomic paralogous and orthologous information within and among the genome of *L*. *sativa* in subgenomes is displayed in three circles. Each circle is formed by short vertical lines that denote homologous orthologous genes, which are colored to indicate the 9 *L*. *sativa* chromosome numbers in their respective source plant, as shown in the color scheme at the bottom (denoted by Ch#). In ①, gene retention level of homologous region group 1 (red), 2 (blue), and 3 (black) in *L*. *sativa*, with *V*. *vinifera* as the reference; in ②, difference in gene retention between homologous groups 1 and 2 (blue) and between homologous groups 2 and 3 (orange).


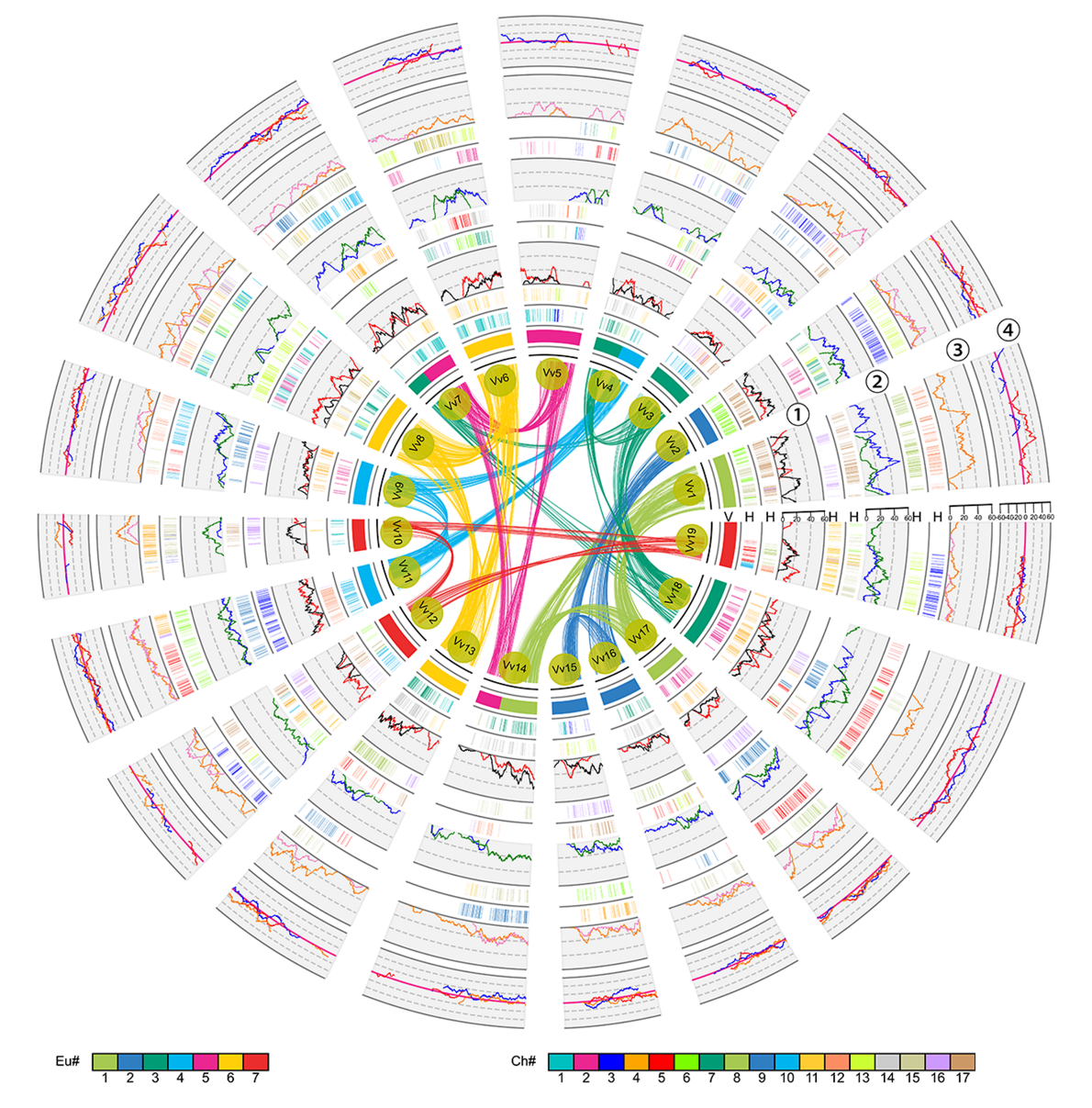


**Supplementary Figure S26. Polyploidization event in *H*. annuus generates intersubgenome retention level balance.** Genomic alignments and gene retention of *H*. *annuus* subgenomes along corresponding orthologous *V*. *vinifera* chromosomes. The genes in 19 chromosomes of *V*. *vinifera* within the inner circle colored by the 7 core-eudicot-common ancestor chromosomes, as shown in the color scheme at the bottom (denoted by Eu#). Compared to the *V*. *vinifera* genome, the genomic paralogous and orthologous information within and among the genome of *H*. *annuus* in subgenomes is displayed in six circles. Each circle is formed by short vertical lines that denote homologous orthologous genes, which are colored to indicate the 17 *H*. *annuus* chromosome numbers in their respective source plant, as shown in the color scheme at the bottom (denoted by Ch#). In ①, gene retention level of homologous region group 1 (red) and 2 (black) in *H*. *annuus*, with *V*. *vinifera* as the reference; in ②, gene retention level of homologous region group 3 (blue) and 4 (green) in *H*. *annuus*; in ③, gene retention level of homologous region group 5 (pink) and 6 (orange) in *H*. *annuus*; in ④, difference in gene retention between homologous groups 1 and 2 (blue), between homologous groups 3 and 4 (red) and between homologous groups 5 and 6 (orange).


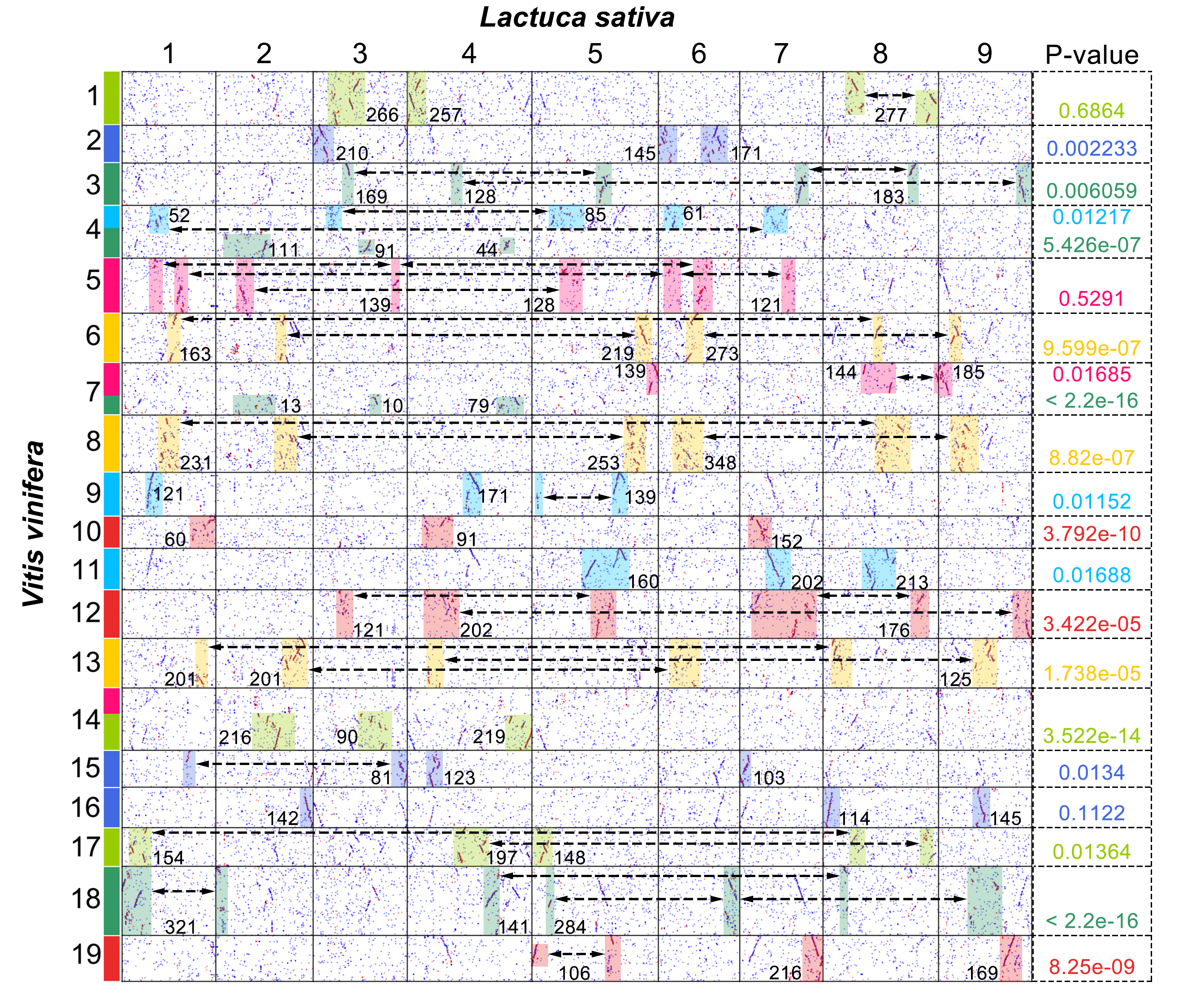


**Supplementary Figure S27. Intergenomic homologous structure comparison analyses between *V*. *vinifera* and *L*. *sativa* genomes.** The number of genes in three orthologous regions between *V*. *vinifera* and *L*. *sativa*. The highlighted frame indicates the identified orthologous synteny regions between genomes related to the species divergence. The dashed arrow indicates that the orthologous region belongs to the same subgenome. The numbers next to the rectangles are the *L*. *sativa* remained number of gene in that region. The number on the right of the figure is the P-value of the gene reservation number of the three *L*. *sativa* subgenomes corresponding to each ancestral chromosome region. The P-value describes whether the number of genes on the homologous fragment is significantly different in the three subgenomes. The P-value < 0.05 indicates that the significant difference in retention among the three subgenomes of *L*. *sativa* is credible.


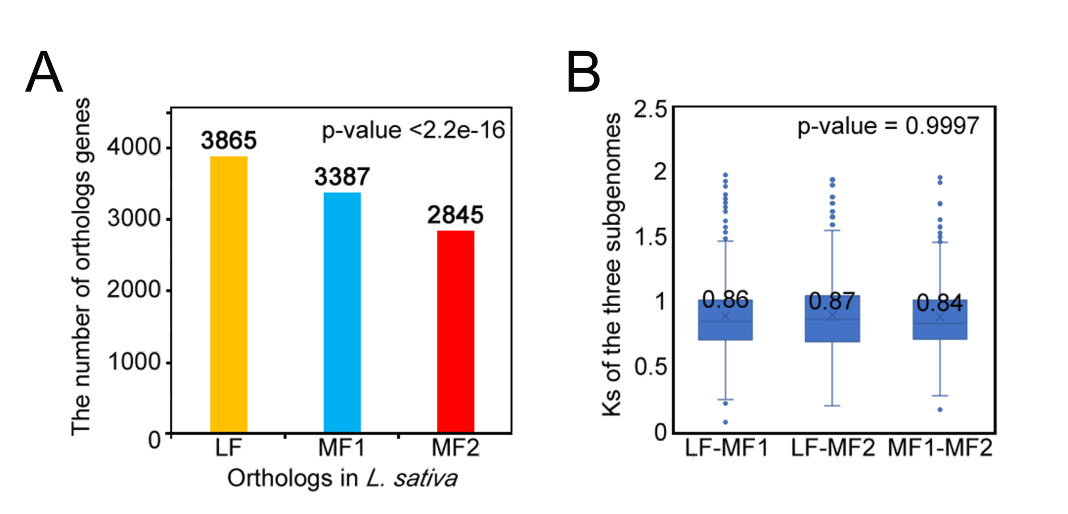


**Supplementary Figure S28. LF, MF1 and MF2 subgenomic gene retention numbers and inter-subgenomic *Ks*.** (A) The number of homologous genes between the three subgenomes in *L*. *sativa*. (B) The median *Ks* of the anchored gene pairs between the three subgenomes in *L*. *sativa*.


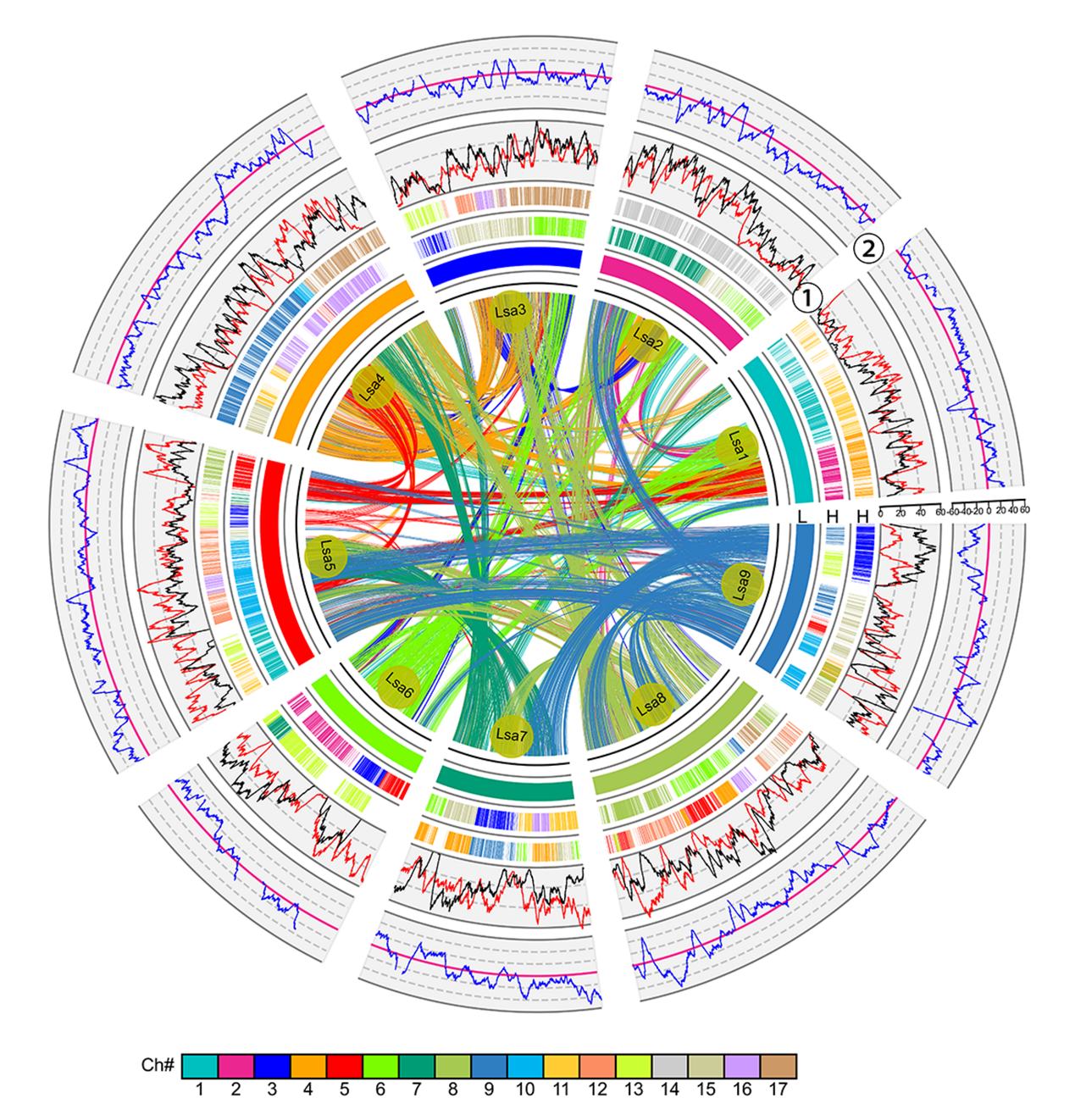


**Supplementary Figure S29. AST event in *H*. *annuus* generates intersubgenome retention level balance.** Genomic alignments and gene retention of *H*. *annuus* subgenomes along corresponding orthologous *L*. *sativa* chromosomes. The genes in 9 chromosomes of *L*. *sativa* within the inner circle colored by the 9 chromosomes of *L*. *sativa*, as shown in the color scheme at the bottom (denoted by Ch#). Compared to the *L*. *sativa* genome, the genomic paralogous and orthologous information within and among the genome of *H*. *annuus* in subgenomes is displayed in two circles. Each circle is formed by short vertical lines that denote homologous orthologous genes, which are colored to indicate the 17 *H*. *annuus* chromosome numbers in their respective source plant, as shown in the color scheme at the bottom (denoted by Ch#). In ①, gene retention level of homologous region group 1 (red) and 2 (black) in *H*. *annuus*, with *L*. *sativa* as the reference; in ②, difference in gene retention between homologous groups 1 and 2 (blue).

**
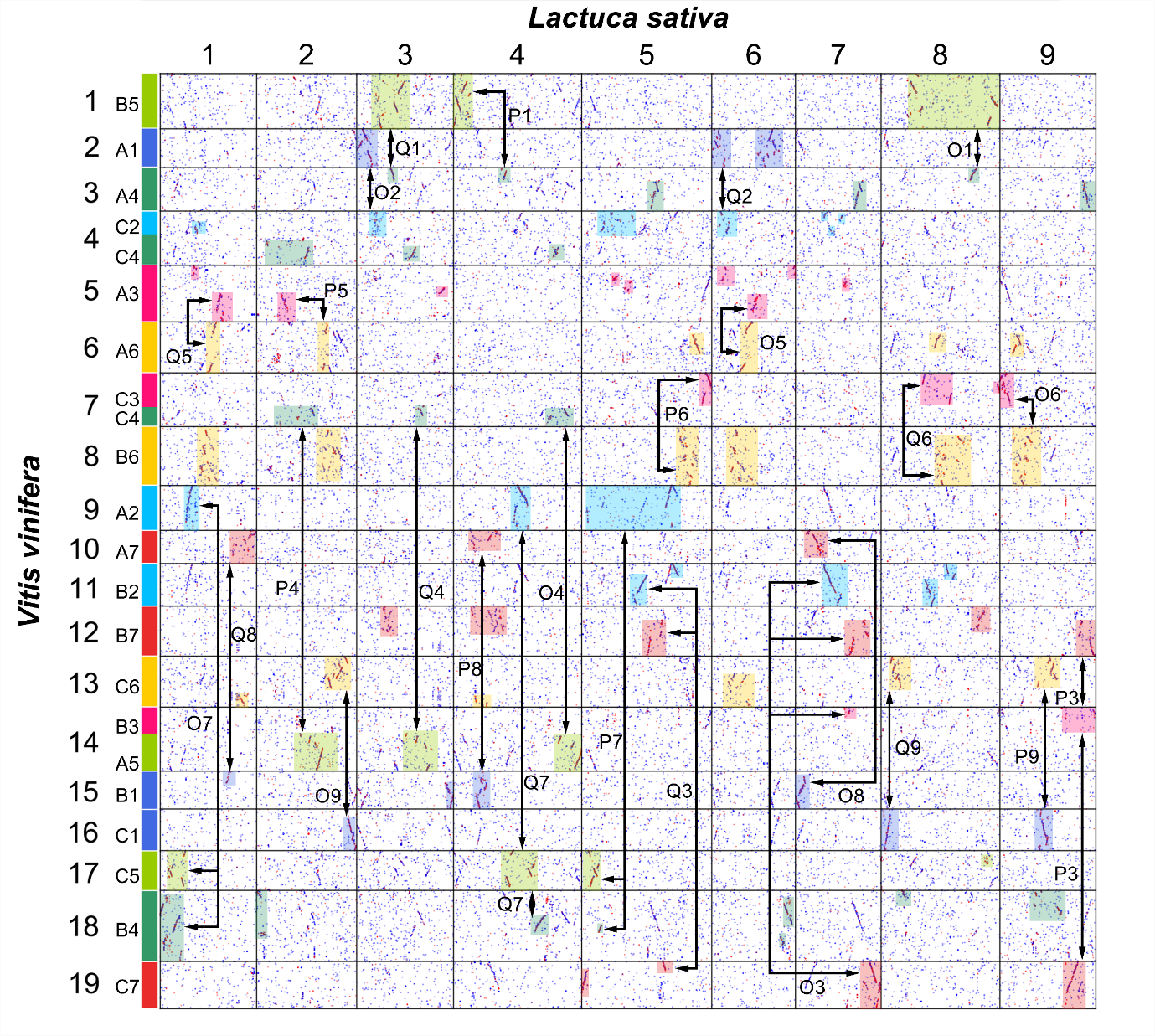
**

**Supplementary Figure S30. Ancestral chromosome fusion in the Asteraceae family inferred from *L*. *sativa*.** The color block next to the *V*. *vinifera* chromosome corresponds to the 7 ancestral chromosomes of the core eudicots, and labeled with numbers and letters. The highlighted frame indicates the identified orthologous synteny regions between genomes related to the species divergence. The chromosomal fusions connected by the black line and arrows are common to Asteraceae. The identified chromosomal fusions of the 21 chromosomes of the AEK (post-ECH event) contain 11 EEJ fusions and 1 NCF fusion formed AAK of 9 proto-chromosomes (T1-T9). After the ACH event, ancestral chromosomes T1-T9 were tripled as 27 chromosomes O1-O9, P1-P9, and Q1-Q9, which were corresponding to the moderately LF, MF1 and the MF2, respectively.


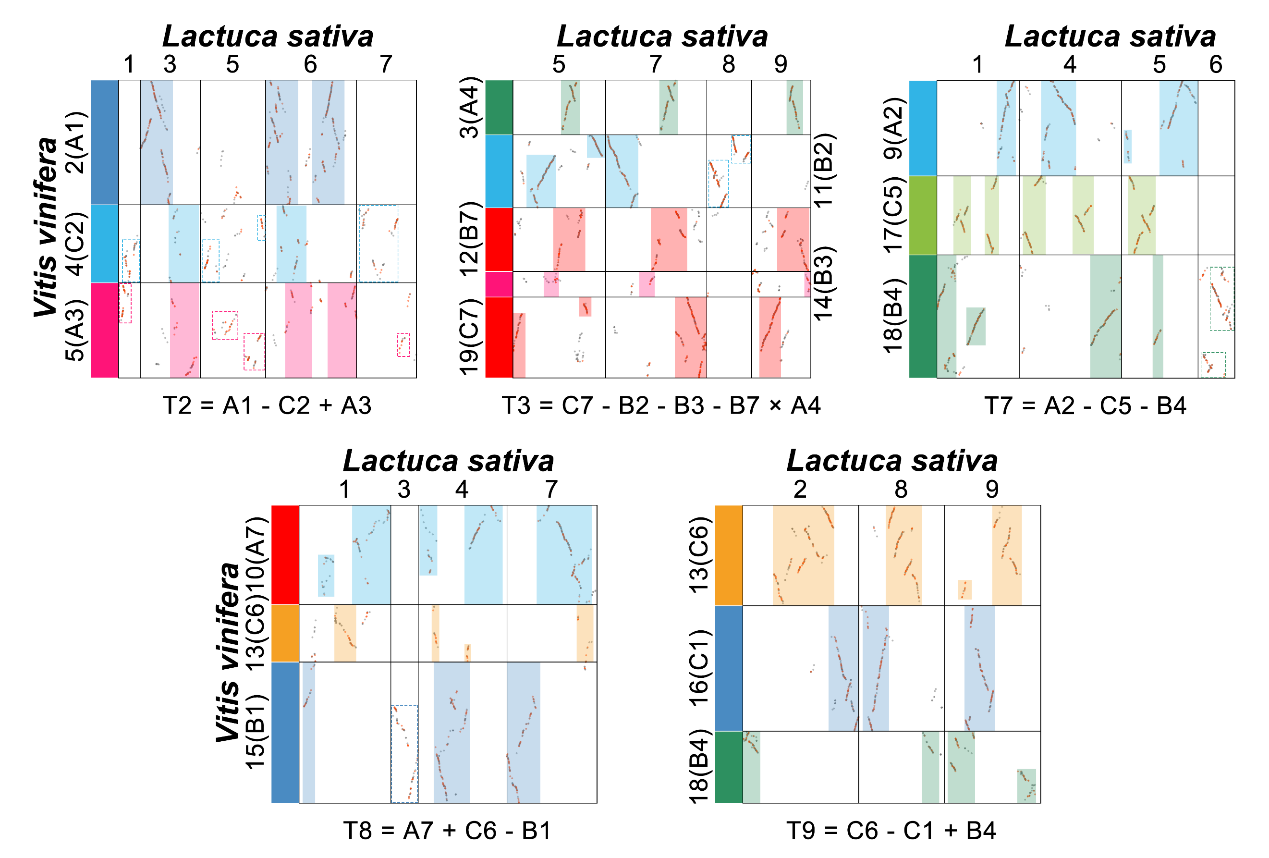


**Supplementary Figure S31. Construction of the ancestral karyotype of Asteraceae.** Local genomic syntenic block dot plots between *V*. *vinifera* and *L*. *sativa* showing the partial fusions of post-ECH event chromosomes before the ACH event. The 9 inferred proto-chromosomes in the ancestral Asteraceae genome are represented by T1-T9. Figure 4A has introduced T1, T4, T5 and T6, and only the remaining Asteraceae ancestral karyotype (AAK) are shown here. The parentheses following the *V*. *vinifera* chromosomes represent the 21 ancestral chromosomes from the post-ECH event. The three orthologous regions between *V*. *vinifera* are highlighted to correspond to the color of the seven ancestral chromosomes before the ECH. The fusion pattern is illustrated at the bottom of the dot plot, with “-” indicating chromosomal fusion (end-end joining [EEJ] and nested chromosome fusion [NCF]), “×” indicating reciprocal chromosomal translocation (RCT) and “+” indicating nonreciprocal chromosomal translocation (NCT). The identified chromosomal fusions of the 21 chromosomes of the ancestral eudicot karyotype (AEK, post-ECH event) contain 11 EEJ fusions, 1 NCF fusion, 2 RCTs, and 3 NCTs to form the AAK of 9 proto-chromosomes.

**
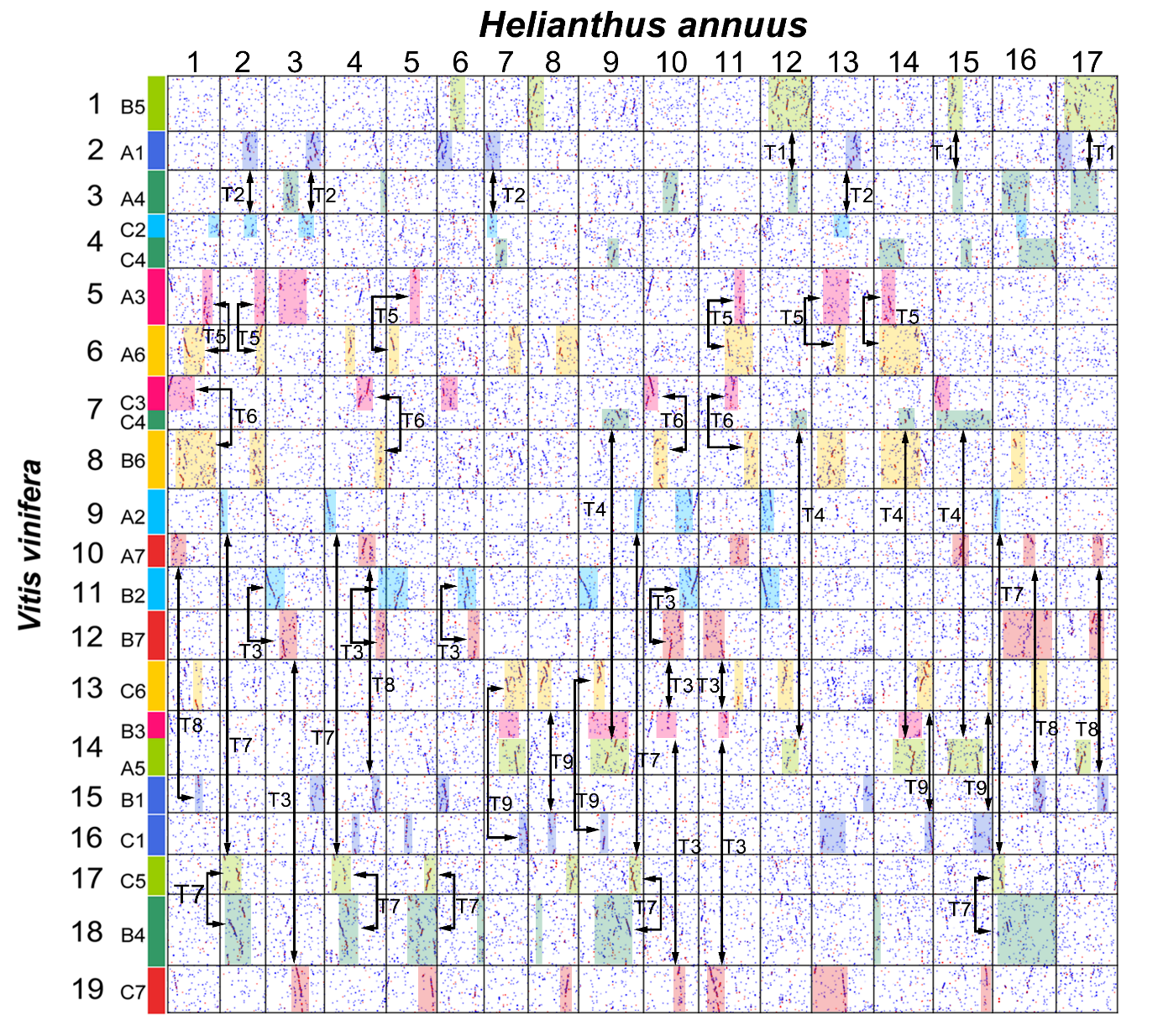
**

**Supplementary Figure S32. Ancestral chromosome fusion in the Asteraceae family inferred from *H*. *annuus*.** The color block next to the *V*. *vinifera* chromosome corresponds to the 7 ancestral chromosomes of the core eudicots, and labeled with numbers and letters. The highlighted frame indicates the identified orthologous synteny regions between genomes related to the species divergence. The chromosomal fusions connected by the black line and arrows are common to Asteraceae. The identified chromosomal fusions of the 21 chromosomes of the ancestral eudicot karyotype AEK (post-ECH event) in post-ECH contain 11 EEJ fusions and 1 NCF fusion formed AAK of 9 proto-chromosomes (T1-T9).


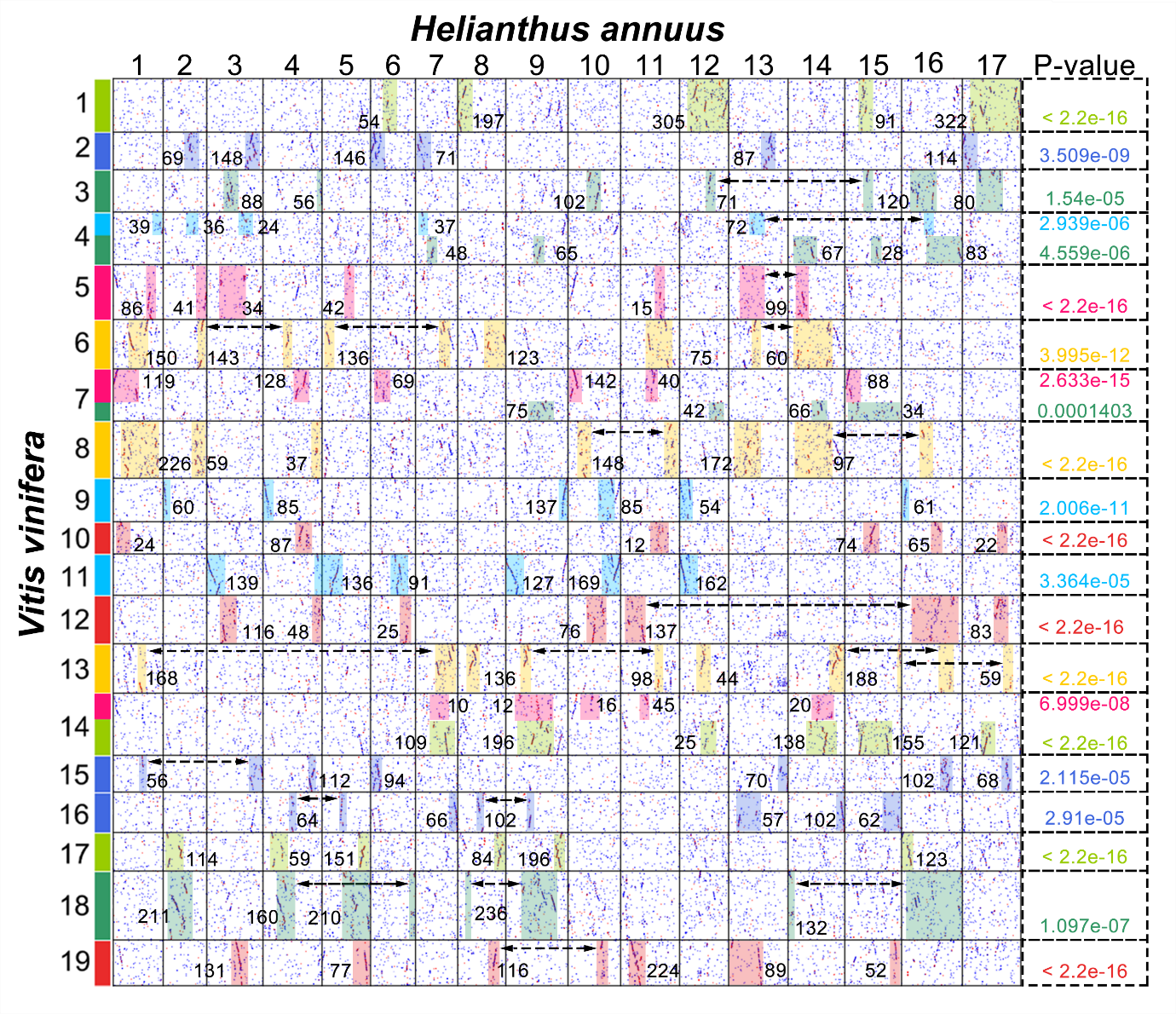


**Supplementary Figure S33. Intergenomic homologous structure comparison analyses between *V*. *vinifera* and *H*. *annuus* genomes.** The number of genes in six orthologous regions between *V*. *vinifera* and *H*. *annuus.* The highlighted frame indicates the identified orthologous synteny regions between genomes related to the species divergence. The dashed arrow indicates that the orthologous region belongs to the same subgenome. The numbers next to the rectangles are the *H*. *annuus* remained number of gene in that region. The number on the right of the figure is the p-value of the gene reservation number of the six *H*. *annuus* subgenomes corresponding to each ancestral chromosome region. The P-value describes whether the number of genes on the homologous fragment is significantly different in the six subgenomes. The P-value < 0.05 indicates that the significant difference in retention among the six subgenomes of *H*. *annuus* is credible.


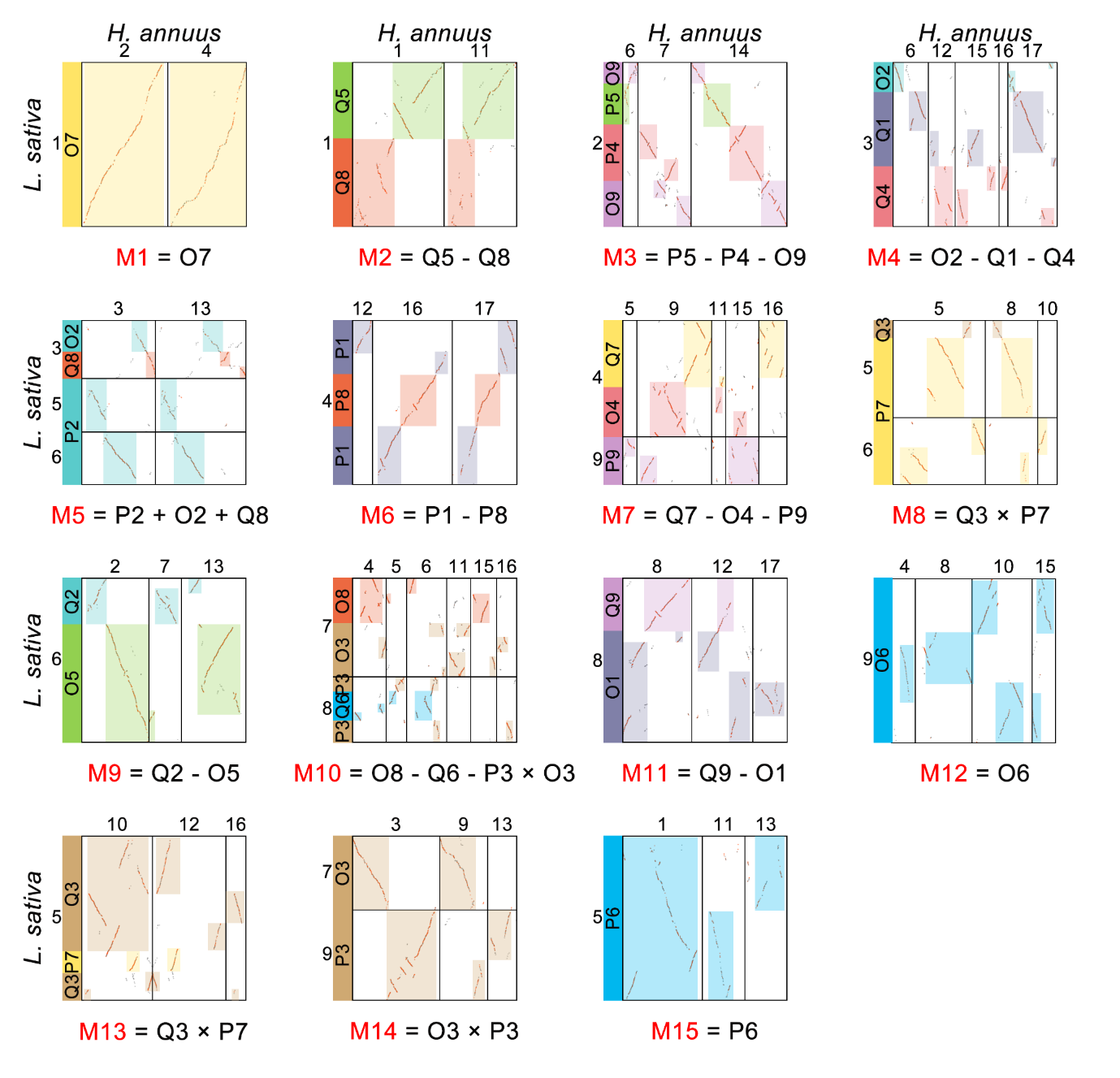


**Supplementary Figure S34. Construction of the most recent common ancestral karyotype of** **Asteraceae.** Local genomic syntenic block dot plots between *H*. *annuus* and *L*. *sativa* showing the after ACH events fusions. The 15 proto-chromosomes of the most recent common ancestor (MRCAK) inferred in the ancestral genome are represented by M1-M15. The rectangles following the *L*. *sativa* chromosomes represent the 27 ancestral chromosomes from the post-ACH event. The fusion pattern is illustrated at the bottom of the dot plot, with “-” indicating chromosomal fusions (EEJ and NCF), “×” indicating RCT and “+” indicating NCT. The identified chromosomal fusions of the 27 chromosomes of the AAK (post-ACH event) contain 10 EEJ fusions, 2 NCF fusions, 2 RCTs, and 2NCTs to form MRCAK of 15 proto-chromosomes.

**
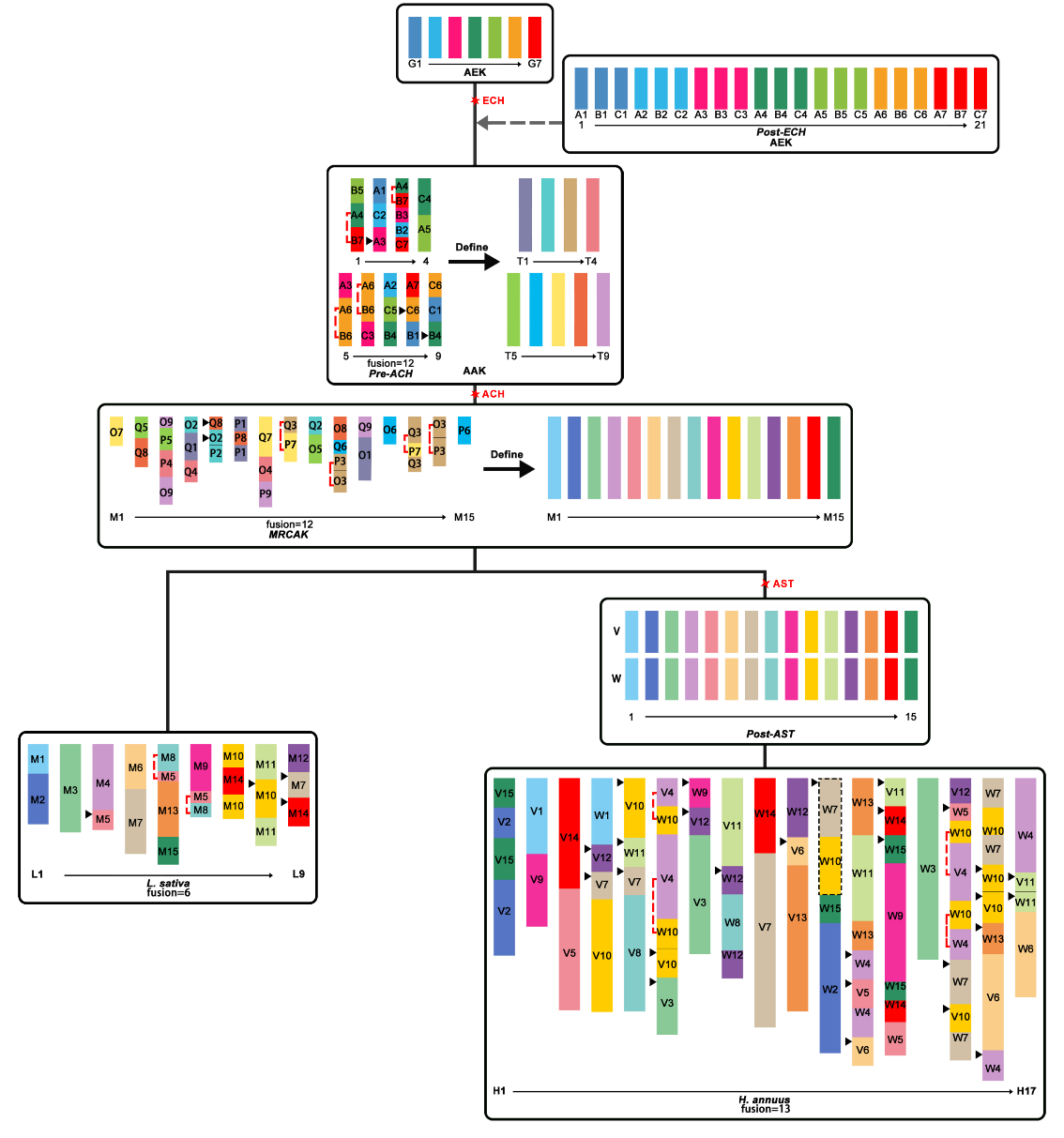
**

**Supplementary Figure S35. Reconstruction of the chromosome evolution trajectories of *L. sativa* and *H. annuus*.** The 9 inferred proto-chromosomes in the ancestral Asteraceae genome are represented by T1-T9. G1-G7 represent the ancient chromosomes (pre-gamma). A, B and C represent the three sub-genomes produced by the ECH event, while O, P and Q represent the three sub-genomes produced by the ACH event. V and W represent the two sub-genomes produced by the AST event. M1-M15 represent the most recent common ancestor (MRCAK) chromosomes of *L. sativa* and *H. annuus*. L1-L9 and H1-H17 represent the chromosomes of *L. sativa* and *H. annuus*, respectively. The black triangle indicates that the chromosomal fragment here was generated by NCT, and the red dashed lines indicates that the chromosomal fragment here underwent RCT. ECH and ACH events are marked with a red hexagonal star, and AST is marked with a red four-pointed star.


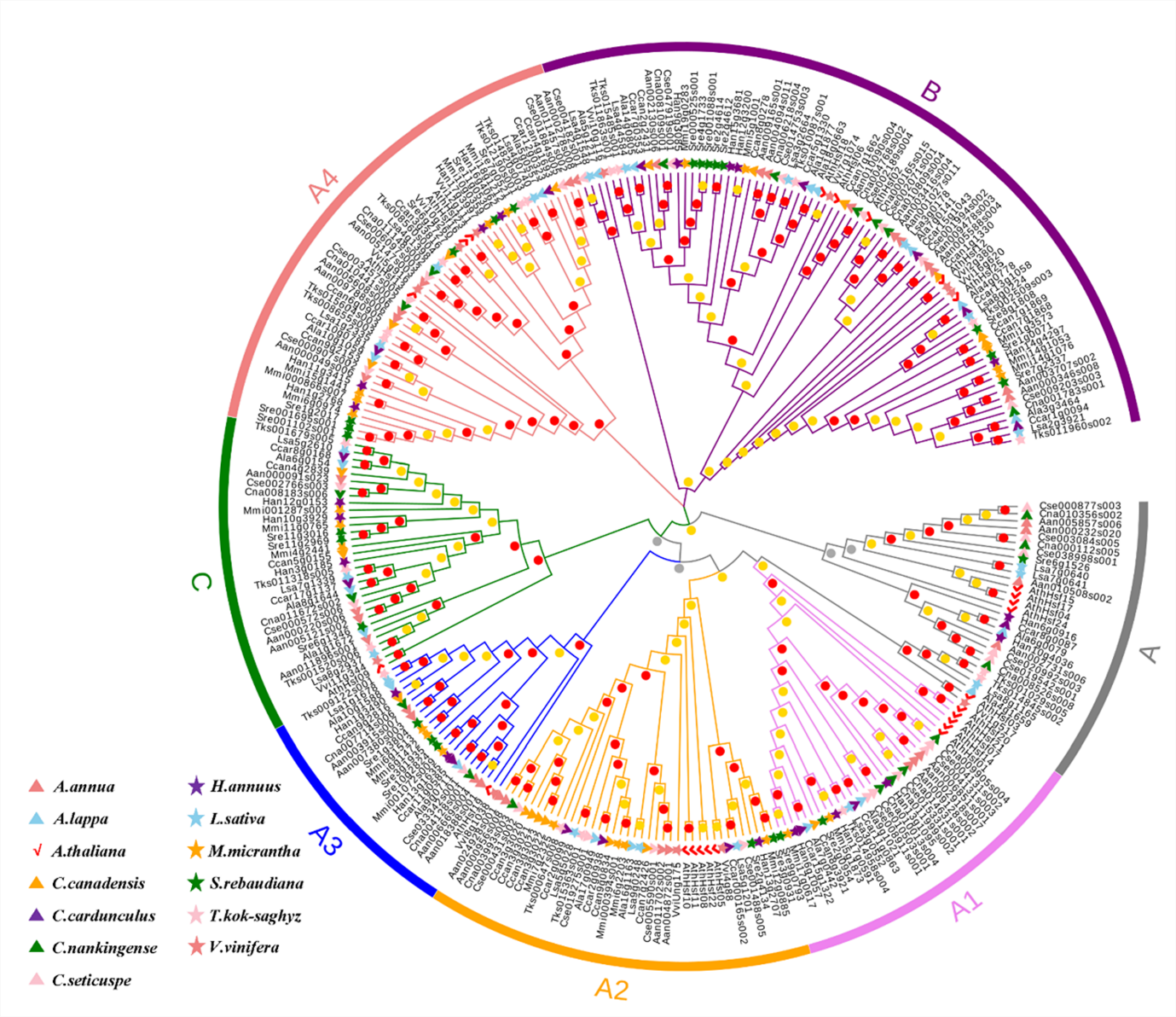


**Supplementary Figure S36. Inference of the expansion pattern of *Hsf* proteins by comparing the Asteraceae with the *V*. *vinifera* genes.** The bootstrap values that indicate the credibility levels of the structures are shown by the different color circles. The different symbols with different colors present the genes belong to different species. The “A” presents the A subgroup of *Hsf* proteins. The A1, A2, A3, A4, B, C represent the different subgroups. Moreover, for the expansion rates of the whole-genome and tandem duplication (WGD-ER and TD-ER) and contraction rates (CR) of the family in Asteraceae, supposing that the family of *V*. *vinifera* has N = [(total number of genes) – (number of the newly gained genes from tandem duplication)] genes, we designed the following algorithm:

(1) WGD-ER = (total number of the newly gained genes from polyploidizations / N) × 100%.

(2) TD-ER = (number of the newly gained genes from tandem duplication / N) × 100%.

(3) CR = (number of *V*. *vinifera* genes lost in Asteraceae / N) × 100%.

For A subgroup of *Hsf* genes, *L*. *sativa* has a duplication expansion rate 200% (whole-genome duplication expansion rate (*WGD-ER*): 100% and tandem duplication expansion rate (*TD-ER*): 100%, respectively), which is higher than that the contraction rate (*CR*) with 100%. These results suggested that all the whole-genome duplication, tandem duplication and gene loss influenced the expansion and contraction pattern of genes.


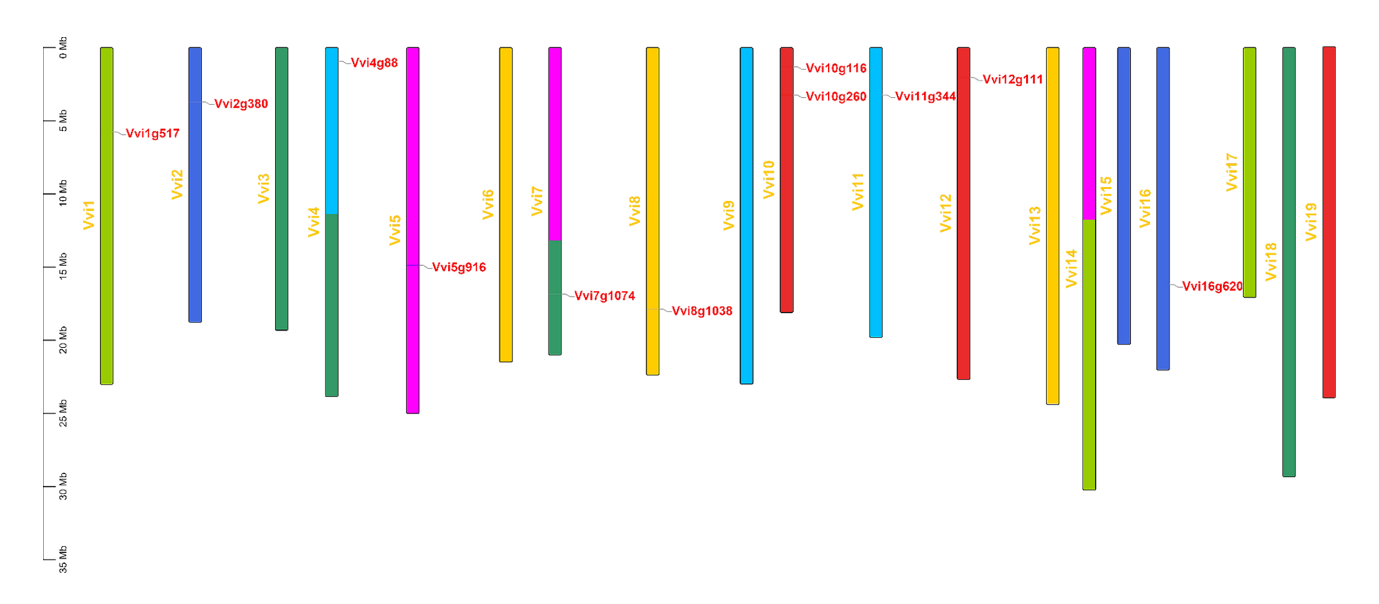


**Supplementary Figure S37. The chromosomal distributions of *Hsf* genes in *V*. *vinifera*.** The seven colors of the chromosomes correspond to the seven ancestral chromosomes of the core eudicots. The genes located on the same chromosome within a region (length <2Mb) formed a gene cluster. For this result, we found that the *Hsf* genes located on the chromosomes randomly.


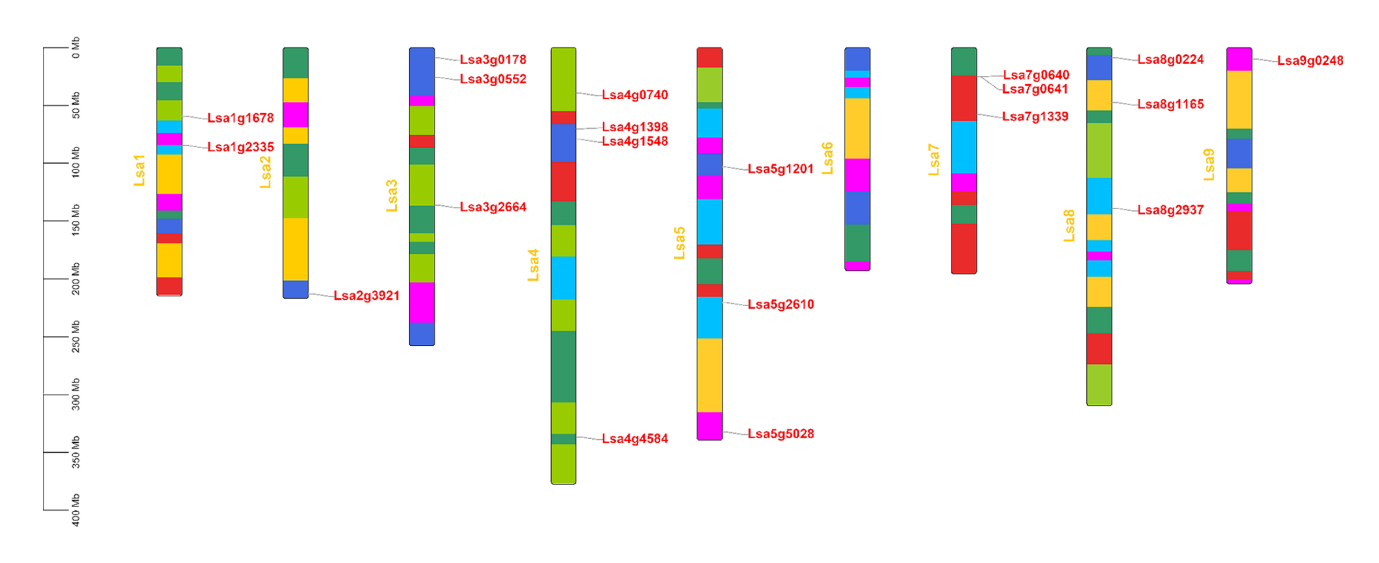


**Supplementary Figure S38. The chromosomal distributions of *Hsf* genes in *L*. *sativa*.** The seven colors of the chromosomes correspond to the seven ancestral chromosomes of the core eudicots. The genes located on the same chromosome within a region (length <2Mb) formed a gene cluster. For this result, we found that the *Hsf* genes located on the chromosomes randomly.


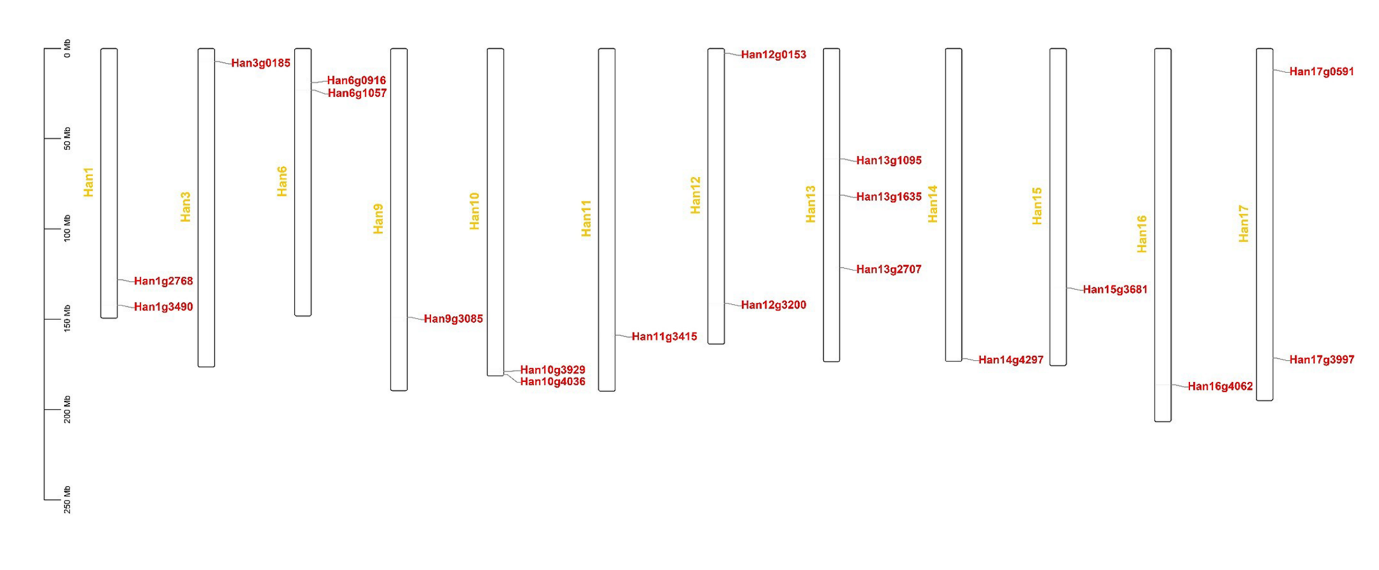


**Supplementary Figure S39. The chromosomal distributions of *Hsf* genes in *H*. *annuus*.** The seven colors of the chromosomes correspond to the seven ancestral chromosomes of the core eudicots. The genes located on the same chromosome within a region (length <2Mb) formed a gene cluster. For this result, we found that the *Hsf* genes located on the chromosomes randomly.

**
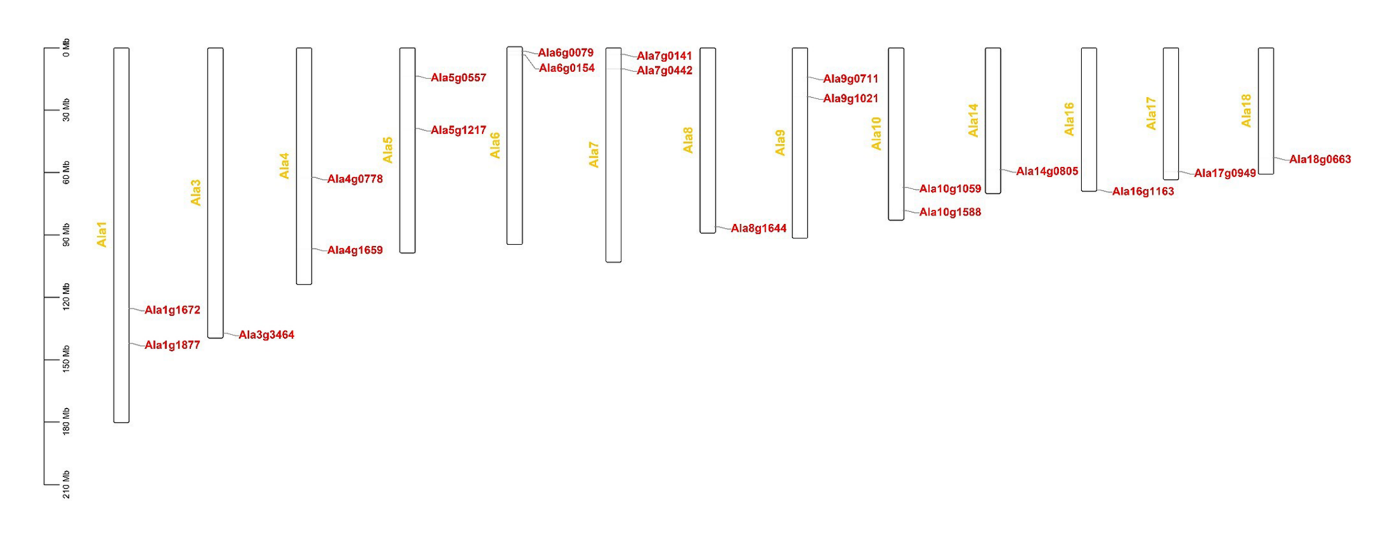
**

**Supplementary Figure S40. The chromosomal distributions of *Hsf* genes in *A*. *lappa*.** The seven colors of the chromosomes correspond to the seven ancestral chromosomes of the core eudicots. The genes located on the same chromosome within a region (length <2Mb) formed a gene cluster. For this result, we found that the *Hsf* genes located on the chromosomes randomly.

**
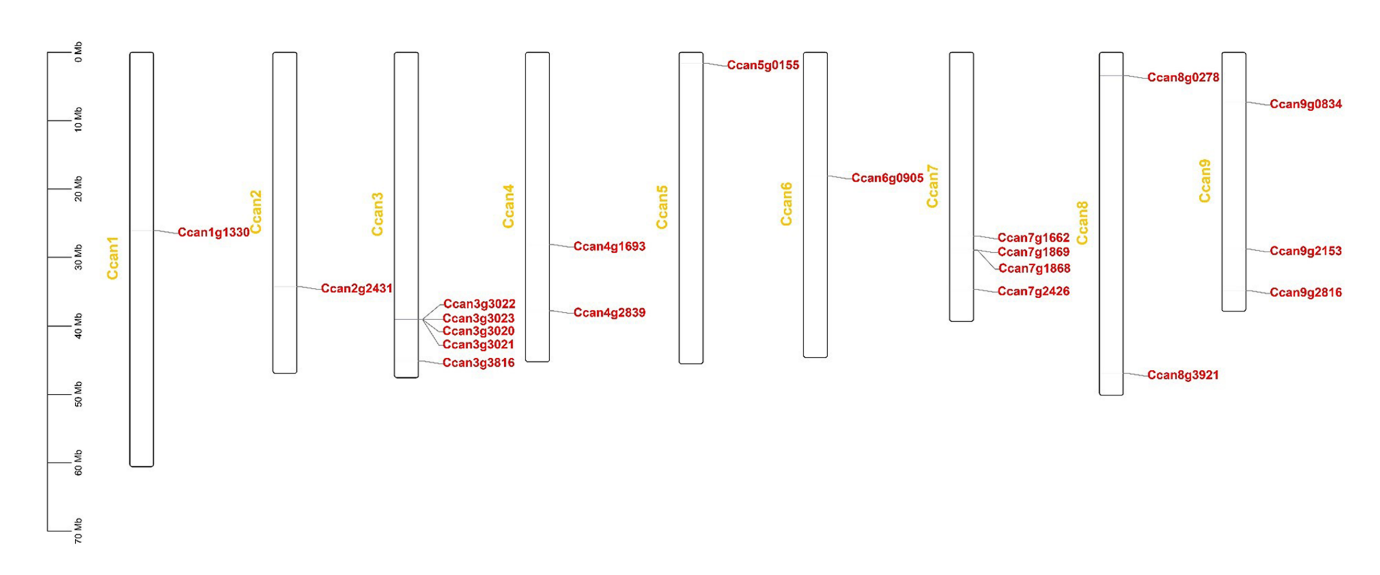
**

**Supplementary Figure S41****. The chromosomal distributions of *Hsf* genes in *C*. *canadensis*.** The seven colors of the chromosomes correspond to the seven ancestral chromosomes of the core eudicots. The genes located on the same chromosome within a region (length <2Mb) formed a gene cluster. For this result, we found that the *Hsf* genes located on the chromosomes randomly and part of *Hsf* genes formed the gene clusters, such as the genes on the chromosome 3 in *C*. *canadensis*.


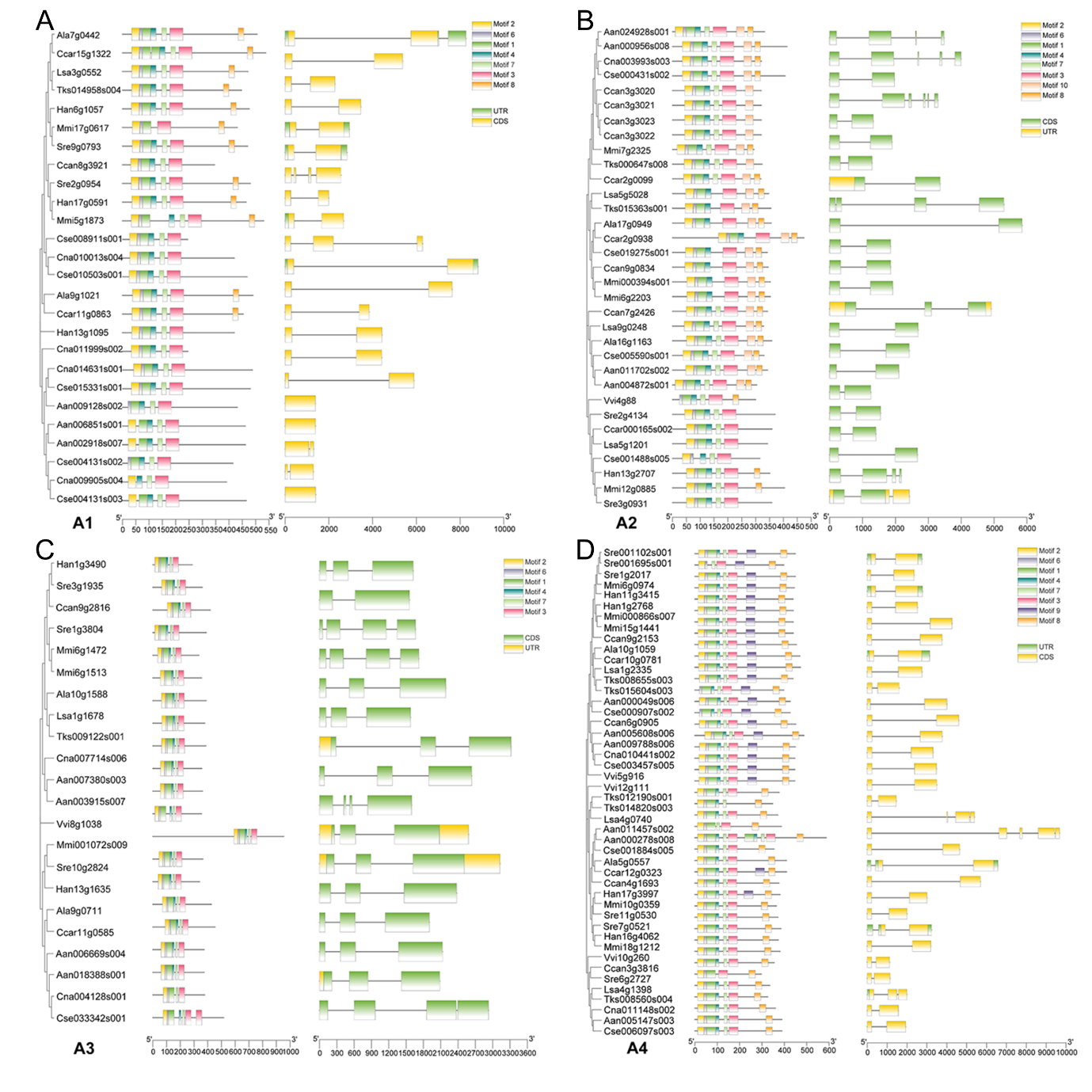


**Supplementary Figure S42. The phylogenetic trees and structure analyses of the *Hsf* genes within A1 (A), A2 (B), A3 (C) and A4 (D) subgroups in studied genomes.** The unit of the motif lens was aa and the unit of CDS and UTR was bp. For genes of A1, A2, A3, and A4 subgroups, the motif1-7 existed in each gene, the motif8 existed in each gene in A4, the part genes of A1 and A3 subgroup, and the motif10 only existed in genes of A2 subgroup, the motif9 only existed in genes of A4 subgroup. This result showed that the structure of *Hsf* genes is diverse.


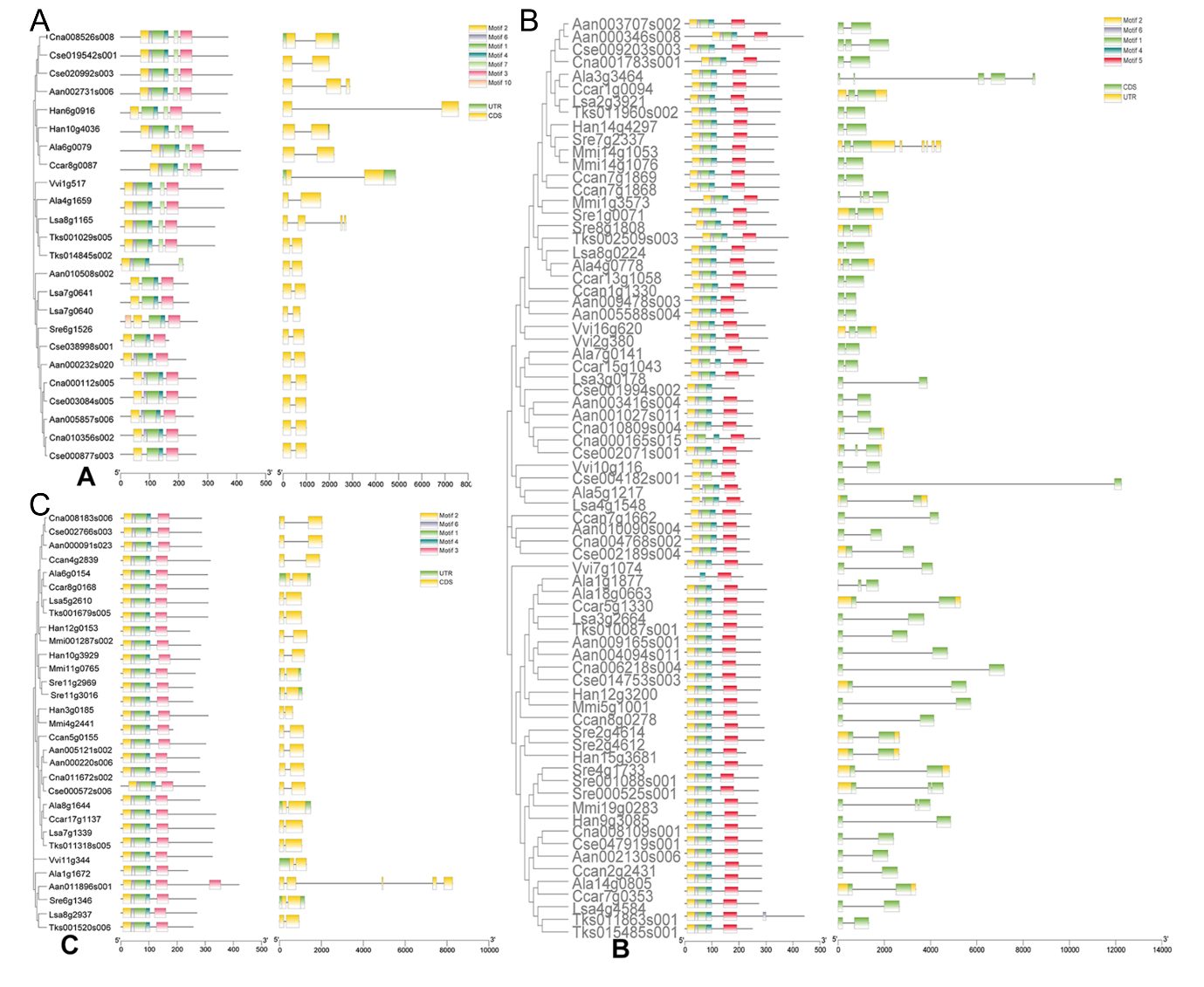


**Supplementary Figure S43. The phylogenetic trees and structure analyses of the *Hsf* genes within A (A), B (B) and C (C) subgroups in studied genomes.** The unit of the motif lens was aa and the unit of CDS and UTR was bp. For genes of A, B, and C subgroups, the motif1/2/4/6 existed in most genes, the motif3/7/10 existed in part genes of A, and the motif5 only existed in each gene of B subgroup, the motif3 only existed in each gene of C subgroup. This result showed that the structure of *Hsf* genes is diverse.


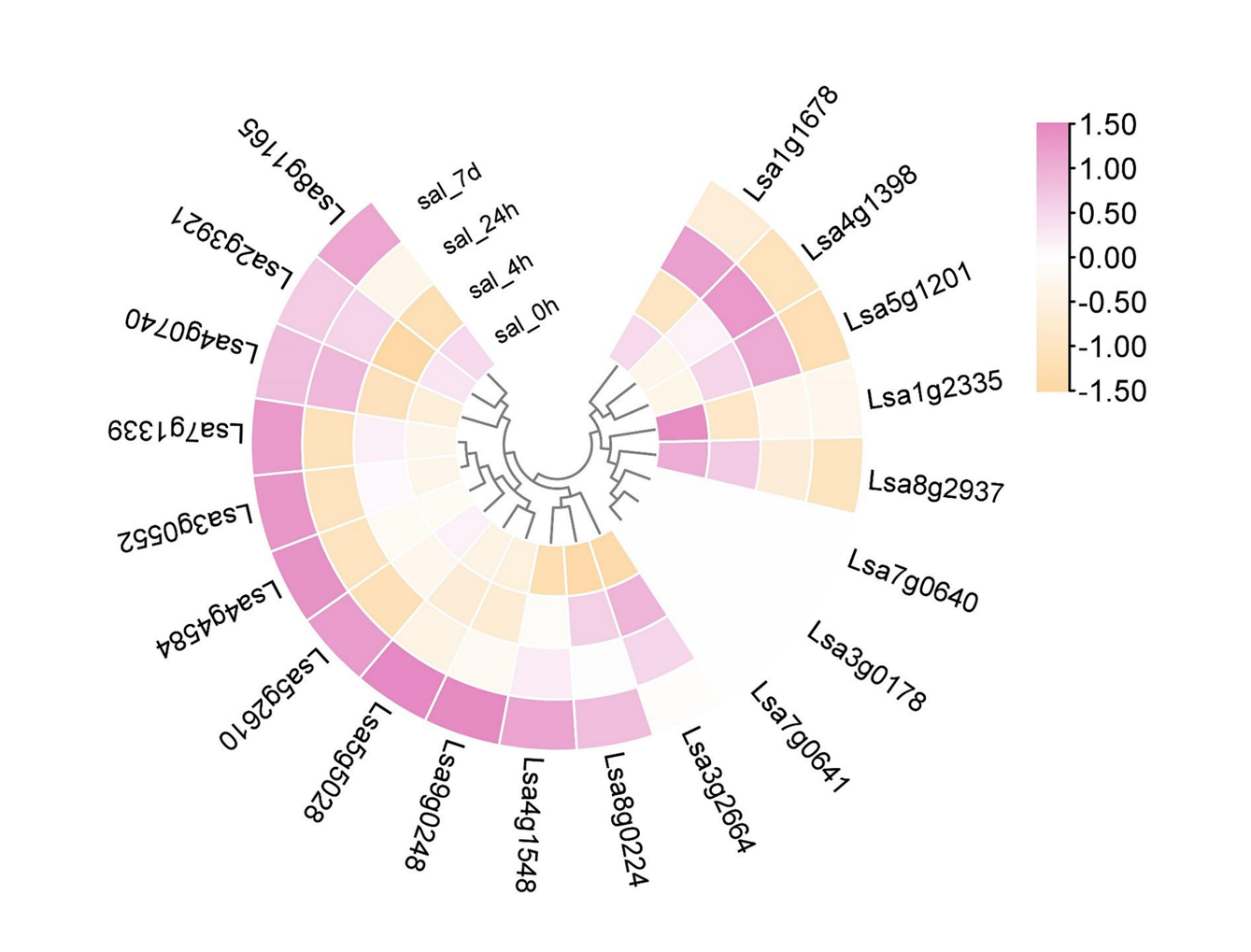


**Supplementary Figure S44. The expression pattern of *Hsf* proteins among *L*. *sativa*.** The color from blue to red represent the lower expression level to the higher expression level. The sal_0h, sal_4h, sal_24h and sal_7d represent the different tissues of *L*. *sativa*. This result represent that some *Hsf* genes not expressed in all the tissues, implying that the expression of *Hsf* genes in *L*. *sativa* may be influenced by the dosage balance constraints.
